# Pyridazino-1,3a,6a-Triazapentalenes as Versatile Fluorescent Probes: Impact of Their Post-Functionalization and Application for Cellular Imaging

**DOI:** 10.3390/ijms22126645

**Published:** 2021-06-21

**Authors:** Doina Sirbu, Nicolas Chopin, Ivana Martinić, Moussa Ndiaye, Svetlana V. Eliseeva, Marie-Aude Hiebel, Stéphane Petoud, Franck Suzenet

**Affiliations:** 1 Institut de Chimie Organique et Analytique—ICOA UMR7311, Rue de Chartres, CEDEX 2, 45100 Orléans, France; dosirbu@gmail.com (D.S.); nicolas.chopin@gmail.com (N.C.); moussa.ndiaye@univ-orleans.fr (M.N.); marie-aude.hiebel@univ-orleans.fr (M.-A.H.); 2 Centre de Biophysique Moléculaire CNRS UPR 4301, Rue Charles Sadron, CEDEX 2, 45071 Orléans, France; ivana.martinic@yahoo.com (I.M.); svetlana.eliseeva@cnrs-orleans.fr (S.V.E.)

**Keywords:** pyridazino-1,3a,6a-triazapentalenes, fluorophores, photostability, cell imaging, fluorescence

## Abstract

Pyridazino-1,3a,6a-triazapentalenes (PyTAP) are compact fused 6/5/5 tricyclic scaffolds which exhibit promising fluorescent properties. Chemically stable, they can be post-functionalized using standard Pd-catalyzed cross-coupling chemistry. Several original PyTAP bearing additional unsaturated substituents in positions 2 and 8 were synthetized and their spectroscopic properties analyzed. They have been successfully tested as fluorescent probes for cellular imaging.

## 1. Introduction

**Scheme 1:**
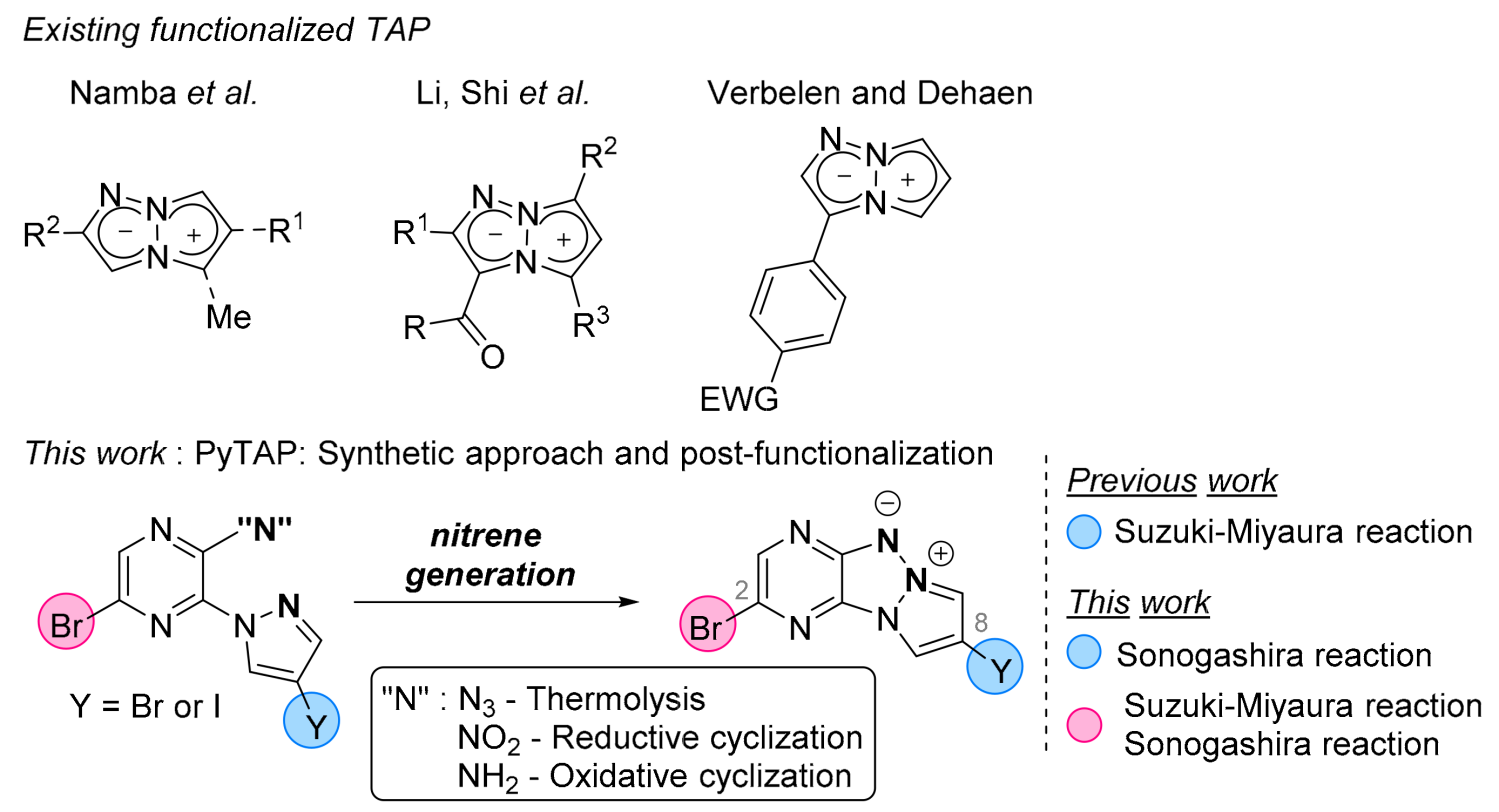
TAP and PyTAP scaffolds.

Small-organic fluorophores have become tools of major importance for biological, biochemical and biomedical research but also for diagnosis [1,2,3]. Recently added in this field, 1,3a,6a-triazapentalene (TAP) has emerged and its uses as probes [4,5,6] or sensors [7] has been tested. Therefore, extensive synthetic studies have been reported on the 5/5 bicyclic scaffold in order to introduce structural diversities in order to improve its fluorescent property and/or its chemical stability [8]. However, the postfunctionalization remains delicate and thus the modulation of the existing TAP fluorophore is closely related to the few synthetic pathways reported in the literature (Scheme 1), i.e., substitution at position 2 and 4 [9], at position 2 [10], position 2 and 5 [11,12], at position 4 [13], at position 2, 6 [14], at position 3 [15,16] and at position 2, 3, 4 and 6 [17]. Recently, the development of a TAP system flanked with an additional fused heteroaromatic ring demonstrated a significant improvement in respect to the chemical stability while retaining promising spectroscopic properties [18,19]. This low molecular weight tricyclic framework is straightforwardly synthetized from either azides [18] or nitro derivatives [20,21] though a thermal activation or primary amines, ref. [19] followed by a mild oxidative cyclization in the presence of an hypervalent iodine. A preliminary postfunctionalization experiment was already reported since several aryl-substituted triazapentalenes were obtained through a Suzuki–Miyaura cross-coupling reaction [18]. Here, we investigate further the reactivity and the stability of these pyridazino-1,3a,6a-triazapentalene (PyTAP) toward Pd-catalyzed cross-coupling chemistry and other postfunctionalization. The spectroscopic properties of each compound resulting from these structural refinements was analyzed. The most promising compounds were tested for their ability to be used as fluorescent probes for the imaging of living HeLa cancer cells.

Previous investigations enabled the formation of halogenated PyTAP in position 2 and 8 (**1a**–**c**) [18,19]. These key functional groups open the way to additional chemical transformations such as Pd-catalyzed cross-coupling reaction. While the presence of aromatic groups in the position 8 of PyTAP were expected to shift the absorption and emission wavelengths to the red with an increased molar absorption coefficient, a previous study shown that the π-conjugation was inexistent and that the spectroscopic properties of the PyTAP scaffold were almost unchanged [18]. Therefore, taking advantage of a halogenated PyTAP to perform various Suzuki–Miyaura or Sonogashira reactions enabled the formation and exploration of new type of π-conjugated system (i.e., alkynes) and new substitution positions on the PyTAP scaffold though simple chemical modification. Fluorescence properties were substantially improved in respect to brightness and/or to photostability.

## 2. Results and Discussion

**Scheme 2:**
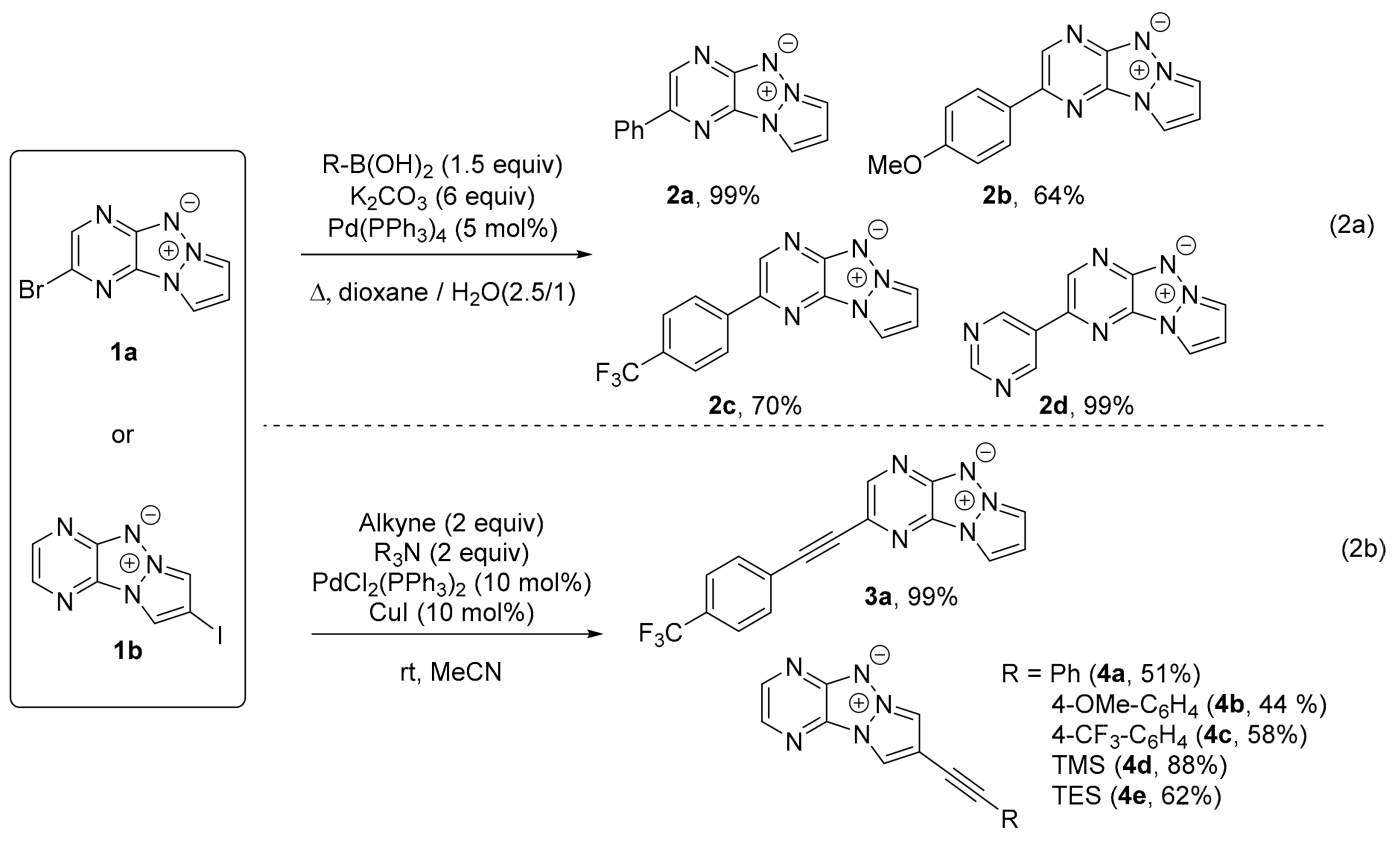
(**2a**) Formation of 2- and 8-substituted PyTAP through Suzuki-Miyaura cross-coupling reactions. (**2b**) Formation of 2- and 8-substituted PyTAP through Sonogashira cross-coupling reactions.

First, four derivatives were obtained in good to excellent yield when **1a** was submitted to a Suzuki–Miyaura reaction following usual reaction conditions (Scheme 2). By this way, phenyl- (**2a**), 4-methoxyphenyl- (**2b**), 4-trifluoromethyphenyl- (**2c**) and pyrimidin-5-yl-PyTAP (**2d**) were straightforwardly isolated. For the introduction of an ethynyl moiety, the position 2 appeared more reactive than the position 8, where moderate yields were observed (**3a** vs. **4a**–**e**) except for the compound **4d**. In general, arylacetylenes **4a–c** provided the expected product in a lower yield compared to trialkylsilyl ethnyl and alkynyl, **4d**–**e** and **4g**, **4i** respectively. However, by this approach, a selection of electron withdrawing and donating groups were introduced and their impact on the spectroscopic properties were evaluated.

**Scheme 3:**
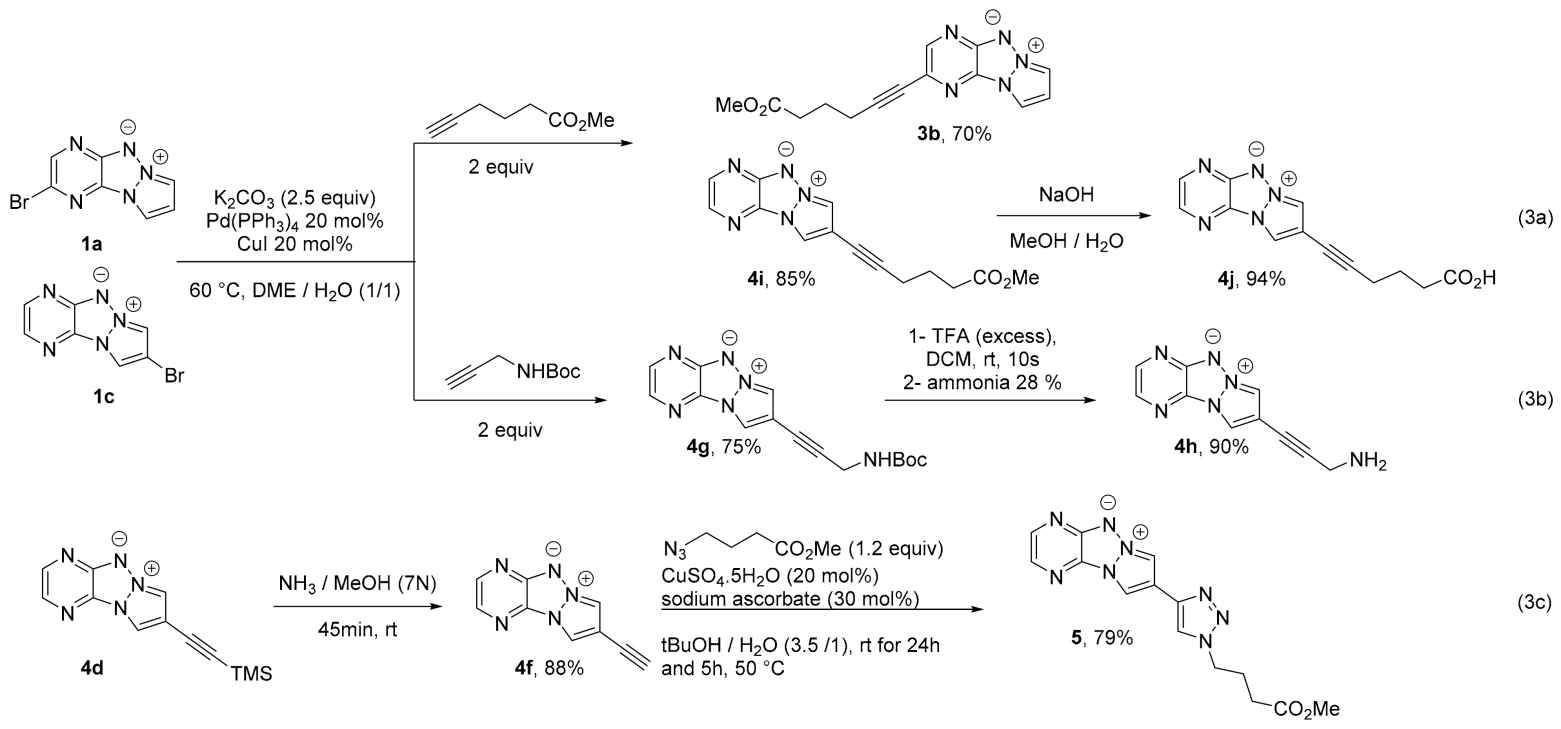
Introduction of ester (**3a**) and aminated (**3b**) groups through Sonogashira cross-coupling reactions. Introduction of ester (**3c**) group through a CuAAC reaction.

Finally, various alkynyl bearing alkyl chains were introduced through a Sonogashira reaction from starting materials **1a** and **1c** with Pd(0) and CuI as catalyst in a mixture of DME and water (1/1) (Scheme 3). Under these reaction conditions, Boc-protected amine and ester were well tolerated in position 2 and 8 on the PyTAP scaffold (**4g**, **3b** and **4i**). A further Boc removal and saponification were successfully performed to provide **4h** and **4j** respectively in excellent yields. This final reaction underlined the chemical stability of the PyTAP scaffold either under basic or acidic conditions.

Taking advantage of the efficient introduction of the trimethylsilyl ethnyl moiety (**4d**), an alternative synthetic route was explored (Scheme 3). The smooth deprotection of the alkyne was achieved with a saturated methanolic solution of ammonia, giving access to **4f** in an excellent yield. At this stage, a copper (I)-catalyzed azide–alkyne cycloaddition (CuAAC) was successfully performed with **4f** giving **5** in a 79% yield. Beyond the success of this cycloaddition, this sequence illustrates the possibility to use **4f** in CuAAC reaction with various linkers in bioconjugation strategies.

In the meantime, it is worth noting that the terminal alkyne reacted almost quantitatively in a Sonogashira reaction providing **4a** in a 74% global yield from **1b** vs. 51% considering the direct conversion (see Supplementary Materials for details).

With these compounds in hand, the spectroscopic properties of the resulted functionalized PyTAP were analyzed (Table 1). Concerning the position 8, we noticed that accordingly to what was suspected in the previous studies with the presence of aromatic or heteroaromatic substituents, ref. [18] the introduction of alkynyl groups barely affects the excitation and emission wavelengths as well as the brightness of the compounds **4**. This result confirms a lack of electronic delocalization between the PyTAP scaffold and unsaturated substituents in this position. However, a bathochromic effect was observed with either aromatic (**2a**–**d**) or ethynyl (**3a**–**b**) groups in position 2. Apart from the quantum yield, which remained around a value of 0.50, a significant increase of the molar absorption coefficient was observed. This tendency is particularly emphasized for the compound **3a** vs. **4c**, the molar absorption coefficients of which are respectively 21,957 and 14,897 L·mol^−1^·cm^−1^. Satisfactorily, the presence of functional groups enabling further bioconjugation, i.e., carboxylic acid (**4j**) primary amine (**4h**), were well tolerated and the resulted PyTAP maintained their advantageous fluorescence properties.

To evaluate the potential of substituted PyTAPs as scaffolds for the design of fluorescent probes for cellular imaging, we analyzed the biological behavior and photostability of compounds **3a** (substituted in position 2) and **4i** (substituted in position 8) in epifluorescence microscopy experiment after determination of their cytotoxicity (Figures S1 and S2 in Supplementary Materials). The cytotoxicity was evaluated on HeLa cells incubated with different concentrations of **3a** or **4i** during 24 h using the Alamar Blue assay. No significant toxicity was observed up to 170 µM concentration.

**Figure 1:**
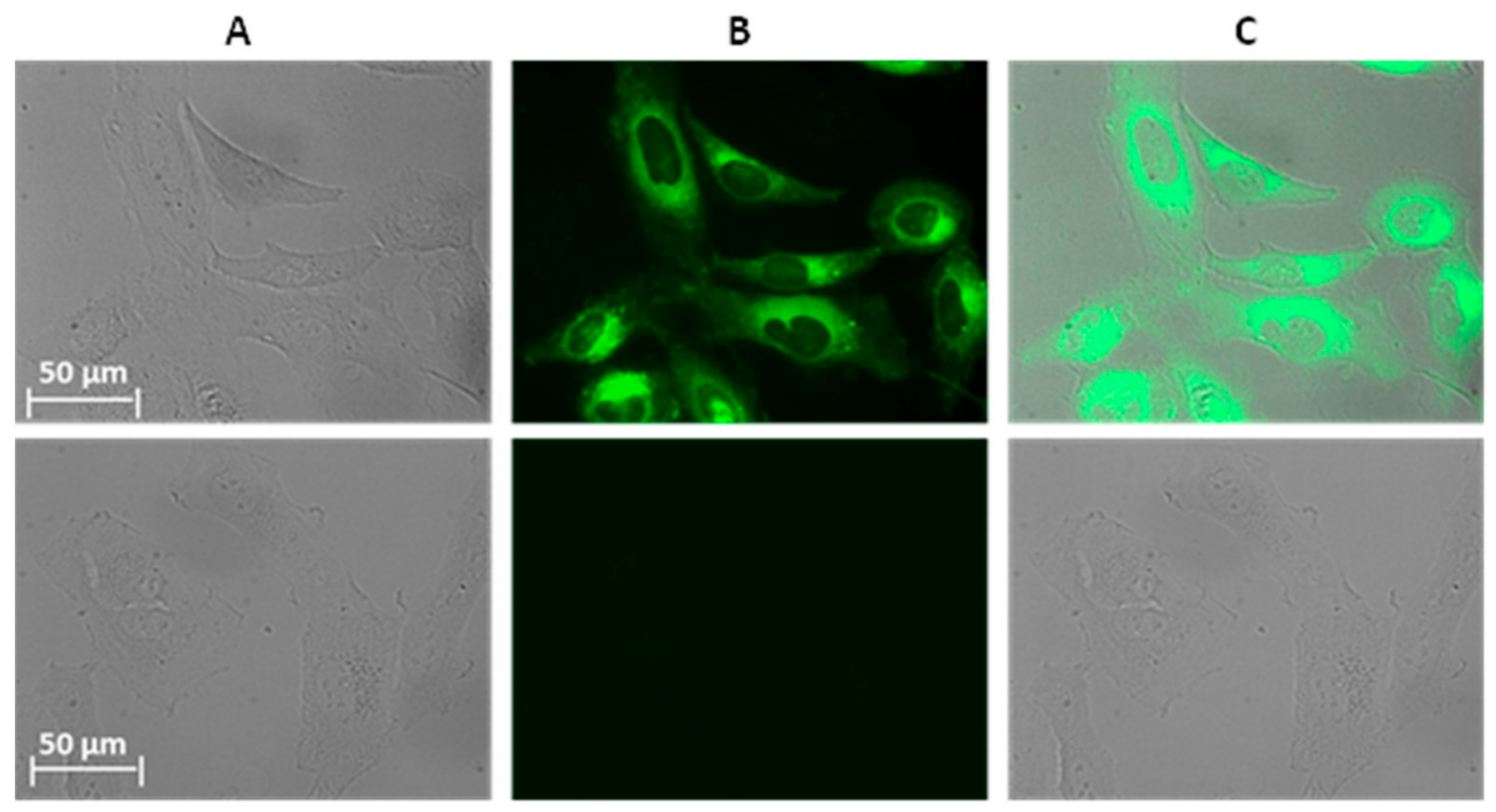
Images obtained from epifluorescence microscopy experiments on living HeLa cells. (Top) after 1 h and 30 min of incubation with 30 µM solution of the **3a** fluorescent probe. (Bottom) Untreated cells as a control. (**A**) Brightfield. (**B**) Fluorescence. *λ*_ex_: 485 nm band pass 20 nm filter, *λ*_em_: 525 nm band pass 50 nm, *τ*_exp_: 300 ms. (**C**) Merged. 40× objective.

**Figure 2:**
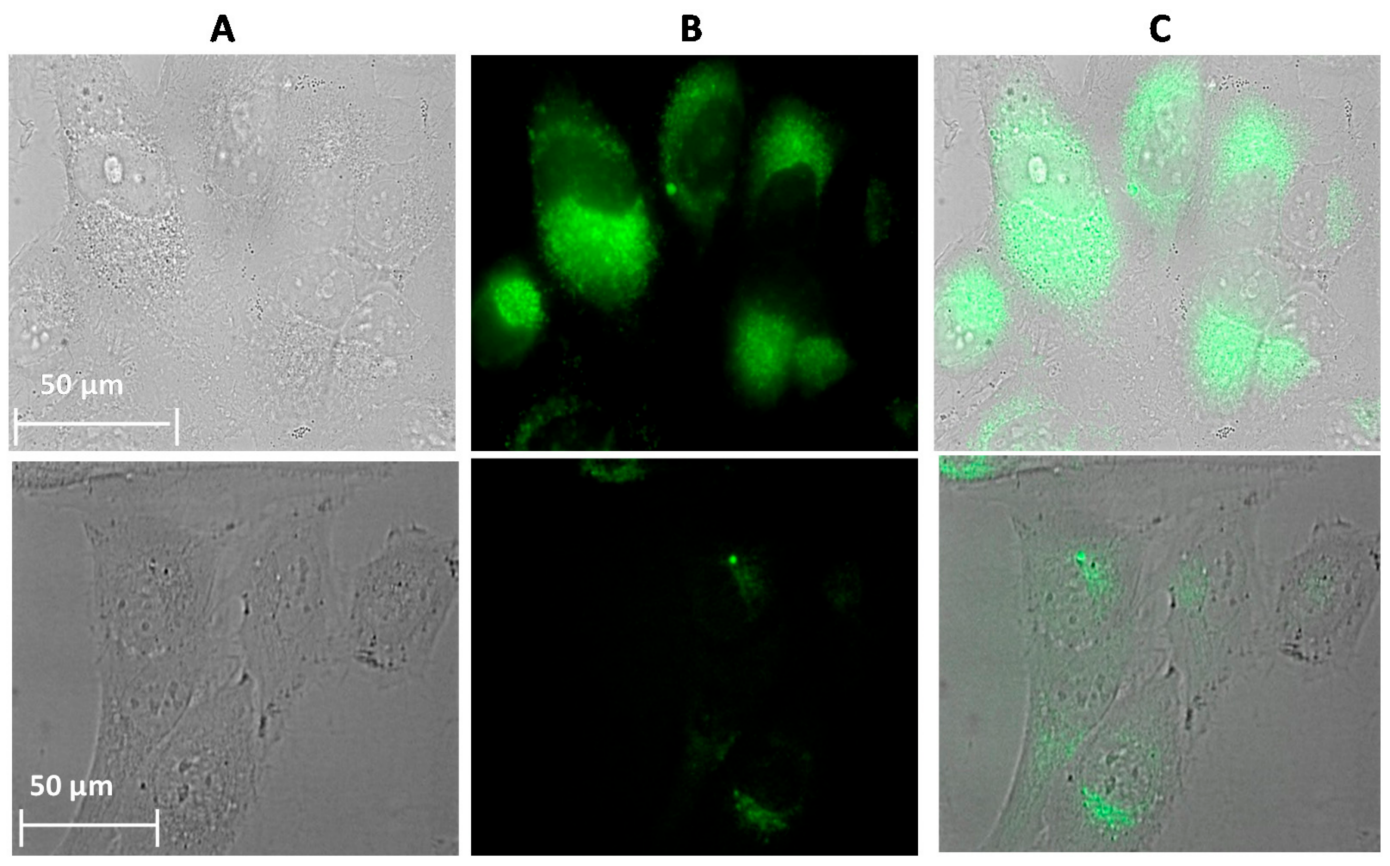
Results of epifluorescence microscopy experiments on living HeLa cells. (Top) after 1 h and 30 min of incubation with a 210 µM solution of the **4i** probe. (Bottom) Untreated cells as a control. (**A**) Brightfield. (**B**) Fluorescence. λex: 417 nm band pass 60 nm filter, λem: 536 nm band pass 40 nm, τexp: 500 ms. (**C**) Merged. 40× objective.

To perform epifluorescence microscopy experiments HeLa cells were incubated with **3a** (Figure 1) or **4i** (Figure 2) during 1 h 30 min. It was demonstrated that for both probes the emission signal arising from HeLa cells upon excitation with light selected using a 485 nm band pass 20 or 417 nm band pass 60 nm filters, respectively, could be clearly detected in the visible range.

**Figure 3:**
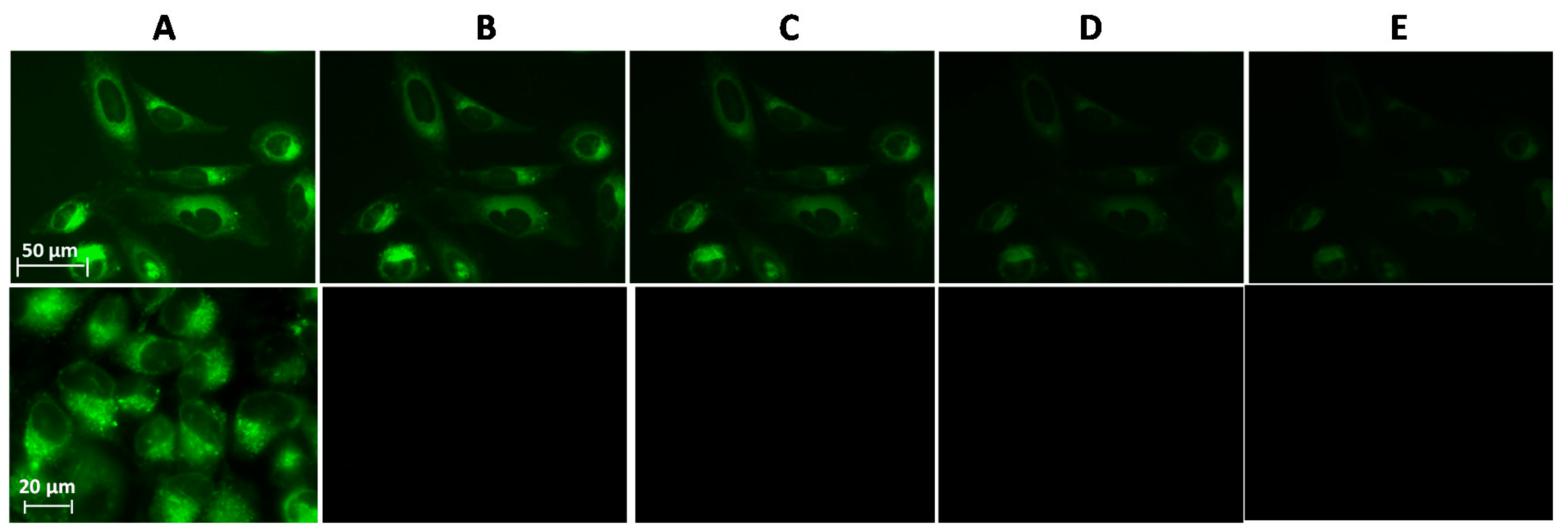
Images obtained from photobleaching experiments. HeLa cells incubated (top) during 1 h and 30 min with a 30 µM solution of the **3a** or (bottom) during 30 min with a 50 nM LysoTracker Green DND-26 after exposure to a continuous excitation light selected with a 485 nm (band pass 20 nm filter) collected at different times: (**A**) 0 s, (**B**) 25 s, (**C**) 45 s, (**D**) 100 s, (**E**) 185 s. The emission signal was collected with a 525 nm band pass 50 nm, *τ*_exp_: 300 ms, 40× objective.

**Figure 4:**
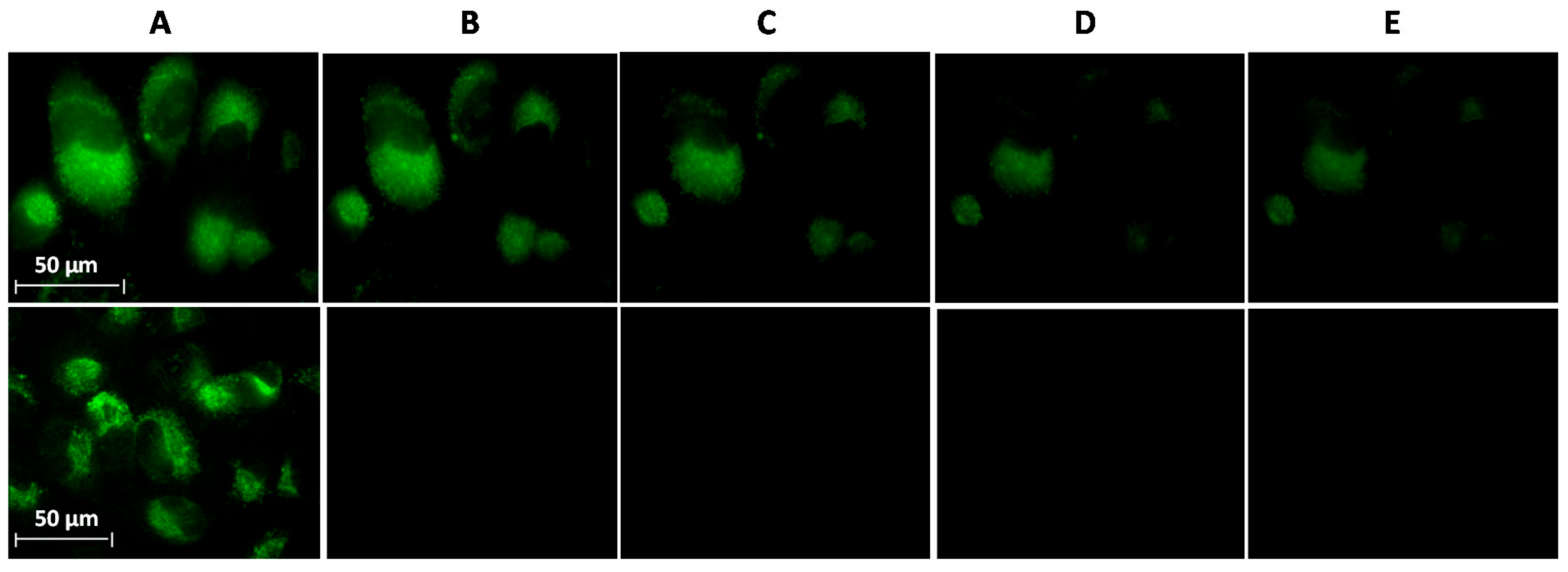
Images collected during photobleaching experiments. HeLa cells incubated (top) during 1h and 30 min with a 210 µM solution of the **4i** probe or (bottom) during 30 min with 50 nM LysoTracker Green DND-26 after exposure to a continuous excitation light selected with a 417 nm (band pass 60 nm filter) collected at different times: (**A**) 0 s, (**B**) 30 s, (**C**) 75 s, (**D**) 180 s, (**E**) 485 s. The emission signal was collected with a 536 nm band pass 54 nm, *τ*_exp_: 500 ms, 63× objective.

The photostability of the compounds **3a** and **4i** was evaluated by photobleaching experiments on HeLa cells upon continuous exposure to the excitation light selected with the appropriate band pass filters (Figure 3 and Figure 4 top panels). A comparison with the commercially available cellular imaging probe, LysoTracker Green DND-26, that exhibits excitation and emission in the similar range of wavelengths was performed (Figure 3 and Figure 4, bottom panels). These experiments confirmed an excellent and superior to LysoTracker Green DND-26 photostability of the PyTAP scaffold: an emission signal being still detectable after 100 or 485 s of continuous exposure to the excitation light.

## 3. Materials and Methods

### 3.1. Material and Methods

Unless otherwise noted, all reagent-grade chemicals and commercially available solvents were used without further purification. Reactions were monitored by thin-layer chromatography (TLC) using aluminum sheets coated with silica gel 60 F254. Flash column chromatography was carried out using silica gel 60 A° (0.04−0.06 mm). Solvents mentioned as being dry were purified with a dry station GT S100 immediately prior their use. NMR spectra were recorded with a 250 MHz (1H: 250 and 13C: 63 MHz), 300 MHz (1H: 300 and 13C: 75 MHz) or 400 MHz (1H: 400 and 13C: 100.7 MHz) Bruker spectrometer. Chemical shifts are given in parts per million (ppm) from tetramethylsilane (TMS), calibrated to the residual solvent peak. Coupling constants “J” are expressed in hertz (multiplicity: s = singlet, bs = broad singlet, d = doublet, dd = double doublet, dt = double triplet, t = triplet, q = quartet, m = multiplet). High-resolution accurate mass measurements (HRAM) were performed on a Bruker maXis mass spectrometer by the “Fédération de Recherche” ICOA/CBM (FR2708) platform. Melting points were measured in open capillary tubes.

### 3.2. Optical Spectroscopy

Absorption spectra were recorded on a UV-1800 Shimadzu spectrophotometer. Fluorescence measurements were performed with a Horiba Scientific Fluoromax-4 spectrofluorometer. Sample solutions were fully degassed by purging with argon.

Quantum Yield Measurements

The quantum yield of a dye is defined as follows:

φ = P_E_/P_A_

where, P_E_ and P_A_ are the number of photons emitted and absorbed respectively. Quantum yields of our tricyclic compounds were determined using an established relative method using coumarine 153 (λ_ex_ = 421 nm, λ_em_ = 531 nm in ethanol, φ = 0.38 in Ethanol) [22] as a reference.

The quantum yield of the compounds in organic solvents was calculated according to the following equation:

φ_sample_= φ_standard_ · (I_sample_/I_standard_) · (A_standard/_A_sample_) · (n_sample_/n_standard_)^2^

φ denotes the quantum yield; I denotes the area under the fluorescence band; A denotes the absorbance (in the range of 0.01–0.1 absorbance unit); n denotes the refractive index of the solvent (at 25 °C). All compound solutions were freshly prepared before each spectroscopic analysis, degassed with argon (during 30–40 min) and protected from direct light throughout the analysis. Fluorescence quantum yields (φ) were determined in DCM at room temperature upon selection of λ_ex_ and λ_em_ (maximum of emission) as the excitation and emission wavelengths.

### 3.3. General Procedures

#### 3.3.1. General Procedure A: Suzuki Miyaura Reaction

In a dry flask, the halogenated PyTAP (1 equiv) was solubilized in a 1,4-dioxane/water mixture (2.5/1; 30 mM) under argon. The corresponding boronic acid (1.5 equiv) was introduced with K_2_CO_3_ (6 equiv). The resulted reaction mixture was degassed with argon for 10 min before the introduction of Pd(PPh_3_)_4_ (0.05 equiv) and placed under reflux. After completion of the reaction (controlled by TLC), the reaction mixture was cooled to room temperature and concentrated under reduced pressure. The crude residue was purified by flash chromatography on silica gel.

#### 3.3.2. General Procedure B: Sonogashira Reaction

In a dry sealed tube, the halogenated PyTAP (1 equiv) was solubilized in dry acetonitrile under argon. Freshly distilled triethylamine (2 equiv) and the corresponding alkyne (2 equiv) were introduced. The resulted reaction mixture was degassed with argon for 10 min before the introduction of Pd(PPh_3_)_2_Cl_2_ (10 mol%) and CuI (10 mol%). The reaction was stirred at room temperature until completion of the reaction (controlled by TLC). The reaction mixture was concentrated under reduced pressure. The crude residue was purified by flash chromatography on silica gel.

#### 3.3.3. General Procedure C: Sonogashira Reaction

In a dry sealed tube, the halogenated PyTAP (1 equiv) was solubilized in a DME/water (1/1, 0.1 M). Potassium carbonate (2.5 equiv) and the corresponding alkyne (2 equiv) were introduced. The resulted reaction mixture was degassed with argon for 10 min before the introduction of Pd(PPh_3_)_4_ (20 mol%) and CuI (20 mol%). The reaction was stirred at 50–60 °C until completion of the reaction (controlled by TLC). The reaction mixture was concentrated under reduced pressure. The crude residue was purified by flash chromatography on silica gel.

### 3.4. Cell Imaging

#### 3.4.1. Epifluorescence Microscopy: Cellular Distribution

The HeLa (Human Cervical Carcinoma) cell line obtained from ATCC (Molsheim, France) was cultured in Dulbecco’s modified Eagle’s medium (DMEM) supplemented with 10% of heat-inactivated fetal bovine serum (FBS), 1% of 100× non-essential amino acid solution, 1% of L-glutamine (GlutaMAX) and 1% of streptomycin/penycilin antibiotics. Cells were seeded in an 8-well Lab Tek Chamber coverglass (Nunc, Dutsher S.A., Brumath, France) at a density of 6·10^4^ cells/well and cultured at 37 °C in a 5% humidified CO_2_ atmosphere. After 24 h, the cell culture medium was removed: Cells were then washed twice with Opti-MEM medium (room temperature) and incubated with pyTAP fluorescent probes during 1 h and 30 min at 30 µM concentration for **3a** or 210 µM concentration for **4i** during 30 min or during with a 50 nM LysoTracker Green DND-26. Prior to the epifluorescence imaging experiments, cells were washed twice with Opti-MEM (room temperature) in order to remove any non-specifically bound fluorescent probes. It should be noted that solutions of fluorescent probes were prepared in a DMSO and percentage of DMSO during epifluorescence microscopy was kept at 1%. Cells were observed with a Zeiss Axio Observer Z1 fluorescence inverted microscope (Zeiss, Le Pecq, France) equipped with a CCD camera (Orca-R2 Hamamatsu) with the help of the acquisition software Axiovision (Zeiss). The light source, Zeiss HXP 120 or Colibri, was combined with the following filter cubes: (i) 417 nm band pass 60 nm filter for the excitation and 536 nm band pass 40 nm filter for the emission; (ii) 485 nm band pass 20 nm filter for the excitation and 525 nm band pass 50 nm filter for the emission.

#### 3.4.2. Cytotoxicity Tests

Cytotoxicity tests were performed with the Alamar Blue^®^ assay (Invitrogen, France). Cells were seeded in 96-well plates at the density of 1·104 cells per well and cultured at 37 °C in a 5% humidified CO_2_ atmosphere. After 24 h of attachment, cells were incubated with different concentrations of pyTAP fluorescent probes (i.e., 25 nM, 75 nM, 100 nM, 30 µM, 60 µM, 95 µM, 130 µM, 150 µM and 170 µM) during 24 h followed by the incubation with the Alamar Blue^®^ (10% *v*/*v*) during 3–4 h at 37 °C in a 5% humidified CO_2_ atmosphere. Solutions of fluorescent probes were initially prepared in DMSO and the percentage of DMSO during cytotoxicity tests was maintained at 1%. The fluorescence of Alamar Blue^®^ was measured with a plate reader (Victor 3V, Perkin Elmer, Villebon-sur-Yvette, France) using a 530 nm excitation wavelength and collecting the emission at 590 nm. Control cells were prepared under the same experimental conditions but without the addition of the fluorescent probes but with 1% of DMSO. For each fluorescent probe three individual experiments were performed as triplicates. An average value out of these three experiments was calculated as a mean fluorescence value and corrected for the control cells. Data were presented as the mean ± RSD.

## 4. Conclusions

Here we underlined the possible post functionalization of the PyTAP scaffold in position 2 and 8 following standard Pd-catalyzed reaction conditions. By this way, alkynyl and aromatic substituents were smoothly and efficiently introduced in good to excellent yield. The resulted compounds highlighted the beneficial influence of the substitution in position 2 on the brightness of the fluorophore especially on the molar absorption coefficient. However, both substitutions (position 2 or 8) gave rise to nontoxic probes a high photostability, which further strengthen the versatility of the PyTAP scaffold as promising dye.

## Figures and Tables

**Table 1:** Spectroscopic characterization of functionalized PyTAP in CH_2_Cl_2_.

Dye	λ_ex_ ^a^ (nm)	λ_em_^b^ (nm)	ε_max_^c^	φ ^d^
2a	404	497	11,372	0.48
2b	448	503	18,449	0.38
2c	439	497	19,906	0.51
2d	440	502	15,416	0.58
3a	446	503	21,957	0.49
	445 ^e^	512 ^e^	20,237 ^e^	0.40 ^e^
3b	436	498	13,147	0.42
4a	417	485	16,800	0.58
4b	416	485	16,024	0.51
4c	417	479	14,897	0.58
4e	417	483	16,546	0.52
4f	415	481	17,697	0.53
4g	415	483	16,501	0.54
4h	419	478	13,200	0.46
4i	418	481	17,871	0.45
	418 ^e^	502 ^e^	14,058 ^e^	0.54 ^e^
4j	416	484	14,015	0.50
5	416	492	15,500	0.46

^a^ Apparent maxima of absorption bands. ^b^ Apparent maxima of emission band. ^c^ Units: L·mol^−1^·cm^−1^. ^d^ φ is the relative fluorescence quantum yield estimated by using coumarin 153 (φ = 0.38 in ethanol) as a reference standard [22]. **^e^** Measure performed in DMSO.

## Supplementary Materials

Spectral characterization and copies of ^1^H, ^13^C and ^19^F spectra for all compounds, spectroscopic analysis and cytotoxicity tests, are available online at https://www.mdpi.com/article/10.3390/ijms22126645/s1.
