# Supplementary material for: Pyridazino-1,3a,6a-Triazapentalenes as Versatile Fluorescent Probes: Impact of Their Post-Functionalization and Application for Cellular Imaging
Source: Int J Mol Sci. 2021 Jun 21;22(12):6645. doi: 10.3390/ijms22126645 (PMC8234658; doi:10.3390/ijms22126645)

Pyridazino-1,3a,6a-triazapentalenes as versatile fluorescent probes: impact of their post-functionalization and application for cellular imaging

Doina Sirbu, Nicolas Chopin, Ivana Martinić, Moussa NDiaye, Svetlana V. Eliseeva, Marie-Aude Hiebel, Stéphane Petoud, Franck Suzenet

Table of content

General information 1
General procedures and characterization data 2
Cell Imaging 7
Epifluorescence Microscopy: Cellular Distribution 7
Cytotoxicity tests 8
NMR SPECTRA 10
Photophysical measurements : absorption, excitation and emission spectra 47

General information

Material and methods

Unless otherwise noted, all reagent-grade chemicals (>95% purity) and commercially available solvents were used without further purification. Reactions were monitored by thin-layer chromatography (TLC) using aluminum sheets coated with silica gel 60 F254. Flash column chromatography was carried out using silica gel 60 Å (0.04–0.06 mm). Solvents mentioned as being dry were purified with a dry station GT S100 immediately prior their use. NMR spectra were recorded with a 250 MHz (^{1}H: 250 and ^{13}C: 63 MHz), 300 MHz (^{1}H: 300 and ^{13}C: 75 MHz) or 400 MHz (^{1}H: 400 and ^{13}C: 100.7 MHz) Bruker spectrometer. Chemical shifts are given in parts per million (ppm) from tetramethylsilane (TMS), calibrated to the residual solvent peak. Coupling constants "*J*" are expressed in hertz (multiplicity: s = singlet, bs = broad singlet, d = doublet, dd = double doublet, dt = double triplet, t = triplet, q = quartet, m = multiplet …). High-resolution accurate mass measurements (HRAM) were performed on a Bruker maXis mass spectrometer by the "Fédération de Recherche" ICOA/CBM (FR2708) platform. Melting points were measured in open capillary tubes.

Optical spectroscopy

Absorption spectra were recorded on a UV-1800 Shimadzu spectrophotometer. Fluorescence measurements were performed with an Horiba Scientific Fluoromax-4 spectrofluorometer. Sample solutions were fully degassed by purging with argon.

Quantum Yield Measurements

The quantum yield of a dye is defined as follows:

ϕ = PE/PA

Where P E,A are the number of photons absorbed and emitted respectively. Quantum yields of our tricyclic compounds were determined using an established relative method using Coumarine 153 (λ_{ex} = 421 nm, λ_{em} = 531 nm in ethanol, ϕ = 0.38 in Ethanol)[1] as a reference.

The quantum yield of the compounds in organic solvents was calculated according to the following equation:

$$\phi_{sample} = \phi_{standard} \cdot (I_{sample} / I_{standard}) \cdot (A_{standard} / A_{sample}) \cdot (n_{sample} / n_{standard})^2$$

ϕ denotes the quantum yield; I denotes the area under the fluorescence band; A denotes the absorbance (in the range 0.01 – 0.1 Absorbance unit); n denotes the refractive index of the solvent (at 25 °C). All compound solutions were freshly prepared before each spectroscopic analysis, degassed with argon (during 30-40 minutes) and protected from direct light throughout the analysis. Fluorescence quantum yields (ϕ) were determined in DMSO and DCM at room temperature upon selection of λ_{ex} and λ_{em} (maximum of emission) as the excitation and emission wavelengths.

[1]A. M. Brouwer, *Pure Appl. Chem.* **2011**, *83*, 2213–2228.

General procedures and characterization data

General procedure A: Suzuki Miyaura reaction

In a dry flask, the halogenated PyTAP (1 equiv) was solubilized in a 1,4-dioxane/water mixture (2.5/1; 30 mM) under argon. The corresponding boronic acid (1.5 equiv) was introduced with K_2CO_3 (6 equiv). The resulted reaction mixture was degassed with argon for 10 min before the introduction of Pd(PPh3)4 (0.05 equiv) and placed under reflux. After completion of the reaction (controlled by TLC), the reaction mixture was cooled to room temperature and concentrated under reduced pressure. The crude residue was purified by flash chromatography on silica gel.

General procedure B: Sonogashira reaction

In a dry sealed tube, the halogenated PyTAP (1 equiv) was solubilized in dry acetonitrile under argon. Freshly distilled triethylamine (2 equiv) and the corresponding alkyne (2 equiv) were introduced. The resulted reaction mixture was degassed with argon for 10 min before the introduction of $Pd(PPh_3)_2Cl_2$ (10 mol%) and CuI (10 mol%). The reaction was stirred at room temperature until completion of the reaction (controlled by TLC). The reaction mixture was concentrated under reduced pressure. The crude residue was purified by flash chromatography on silica gel.

General procedure C: Sonogashira reaction

In a dry sealed tube, the halogenated PyTAP (1 equiv) was solubilized in a DME / water (1 / 1, 0.1M). Potassium carbonate (2.5 equiv) and the corresponding alkyne (2 equiv) were introduced. The resulted reaction mixture was degassed with argon for 10 min before the introduction of $Pd(PPh_3)_4$ (20 mol%) and CuI (20 mol%). The

reaction was stirred at 50 – 60 °C until completion of the reaction (controlled by TLC). The reaction mixture was concentrated under reduced pressure. The crude residue was purified by flash chromatography on silica gel.

2-(phenyl)pyrazolo[1',2':1,2][1,2,3]triazolo[4,5-b]pyrazin-6-ium-5-ide (2a)

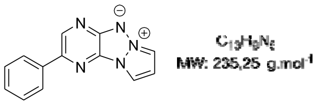

According to the general procedure A and the reaction with **1a** and phenyl boronic acid, the product was obtained in 99% yield (11 mg, 0.06 mmol) as a yellow solid.

mp = 206°C ; **^{1}H RMN** (400 MHz, $CDCl_3$) δ_H 8.95 (s, 1H), 8.13 (d, *J* = 3.3 Hz, 1H), 8.01 (d, *J* = 8.4 Hz, 2H), 7.85 (d, *J* = 2.6 Hz, 1H), 7.51 (t, *J* = 7.6 Hz, 2H), 7.41 (t, *J* = 7.4 Hz, 1H), 6.94 (t, *J* = 3.0 Hz, 1H); **^{13}C RMN** (101 MHz, CDCl3) δ_C 151.4, 149.4, 141.1, 139.4, 136.9, 129.0, 128.4, 126.1, 110.2, 109.7, 109.1; **HRAM (ESI$^+$)**: *m/z* calcd for $C_{13}H_{10}N_5^+$ [(M+H)$^+$]: 236.0931, found 236.0931.

2-(4-Methoxyphenyl)pyrazolo[1',2':1,2][1,2,3]triazolo[4,5-b]pyrazin-6-ium-5-ide (2b)

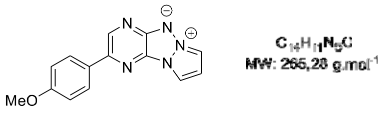

According to the general procedure A and the reaction with **1a** and 4-methoxyphenyl boronic acid, the product was obtained in 64% yield (35 mg, 0.13 mmol) as a yellow solid

mp=206°C ; ^{1}H **RMN** (400 MHz, $CDCl_3$) δ 8.89 (s, 1H), 8.09 (d, *J* = 3.3 Hz, 1H), 7.95 (d, *J* = 8.8 Hz, 2H), 7.82 (d, *J* = 2.6 Hz, 1H), 7.03 (d, *J* = 8.8 Hz, 2H), 6.92 (t, *J* = 3.0 Hz, 1H), 3.88 (s, 3H); ^{13}C **RMN** (101 MHz, $CDCl_3$) δ 160.1, 151.0, 140.6, 139.5, 129.6, 128.6, 127.4, 114.5, 109.8, 109.6, 108.7, 55.4; **HRAM (ESI$^+$)**: *m/z* calcd for $C_{14}H_{12}N_5O^+$ [(M+H)$^+$]: 266.1036, found 266.1031.

2-(4-Trilfuoromethylphenyl)pyrazolo[1',2':1,2][1,2,3]triazolo[4,5-b]pyrazin-6-ium-5-ide (2c)

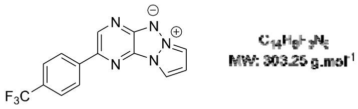

According to the general procedure A and the reaction with **1a** and 4-trifluoromethylphenyl boronic acid, the product was obtained in 70% yield (44 mg, 0.15 mmol) as a yellow solid.

^{1}H RMN (400 MHz, $CDCl_3$) δ_H 8.98 (s, 1H), 8.16 (d, *J* = 3.3 Hz, 1H), 8.14 (d, *J* = 8.3 Hz, 2H), 7.88 (d, *J* = 2.6 Hz, 1H), 7.74 (d, *J* = 8.3 Hz, 2H), 6.96 (dd, *J* = 3.3, 2.8 Hz, 1H); **^{13}C RMN (101 MHz, $CDCl_3$)** δ_C 152.0, 141.4, 140.4, 137.5, 130.2 (q, *J* = 32.1 Hz), 129.1, 126.3, 126.1 (q, *J* = 3.8 Hz), 124.3 (q, *J* = 272.4 Hz), 110.9, 110.1, 110.0; **HRAM (ESI$^+$)**: *m/z* calcd for $C_{14}H_9F_3N_5^+$ [(M+H)$^+$]: 304.0805, found 304.0801.

2-(Pyrimidin-5-yl)pyrazolo[1',2':1,2][1,2,3]triazolo[4,5-b]pyrazin-6-ium-5-ide (2d)

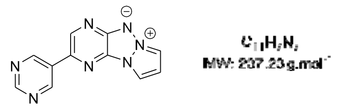

According to the <u>general procedure A</u> and the reaction with **1a** and 4-frifluoromethylphenyl boronic acid, the product was obtained in 99% yield (53 mg, 0.21 mmol) as an orange solid.

mp = 250°C (dégradation); **^{1}H RMN (400 MHz, CDCl$_3$)** δ_H 9.36 (s, 2H), 9.23 (s, 1H), 8.95 (s, 1H), 8.19 (dd, *J* = 3.4, 0.5 Hz, 1H), 7.92 (dd, *J* = 2.7, 0.5 Hz, 1H), 7.00 (dd, *J* = 3.4, 2.7 Hz, 1H); **^{13}C RMN (101 MHz, CDCl$_3$)** δ_C 157.8, 153.9, 140.7, 132.9, 130.6, 111.4, 110.4, 110.1; **HRAM (ESI$^+$)**: *m/z* calcd for $C_{11}H_8N_7{}^+$ [(M+H)$^+$]: 238.0836, found 238.0837.

2-((4-(trifluoromethyl)phenyl)ethynyl)pyrazolo[1',2':1,2][1,2,3]triazolo[4,5-b]pyrazin-6-ium-5-ide (3a)

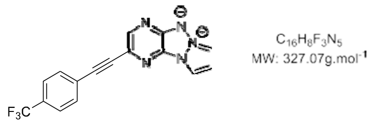

According to the <u>general procedure B</u> and the reaction with **1a** and 4-trifluoromethylphenyl acetylene, the product was obtained in 99% yield (20 mg, 0.06 mmol) as a yellow solid.

mp = 246°C ; **^{1}H RMN (400 MHz, CDCl$_3$)** δ_H 8.69 (s, 1H), 8.14 (d, *J* = 3.4 Hz, 1H), 7.89 (d, *J* = 2.6 Hz, 1H), 7.72 (d, *J* = 8.1 Hz, 2H), 7.64 (d, *J* = 8.2 Hz, 2H), 6.97 (t, *J* = 3.0 Hz, 1H);); **^{13}C RMN (101 MHz, CDCl$_3$)** δ_C 151.2, 147.5, 132.0, 130.5 (q, *J* = 32.6 Hz), 128.8, 126.3, 125.5 (q, *J* = 3.9 Hz), 123.9 (q, *J* = 270.7 Hz), 123.6, 111.6, 110.6, 110.3, 89.8, 89.2; **HRAM (ESI$^+$)**: *m/z* calcd for $C_{16}H_9F_3N_5{}^+$ [(M+H)$^+$]: 328.0804, found 328.0804.

2-((4-methoxyphenyl)ethynyl)pyrazolo[1',2':1,2][1,2,3]triazolo[4,5-b]pyrazin-6-ium-5-ide (3b)

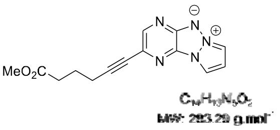

According to the <u>general procedure C</u> and the reaction with **1a** and methyl-5-hexynoate, the product was obtained in 70% yield (42 mg, 0.17 mmol) as an orange solid.

mp = 148°C ; **^{1}H RMN (400 MHz, CDCl$_3$)** δ_H 8.46 (s, 1H), 8.18 (s, 1H), 7.94 (s, 2H), 7.50 (d, *J* = 8.1 Hz, 2H), 6.91 (d, *J* = 8.1 Hz, 2H), 3.85 (s, 3H); **^{13}C RMN (101 MHz, CDCl$_3$)** δ_C 160.6, 152.6, 143.7, 133.4, 130.8, 129.2, 114.4, 114.0, 111.7, 110.5, 108.4, 93.4, 77.2, 55.5; **HRAM (ESI$^+$)**: *m/z* calcd for $C_{14}H_{14}N_5O_2{}^+$ [(M+H)$^+$]: 284.1442, found 284.1147.

8-(phenylethynyl)pyrazolo[1',2':1,2][1,2,3]triazolo[4,5-b]pyrazin-6-ium-5-ide(4a)

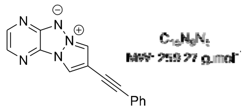

According to the <u>general procedure B</u> and the reaction with **1b** and phenyl acetylene, the product was obtained in 51% yield (70 mg, 0.27 mmol) as a yellow solid.

According to the <u>general procedure B</u> and the reaction with **4f** and phenyl iodide, the product was obtained in 95% yield (134 mg, 0.52 mmol) as a yellow solid.

mp = 238°C; **^{1}H RMN (400 MHz, CDCl$_3$)** δ_H 8.48 (d, *J* = 2.7 Hz, 1H), 8.21 (s, 1H), 7.98 (s, 1H), 7.95 (d, *J* = 2.7 Hz, 1H), 7.60 – 7.52 (m, 2H), 7.43 – 7.35 (m, 3H); **^{13}C RMN (101 MHz, CDCl$_3$)** δ_C 152.7, 143.8, 131.8, 130.9, 129.3, 128.7, 122.0, 111.9, 110.5, 107.9, 93.3, 78.1; **HRAM (ESI$^+$)**: *m/z* calcd for $C_{15}H_{10}N_5{}^+$ [(M+H)$^+$]: 260.0931, found 260.0931.

8-((4-methoxyphenyl)ethynyl)pyrazolo[1',2':1,2][1,2,3]triazolo[4,5-b]pyrazin-6-ium-5-ide (4b)

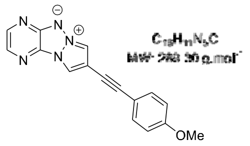

According to the <u>general procedure B</u> and the reaction with **1b** and 4-methoxyphenyl acetylene, the product was obtained in 44% yield (45 mg, 0.15 mmol) as a yellow solid.

mp = 210°C; **^{1}H RMN (400 MHz, CDCl$_3$)** δ_H 8.46 (d, *J* = 2.7 Hz, 1H), 8.18 (s, 1H), 7.94 (d, *J* = 1.6 Hz, 3H), 7.55 – 7.46 (m, 2H), 6.95 – 6.87 (m, 2H); **^{13}C RMN (101 MHz, CDCl$_3$)** δ_C 160.6, 152.6, 143.7, 133.4, 130.8, 129.2, 114.4, 114.0, 111.7, 110.5, 108.4, 93.4, 55.5; **HRAM (ESI$^+$)**: *m/z* calcd for $C_{16}H_{12}N_5O^+$ [(M+H)$^+$]: 290.1036, found 290.1039.

8-((4-(trifluoromethyl)phenyl)ethynyl)pyrazolo[1',2':1,2][1,2,3]triazolo[4,5-b]pyrazin-6-ium-5- ide (4c)

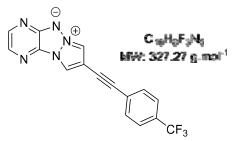

According to the <u>general procedure B</u> and the reaction with **1b** and 4-trifluoromethylphenyl acetylene, the product was obtained in 58% yield (100 mg, 0.31 mmol) as a yellow solid.

mp = 237°C; **^{1}H RMN (400 MHz, CDCl$_3$)** δ_H 8.49 (d, *J* = 2.7 Hz, 1H), 8.25 (s, 1H), 7.99 (s, 1H), 7.97 (d, *J* = 2.7 Hz, 1H), 7.66 (s, 4H); **^{13}C RMN (101 MHz, CDCl$_3$)** δ_C 152.5, 143.8, 131.9, 131.0, 131.0, 125.7, 125.5, 125.46, 111.9, 110.2, 107.0, 91.6, 80.4; **HRAM (ESI$^+$)**: *m/z* calcd for $C_{16}H_9F_3N_5^+$ [(M+H)$^+$]: 328.0805, found 328.0805.

8-((trimethylsilyl)ethynyl)pyrazolo[1',2':1,2][1,2,3]triazolo[4,5-b]pyrazin-6-ium-5-ide (4d)

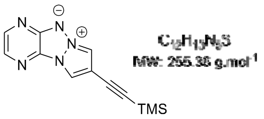

According to the <u>general procedure B</u> and the reaction with **1b** and trimethyl(ethylnyl)silane, the product was obtained in 68% yield (304 mg, 1.19 mmol) as a yellow solid.

mp = 197°C; **^{1}H RMN (400 MHz, CDCl$_3$)** δ_H 8.46 (d, *J* = 2.7 Hz, 1H), 8.14 (s, 1H), 7.94 (d, *J* = 2.6 Hz, 1H), 7.88 (s, 1H), 0.29 (s, 9H); **^{13}C RMN (101 MHz, CDCl$_3$)** δ_C 152.9, 143.8, 130.9, 112.5, 110.8, 107.8, 99.8, 95.2, 93.1, -0.2; **HRAM (ESI$^+$)**: *m/z* calcd for $C_{12}H_{14}N_5Si^+$ [(M+H)$^+$]: 256.1018, found 256.1016.

8-((triethylsilyl)ethynyl)pyrazolo[1',2':1,2][1,2,3]triazolo[4,5-b]pyrazin-6-ium-5-ide (4e)

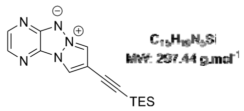

According to the <u>general procedure B</u> and the reaction with **1b** and triethyl(ethylnyl)silane, the product was obtained in 68% yield (65 mg, 0.22 mmol) as a yellow solid.

mp =103°C ; **^{1}H RMN (400 MHz, CDCl$_3$)** δ_H 8.45 (d, *J* = 2.7 Hz, 1H), 8.15 (s, 1H), 7.92 (d, *J* = 2.7 Hz, 1H), 7.88 (s, 1H), 1.06 (t, *J* = 7.8 Hz, 9H), 0.71 (q, *J* = 7.8 Hz, 6H); **^{13}C RMN (101 MHz, CDCl$_3$)** δ_C 152.8, 143.8, 130.9, 129.1, 112.6, 110.9, 107.9, 97.6, 94.2, 7.6, 4.3, 0.1; **HRAM (ESI$^+$)**: *m/z* calcd for $C_{15}H_{20}N_5Si^+$ [(M+H)$^+$]: 298.1482, found 298.1482.

8-ethynylpyrazolo[1',2':1,2][1,2,3]triazolo[4,5-b]pyrazin-6-ium-5-ide (4f)

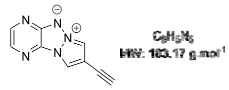

Compound **4d** (230mg, 0.90mmol, 1éq.) was solubilized in a saturated solution of ammonia in methanol (28 mL, 7N). The reaction mixture stirred for 25 min at room temperature before concentration under reduced pressure. The crude residue was was purified by flash chromatography on silica gel (elution DCM/AcOEt 7/3). The product was obtained in 89% yield (147 mg, 0.80 mmol) as a light yellow/green solid.

mp = 234°C ; **^{1}H RMN (400 MHz, CDCl$_3$)** δ_H 8.47 (d, *J* = 2.7 Hz, 1H), 8.19 (s, 1H), 7.95 (d, *J* = 2.7 Hz, 1H), 7.92 (s, 1H), 3.23 (d, *J* = 2.6 Hz, 1H); **^{13}C RMN (101 MHz, CDCl$_3$)** δ_C 152.6, 143.8, 130.9, 128.9, 112.6, 110.7, 106.4, 81.6, 72.6; **HRAM (ESI$^+$)**: *m/z* calcd for $C_9H_6N_5^+$ [(M+H)$^+$]:184.0618, found 184.0615.

8-(3-((tert-butoxycarbonyl)amino)prop-1-yn-1-yl)pyrazolo[1',2':1,2][1,2,3]triazolo[4,5-b]pyrazin-6-ium-5-ide (4g)

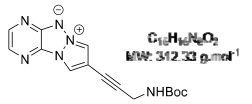

According to the <u>general procedure C</u> and the reaction with **1c** and *tert*-butyl-prop-2-ynylcarbamate, the product was obtained in 75% yield (196 mg, 0.63 mmol) as a yellow solid.

mp = 185°C (degradation); **^{1}H RMN (400 MHz, CDCl$_3$)** δ_H 8.47 (s, 1H), 8.11 (s, 1H), 7.95 (s, 1H, H1 ou H2), 7.86 (s, 1H), 4.81 (bs, 1H), 4.19 (d, *J* = 5.1 Hz, 2H), 1.48 (s, 9H); **^{13}C RMN (101 MHz, CDCl$_3$)** δ_C 155.4, 152.8, 143.8, 132.3, 130.9, 128.7, 112.2, 110.7, 107.2, 90.1, 80.3, 72.1, 28.5; **HRAM (ESI$^+$)**: *m/z* calcd for $C_{15}H_{17}N_6O_2{}^+$ [(M+H)$^+$]: 313.1407, found 313.1408.

8-(3-aminoprop-1-yn-1-yl)pyrazolo[1',2':1,2][1,2,3]triazolo[4,5-b]pyrazin-6-ium-5-ide (4h)

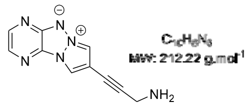

In a round bottom flask, **4g** (45 mg, 0.144 mmol) was solubilized in DCM (2 mL). TFA (0.11 mL, 1.44 mmol, 10 eq.) was added and the mixture was stirred at room temperature for 6h. Water was added and aqueous phase was extracted and washed three times with DCM. Saturated K_2CO_3 aqueous solution was added until pH around 9 and organic phase was extracted with DCM. Solvent was evaporated under vaccum to give pure **4h** (28 mg, 90%) as a yellow solid.

mp =141 °C; **^{1}H RMN (400 MHz, DMSO-*d*$_6$)** δ_H 8.89 (s, 1H), 8.62 (s, 1H), 8.40 (d, *J* = 2.7 Hz, 1H), 7.93 (d, *J* = 2.6 Hz, 1H), 3.56 (s, 3H), 3.39 – 3.19 (m, 2H).;**^{13}C RMN (101 MHz, DMSO-*d*$_6$)** δ_C 152.1, 142.7, 129.6, 128.9, 113.9, 111.3, 106.6, 95.1, 71.3, 31.3; **HRAM (ESI$^+$)**: *m/z* calcd for $C_{10}H_9N_6$+ [(M+H)$^+$]: 213.0889, found 213.0886.

8-((4-methoxyphenyl)ethynyl)pyrazolo[1',2':1,2][1,2,3]triazolo[4,5-b]pyrazin-6-ium-5-ide (4i)

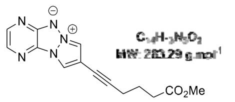

According to the <u>general procedure C</u> and the reaction with **1c** and methyl-5-hexynoate, the product was obtained in 85% yield (200 mg, 0.70 mmol) as a yellow solid.

mp = 99°C; **^{1}H RMN (400 MHz, CDCl$_3$)** δ_H 8.35 (d, *J* = 2.7 Hz, 1H), 8.03 (s, 1H), 7.83 (d, *J* = 2.7 Hz, 1H), 7.78 (s, 1H), 3.63 (s, 3H), 2.45 (td, *J* = 7.2, 5.4 Hz, 4H), 1.89 (p, *J* = 7.2 Hz, 2H); **^{13}C RMN (101 MHz, CDCl$_3$)** δ_C 173.3., 152.4, 143.3, 130.4, 128.9, 111.9, 110.5, 107.8, 93.3, 70.2, 51.7, 32.8, 23.5, 18.9; **HRAM (ESI$^+$)**: *m/z* calcd for $C_{14}H_{14}N_5O_2{}^+$ [(M+H)$^+$]: 284.114201, found 284.114140.

8-(5-carboxypent-1-yn-1-yl)pyrazolo[1',2':1,2][1,2,3]triazolo[4,5-b]pyrazin-6-ium-5-ide (4j)

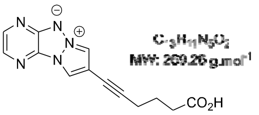

Compound **4i** (200mg, 0.70 mmol) was solubilized in methanol (4 mL) and a 1N solution of NaOH (3 mL) was added and the reaction mixture was stirred at room temperature for 2 hours. Then the reaction mixture was concentrated under reduced pressure and the crude residue was dissolved in a DCM/water mixture (1/). The aqueous phase was extracted with DCM (3 x 20 mL) then was carefully acidified to pH = 4-5 with a solution of HCl (2N) and extracted with DCM.The resulted organic phase was concentrated under reduced pressure, the product was obtained in 94% yield (178 mg, 0.66 mmol) as a yellow solid.

mp = 175°C (deg); **^{1}H RMN (400 MHz, DMSO-*d*$_6$)** δ_H 12.14 (s, 1H), 8.85 (s, 1H), 8.58 (s, 1H), 8.36 (d, *J* = 2.7 Hz, 1H), 7.90 (d, *J* = 2.7 Hz, 1H), 2.55 – 2.46 (m, 2H), 2.38 (t, *J* = 7.3 Hz, 2H), 1.78 (p, *J* = 7.2 Hz, 2H); **^{13}C RMN (101 MHz,**

DMSO) δ_C 174.0, 152.1, 142.7, 129.6, 128.9, 113.9, 111.4, 106.7, 93.0, 70.8, 32.6, 23.5, 18.2; **HRAM (ESI$^+$)**: *m/z* calcd for $C_{13}H_{12}N_5O_2^+$ [(M+H)$^+$]: 270.098551, found 270.098546.

8-(1-(4-methoxy-4-oxobutyl)-1H-1,2,3-triazol-4-yl)pyrazolo[1',2':1,2][1,2,3]triazolo[4,5-b]pyrazin-6- ium-5-ide (5)

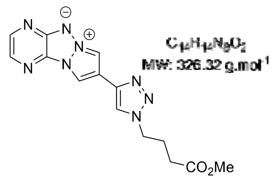

In a *t*BuOH/water (1.4mL/0.4mL) solution, compound **4f** (20 mg, 0.11 mmol), sodium ascorbate (8.4 mg, 0.04 mmol), copper sulfate (7.3 mg, 0.04 mmol) were introduced with methyl-4-azidobutanoate (19 mg, 0.13 mmol). The resulted suspension was stirred at room temperature for 24 hours and then at 50 °C for 5 hours. The reaction mixture was next concentrated under reduced pressure. The crude residue was purified by flash chromatography on silica gel. The expected product was obtained in 86% yield (31 mg, 0.09 mmol) as a yellow solid

mp = 229°C; **^{1}H RMN (400 MHz, DMSO-*d*$_6$)** δ_H 8.99 (s, 1H), 8.79 (s, 1H), 8.55 (s, 1H), 8.41 (d, *J* = 2.7 Hz, 1H), 7.95 (d, *J* = 2.7 Hz, 1H), 4.50 (t, *J* = 7.0 Hz, 2H), 3.60 (s, 3H), 2.40 (t, *J* = 7.3 Hz, 2H), 2.14 (p, *J* = 7.2 Hz, 2H); **^{13}C RMN (101 MHz, DMSO-*d*$_6$)** δ_C 172.5, 151.8, 142.6, 138.3, 129.5, 129.2, 122.3, 116.7, 107.9, 107.3, 51.4, 48.8, 30.1, 25.1; **HRAM (ESI$^+$)**: *m/z* calcd for $C_{14}H_{15}N_8O_2^+$ [(M+H)$^+$]: 327.1312, found 327.1315.

Cell Imaging

Epifluorescence Microscopy: Cellular Distribution

The HeLa (Human Cervical Carcinoma) cell line obtained from ATCC (Molsheim, France) was cultured in Dulbecco's modified Eagle's medium (DMEM) supplemented with 10% of heat-inactivated fetal bovine serum (FBS), 1% of 100× non-essential amino acid solution, 1% of L-glutamine (GlutaMAX) and 1% of streptomycin/penycilin antibiotics. Cells were seeded in a 8-well Lab Tek Chamber coverglass (Nunc, Dutsher S.A., Brumath, France) at a density of $6{\cdot}10^4$ cells/well and cultured at 37°C in a 5% humidified CO_2 atmosphere. After 24h, the cell culture medium was removed: Cells were then washed twice with Opti-MEM medium (room temperature) and incubated with pyTAP fluorescent probes during 1h and 30 min at 30 µM concentration for **3a** or 210 µM concentration for **4i** during 30 min with a 50 nM LysoTracker Green DND-26. Prior the epifluorescence imaging experiments, cells were washed twice with Opti-MEM (room temperature) in order to remove any non-specifically bound fluorescent probes. It should be noted that solutions of fluorescent probes were prepared in a DMSO and percentage of DMSO during epifluorescence microscopy was kept at 1%. Cells were observed with a Zeiss Axio Observer Z1 fluorescence inverted microscope (Zeiss, Le Pecq, France) equipped with a CCD camera (Orca-R2 Hamamatsu) with the help of the acquisition software Axiovision (Zeiss). The light source, Zeiss HXP 120 or Colibri, was combined with the following filter cubes: (i) 417 nm band pass 60 nm filter for the excitation and 536 nm band pass 40 nm filter for the emission; (ii) 485 nm band pass 20 nm filter for the excitation and 525 nm band pass 50 nm filter for the emission.

Cytotoxicity tests

Cytotoxicity tests were performed with the alamarBlue® assay (Invitrogen, France). Cells were seeded in 96-well plates at the density of $1{\cdot}10^4$ cells per well and cultured at 37°C in a 5% humidified CO_2 atmosphere. After 24h of attachment, cells were incubated with different concentrations of pyTAP fluorescent probes (i.e. 25 nM, 75 nM, 100 nM, 30 µM, 60 µM, 95 µM, 130 µM, 150 µM and 170 µM) during 24h followed by the incubation with the alamarBlue® (10% v/v) during 3-4 h at 37°C in a 5% humidified CO_2 atmosphere. Solutions of fluorescent probes were initially prepared in DMSO and the percentage of DMSO during cytotoxicity tests was maintained at 1%. The fluorescence of alamarBlue® was measured with a plate reader (Victor 3V, Perkin Elmer, France) using a 530 nm excitation wavelength and collecting the emission at 590 nm. Control cells were prepared under the same experimental conditions but without the addition of the fluorescent probes but with 3% of DMSO. For each fluorescent probe three individual experiments were performed as triplicates. An average value out of these three experiments was calculated as a mean fluorescence value and corrected for the control cells. Data were presented as the mean ± RSD.

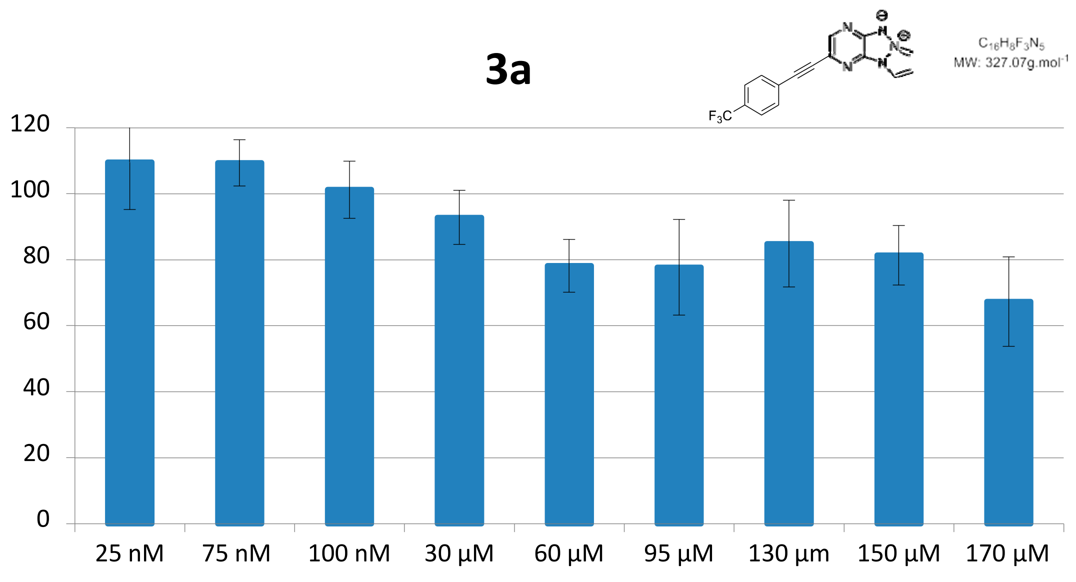

Figure S1 : Results of the cytotoxicity test performed with the Alamar blue assay for the **3a** incubated with HeLa cells during 24 h.

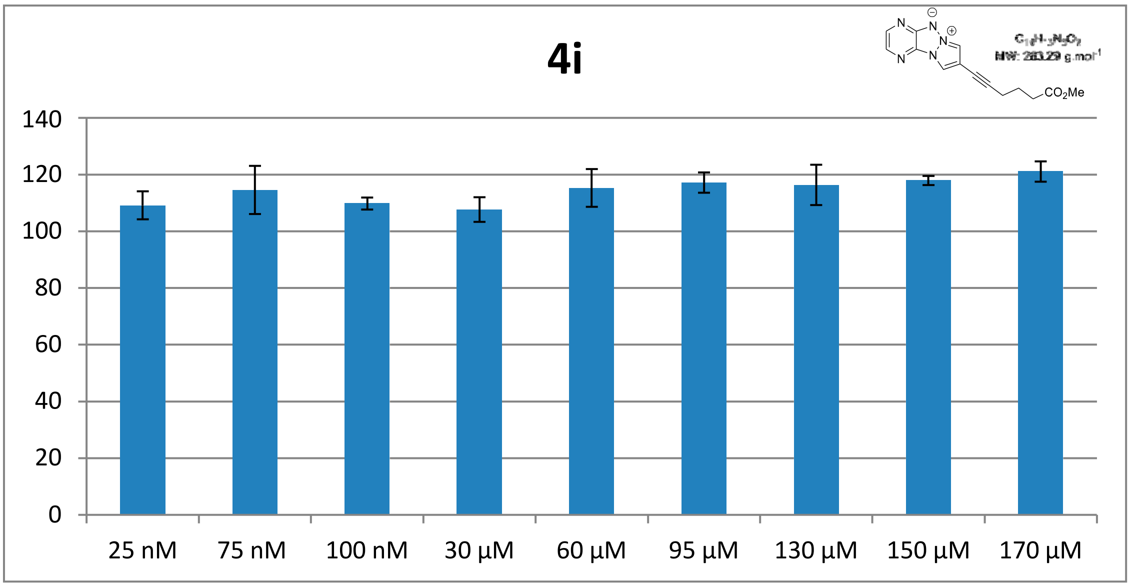

Figure S2 : Results of the cytotoxicity test performed with the Alamar blue assay for the **4i** incubated with HeLa cells during 24 h.

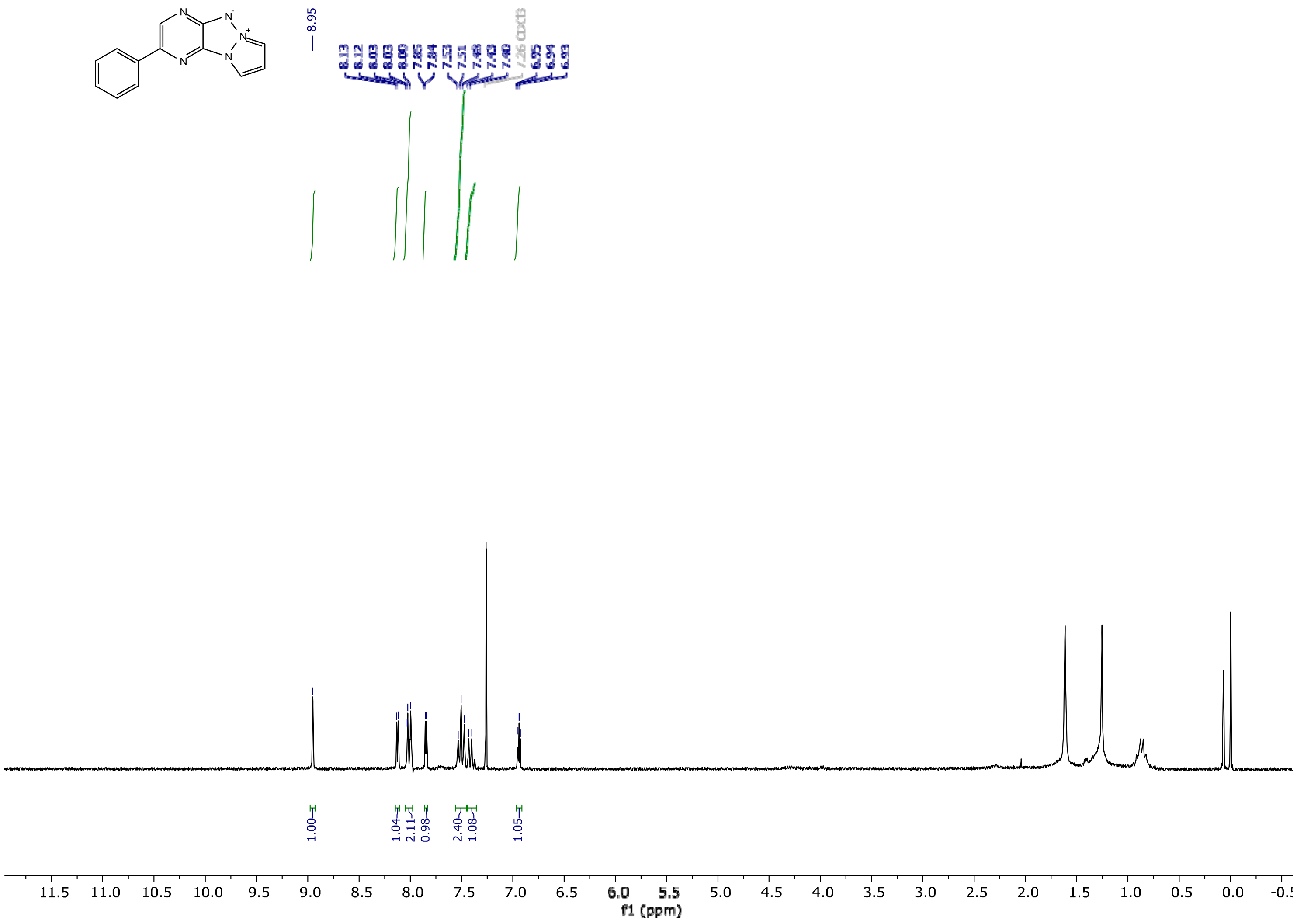
8.95
8.13
8.12
8.03
8.03
8.00
7.85
7.84
7.53
7.51
7.48
7.43
7.40
7.26 cdcl3
6.95
6.94
6.93
1.00
1.04
2.11
0.98
2.40
1.08
1.05
11.5 11.0 10.5 10.0 9.5 9.0 8.5 8.0 7.5 7.0 6.5 6.0 5.5 5.0 4.5 4.0 3.5 3.0 2.5 2.0 1.5 1.0 0.5 0.0
f1 (ppm)

^{13}C NMR (101 MHz, $CDCl_3$) spectrum 2-(phenyl)pyrazolo[1',2':1,2][1,2,3]triazolo[4,5-*b*]pyrazin-6-ium-5-ide **2a**

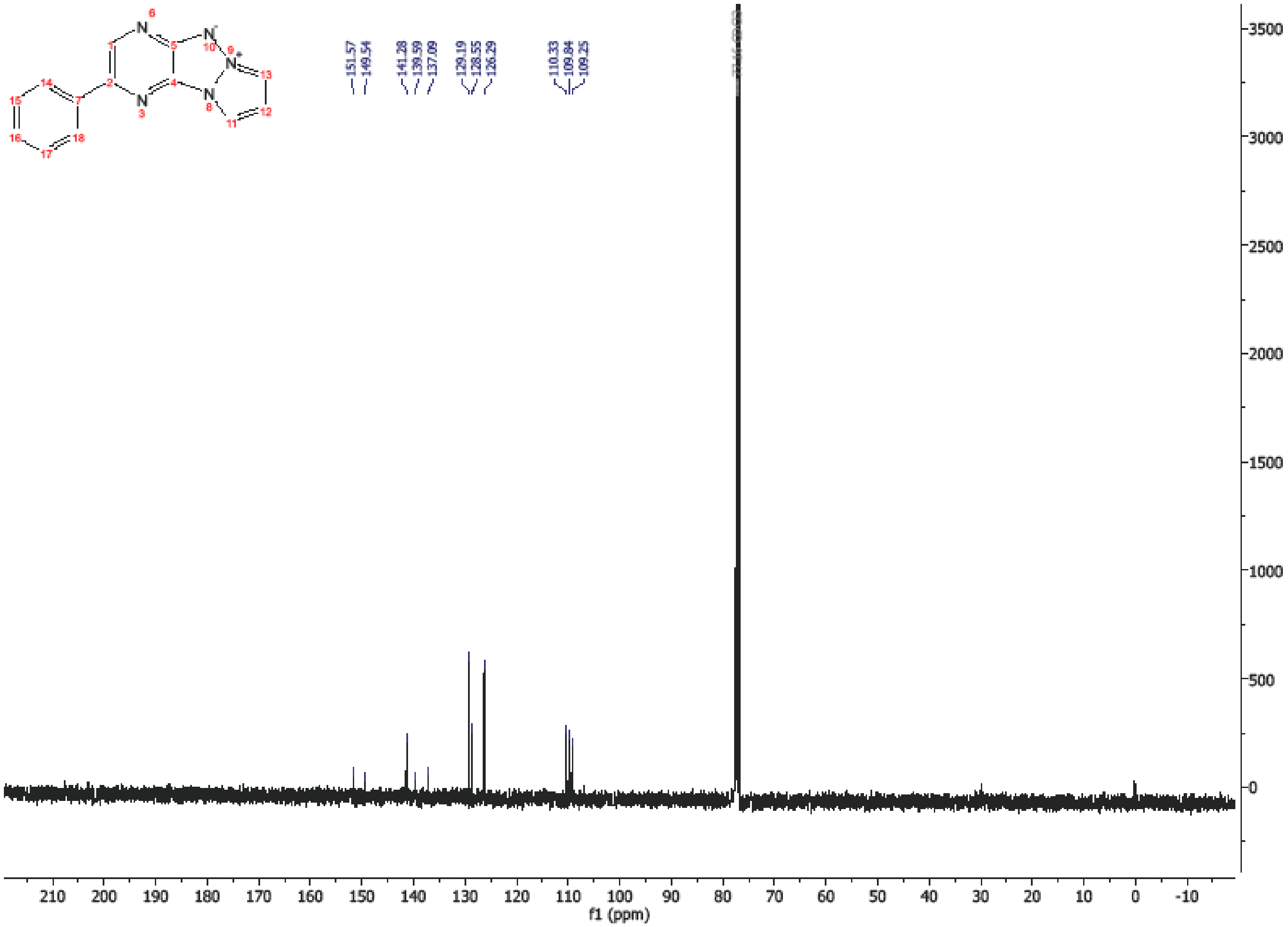

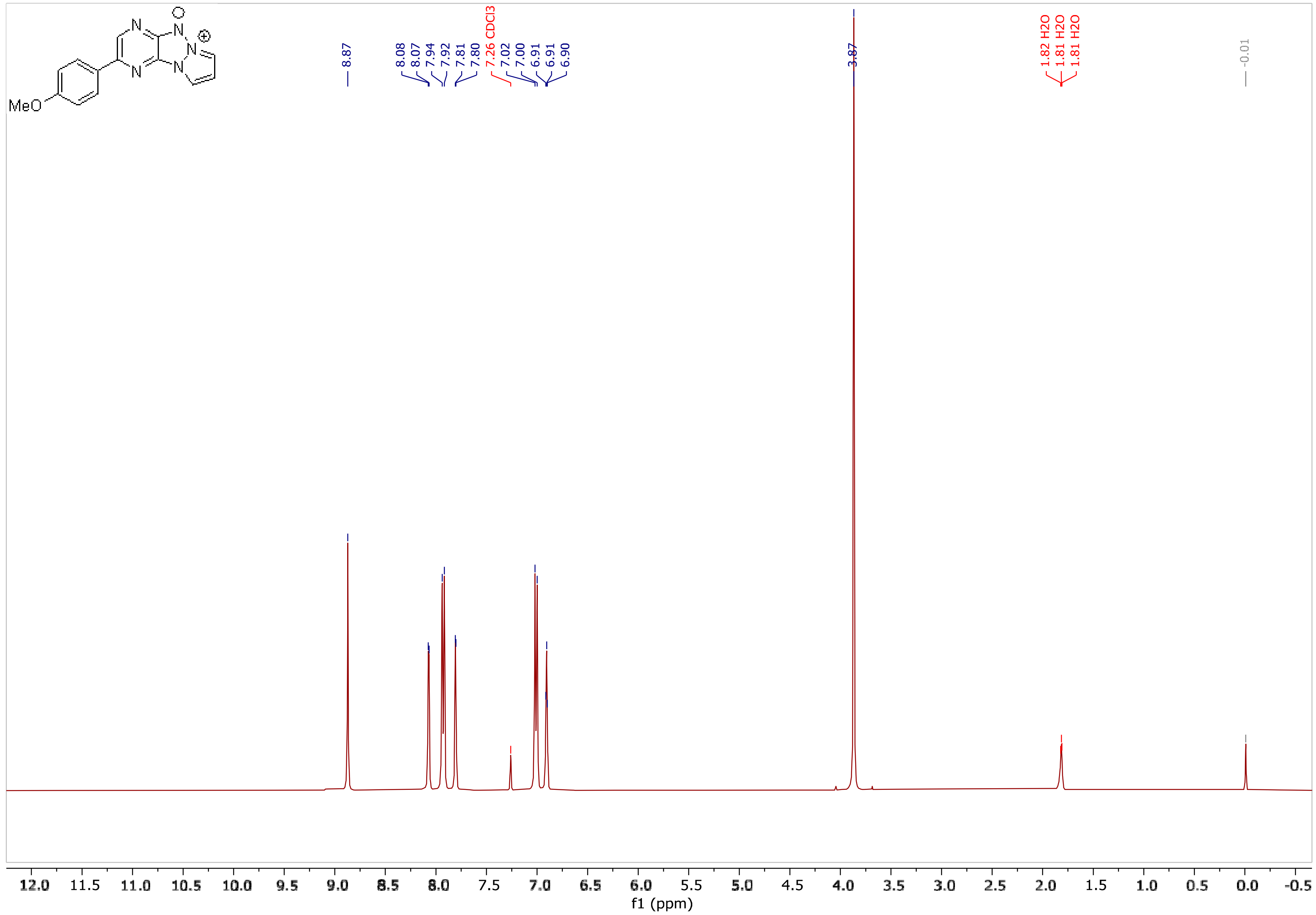
MeO
8.87
8.08
8.07
7.94
7.92
7.81
7.80
7.26 CDCl3
7.02
7.00
6.91
6.91
6.90
3.87
1.82 H2O
1.81 H2O
1.81 H2O
-0.01
12.0 11.5 11.0 10.5 10.0 9.5 9.0 8.5 8.0 7.5 7.0 6.5 6.0 5.5 5.0 4.5 4.0 3.5 3.0 2.5 2.0 1.5 1.0 0.5 0.0 -0.5
f1 (ppm)

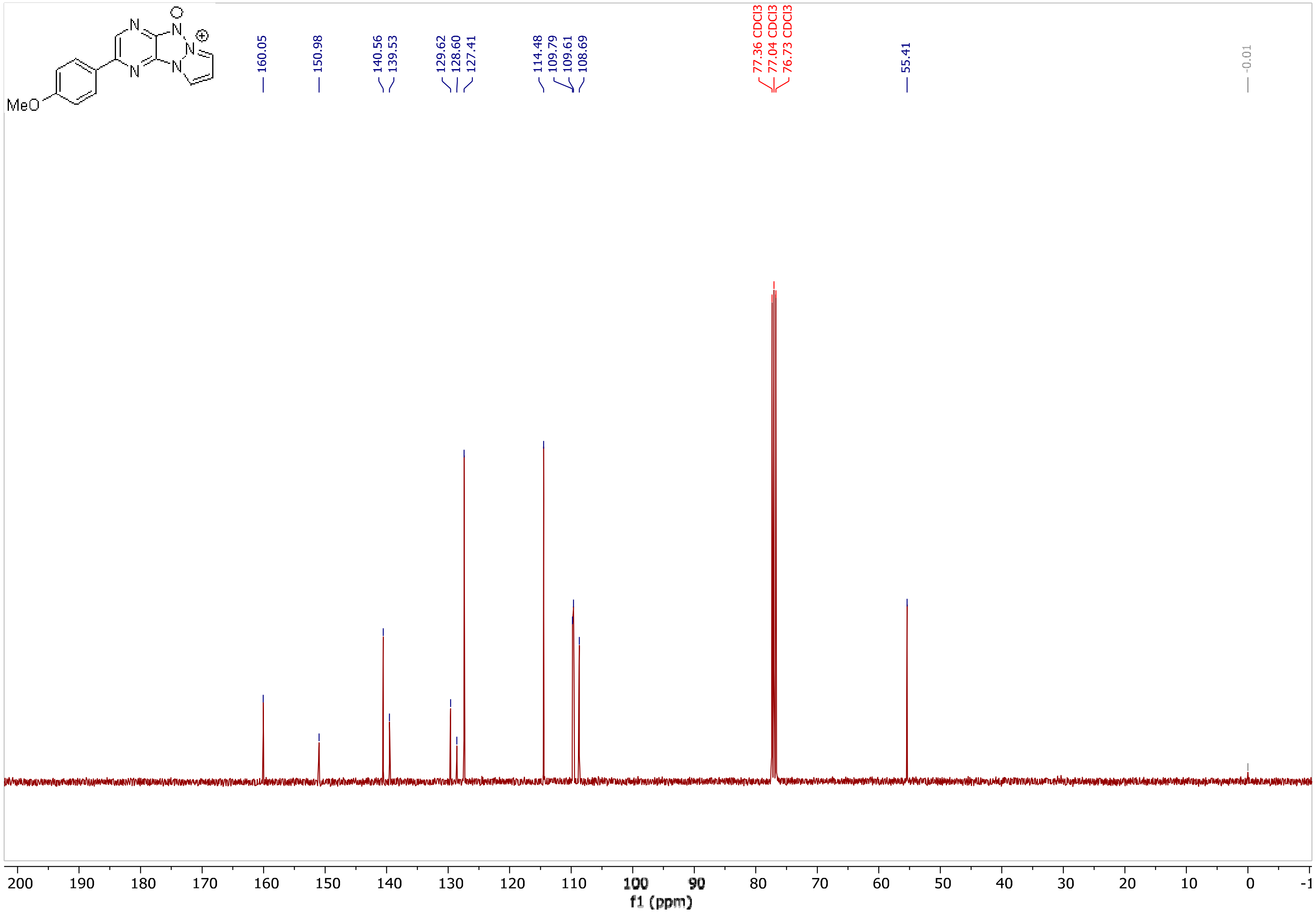
MeO
160.05
150.98
140.56
139.53
129.62
128.60
127.41
114.48
109.79
109.61
108.69
77.36 CDCl3
77.04 CDCl3
76.73 CDCl3
55.41
-0.01
200 190 180 170 160 150 140 130 120 110 100 90 80 70 60 50 40 30 20 10 0 -1
f1 (ppm)

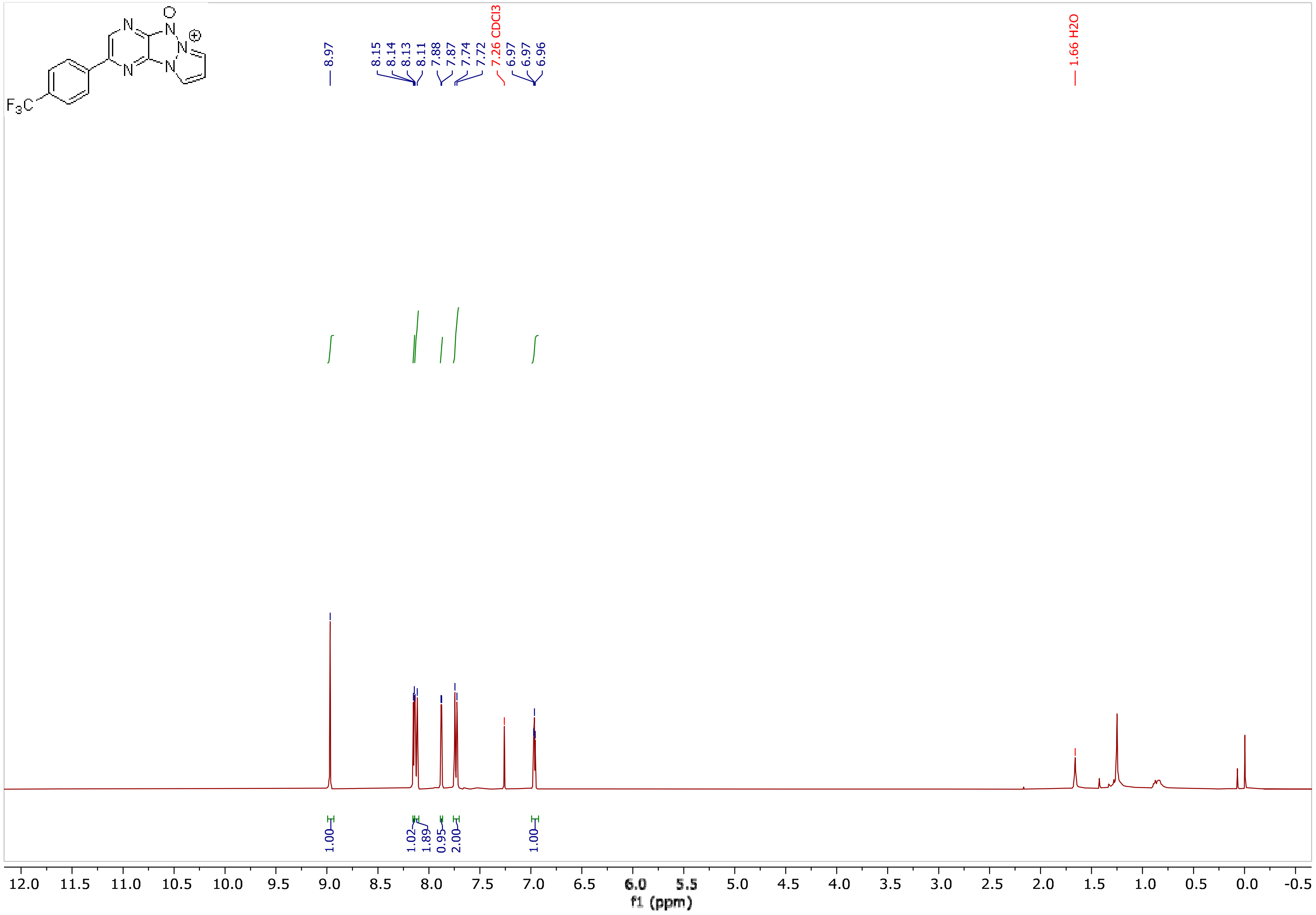
F3C
8.97
8.15
8.14
8.13
8.11
7.88
7.87
7.74
7.72
7.26 CDCl3
6.97
6.97
6.96
1.66 H2O
1.00
1.02
1.89
0.95
2.00
1.00
f1 (ppm)

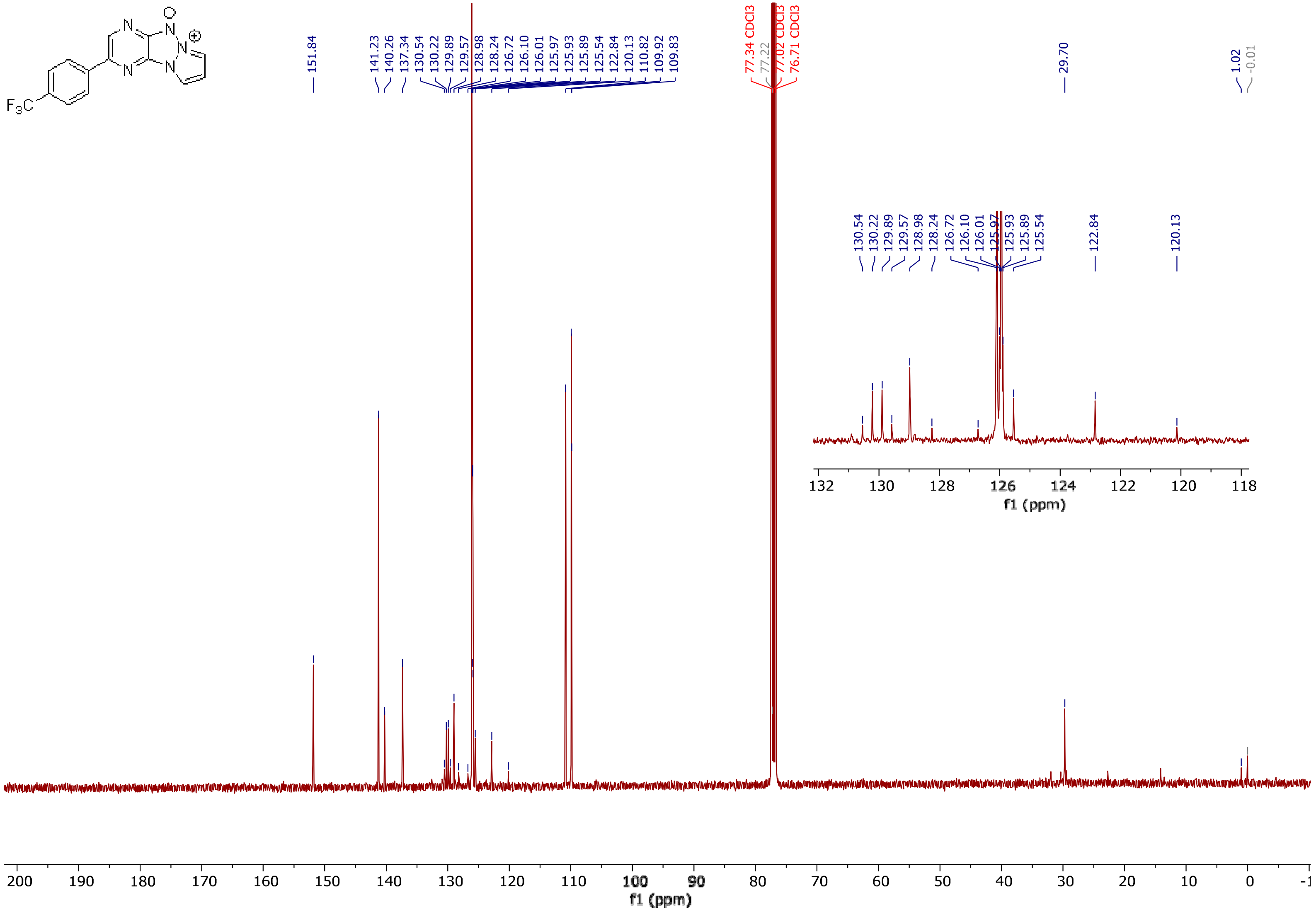

F3C
151.84
141.23
140.26
137.34
130.54
130.22
129.89
129.57
128.98
128.24
126.72
126.10
126.01
125.97
125.93
125.89
125.54
122.84
120.13
110.82
109.92
109.83
77.34 CDCl3
77.22
77.02 CDCl3
76.71 CDCl3
29.70
1.02
-0.01
f1 (ppm)
f1 (ppm)

^{19}F NMR (235 MHz, $CDCl_3$) spectrum of spectrum of 2-(4-Trilfuoromethylphenyl)pyrazolo[1',2':1,2][1,2,3]triazolo[4,5-*b*]pyrazin-6-ium-5-ide **2c**

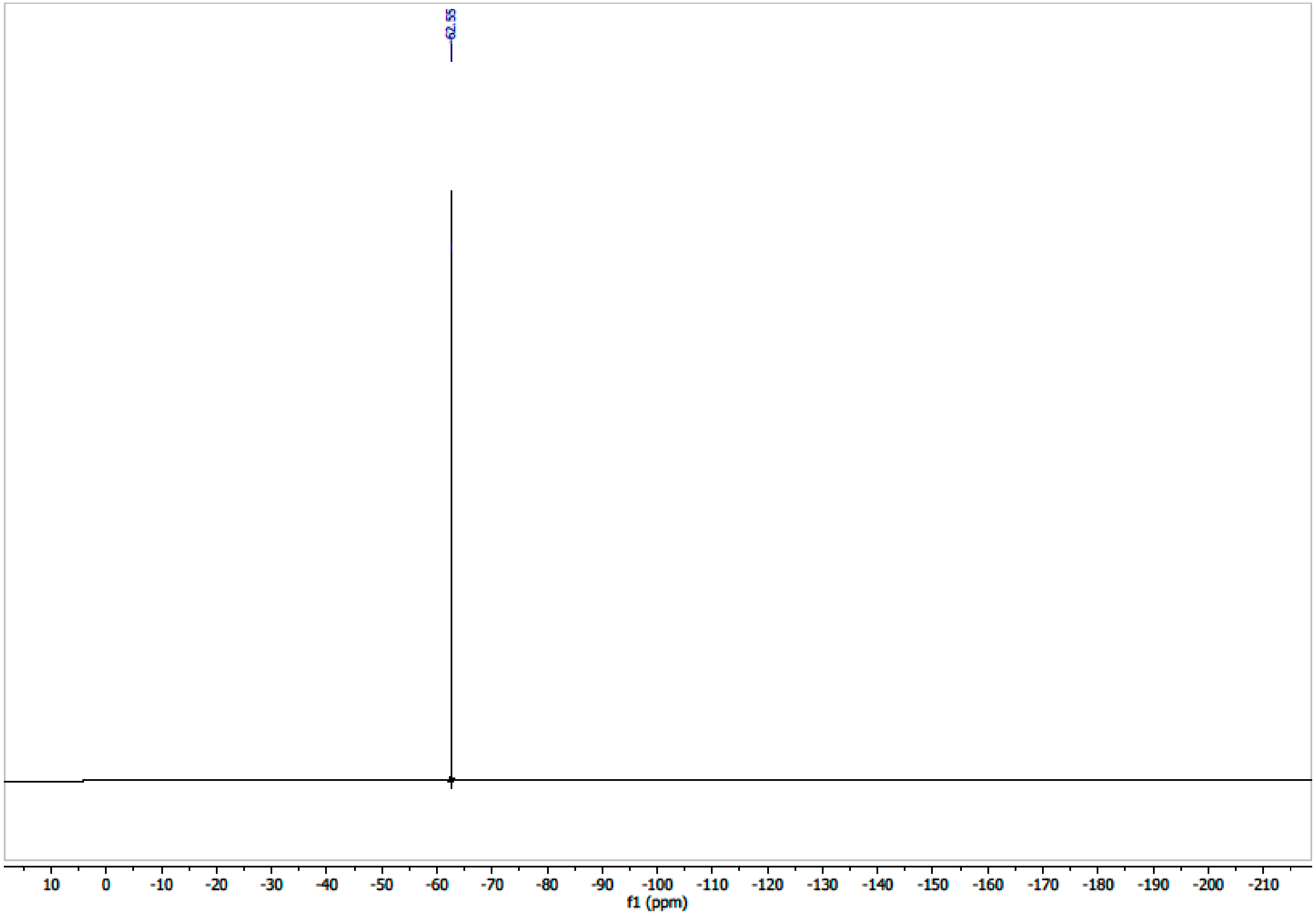

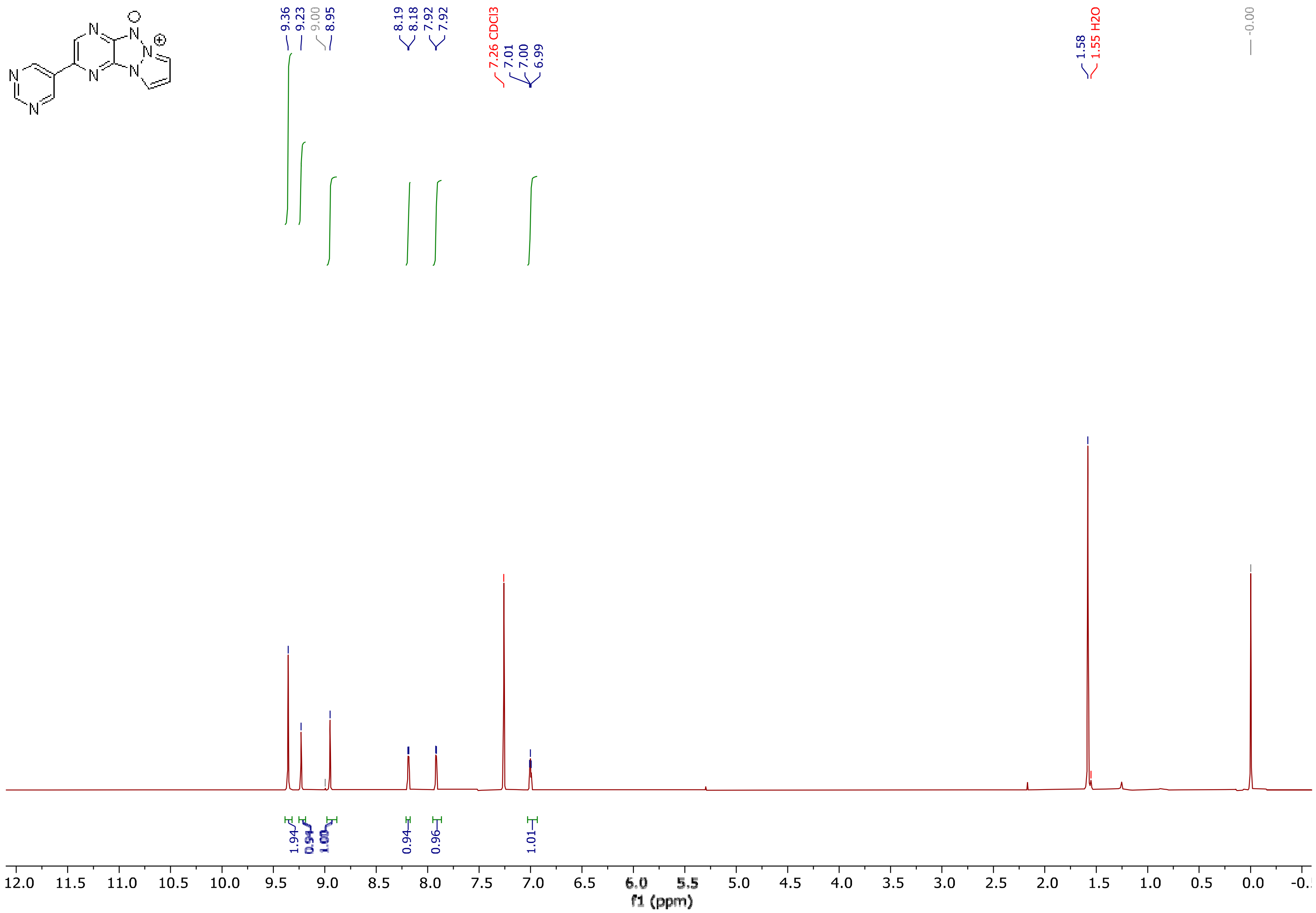

9.36
9.23
9.00
8.95
8.19
8.18
7.92
7.92
7.26 CDCl3
7.01
7.00
6.99
1.58
1.55 H2O
-0.00
1.94
0.94
0.96
1.01
12.0 11.5 11.0 10.5 10.0 9.5 9.0 8.5 8.0 7.5 7.0 6.5 6.0 5.5 5.0 4.5 4.0 3.5 3.0 2.5 2.0 1.5 1.0 0.5 0.0
f1 (ppm)

^{13}C NMR (101 MHz, $CDCl_3$) spectrum of 2-(Pyrimidin-5-yl)pyrazolo[1',2':1,2][1,2,3]triazolo[4,5-*b*]pyrazin-6-ium-5-ide **2d**

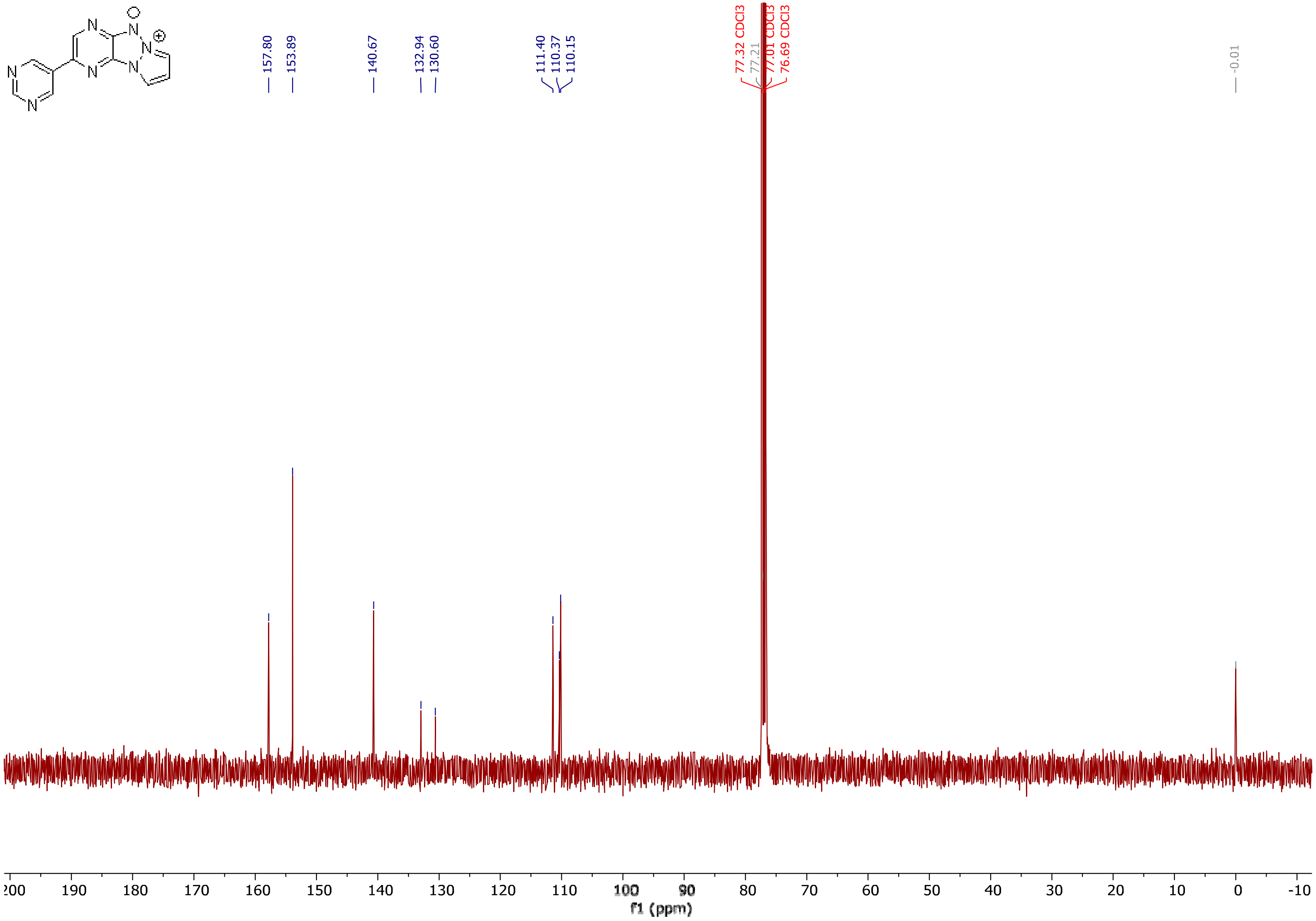

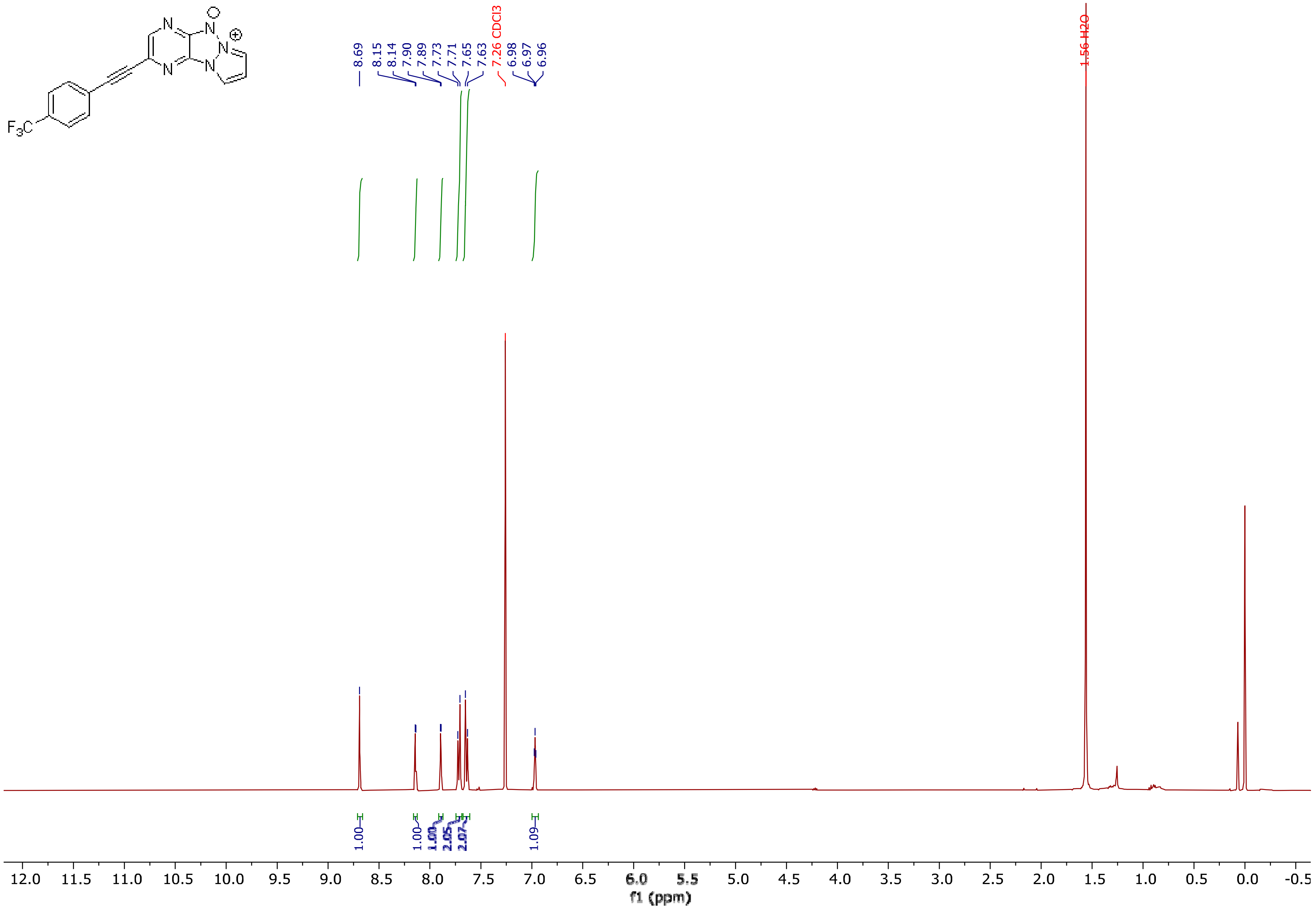

F3C
N
N
N
N
N
8.69
8.15
8.14
7.90
7.89
7.73
7.71
7.65
7.63
7.26 CDCl3
6.98
6.97
6.96
1.56 H2O
1.00
1.00
1.00
2.05
2.07
1.09
12.0 11.5 11.0 10.5 10.0 9.5 9.0 8.5 8.0 7.5 7.0 6.5 6.0 5.5 5.0 4.5 4.0 3.5 3.0 2.5 2.0 1.5 1.0 0.5 0.0 -0.5
f1 (ppm)

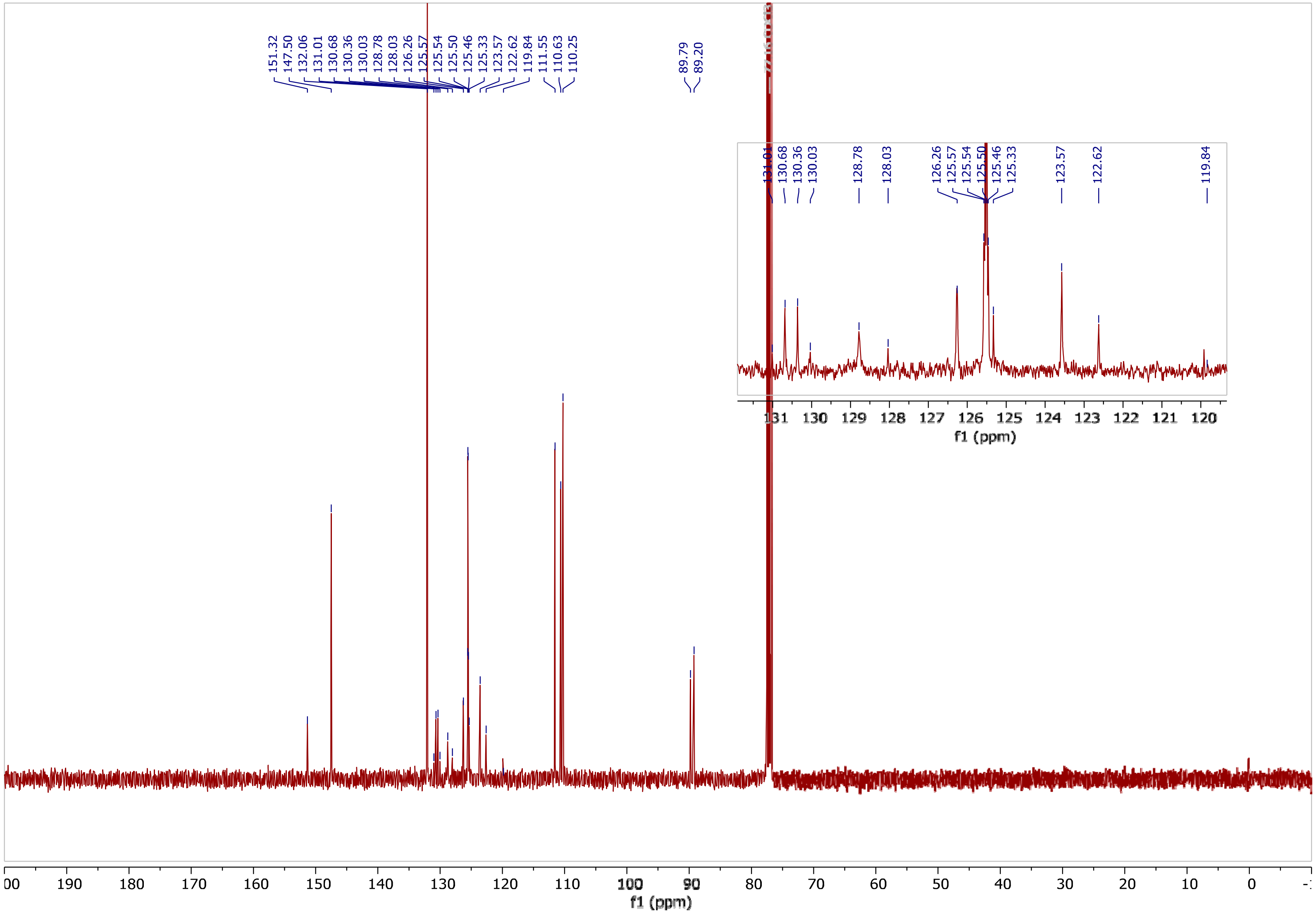
151.32
147.50
132.06
131.01
130.68
130.36
130.03
128.78
128.03
126.26
125.57
125.54
125.50
125.46
125.33
123.57
122.62
119.84
111.55
110.63
110.25
89.79
89.20
f1 (ppm)
f1 (ppm)

^{19}F NMR (235 MHz, $CDCl_3$) spectrum of 2-((4-(trifluoromethyl)phenyl)ethynyl)pyrazolo[1',2':1,2][1,2,3]triazolo[4,5-*b*]pyrazin-6-ium-5-ide **3a**

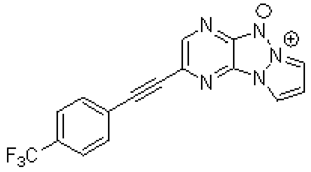

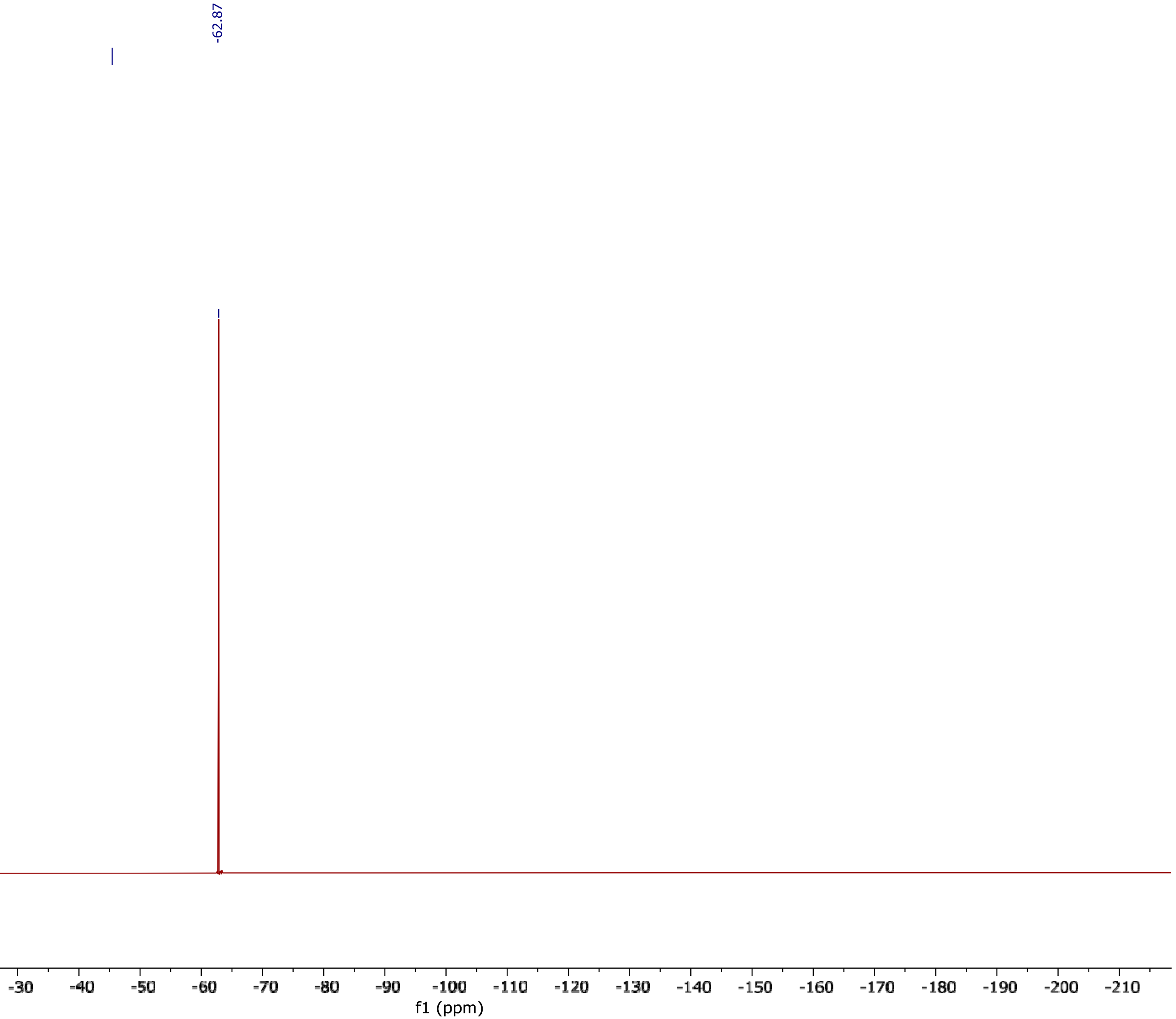

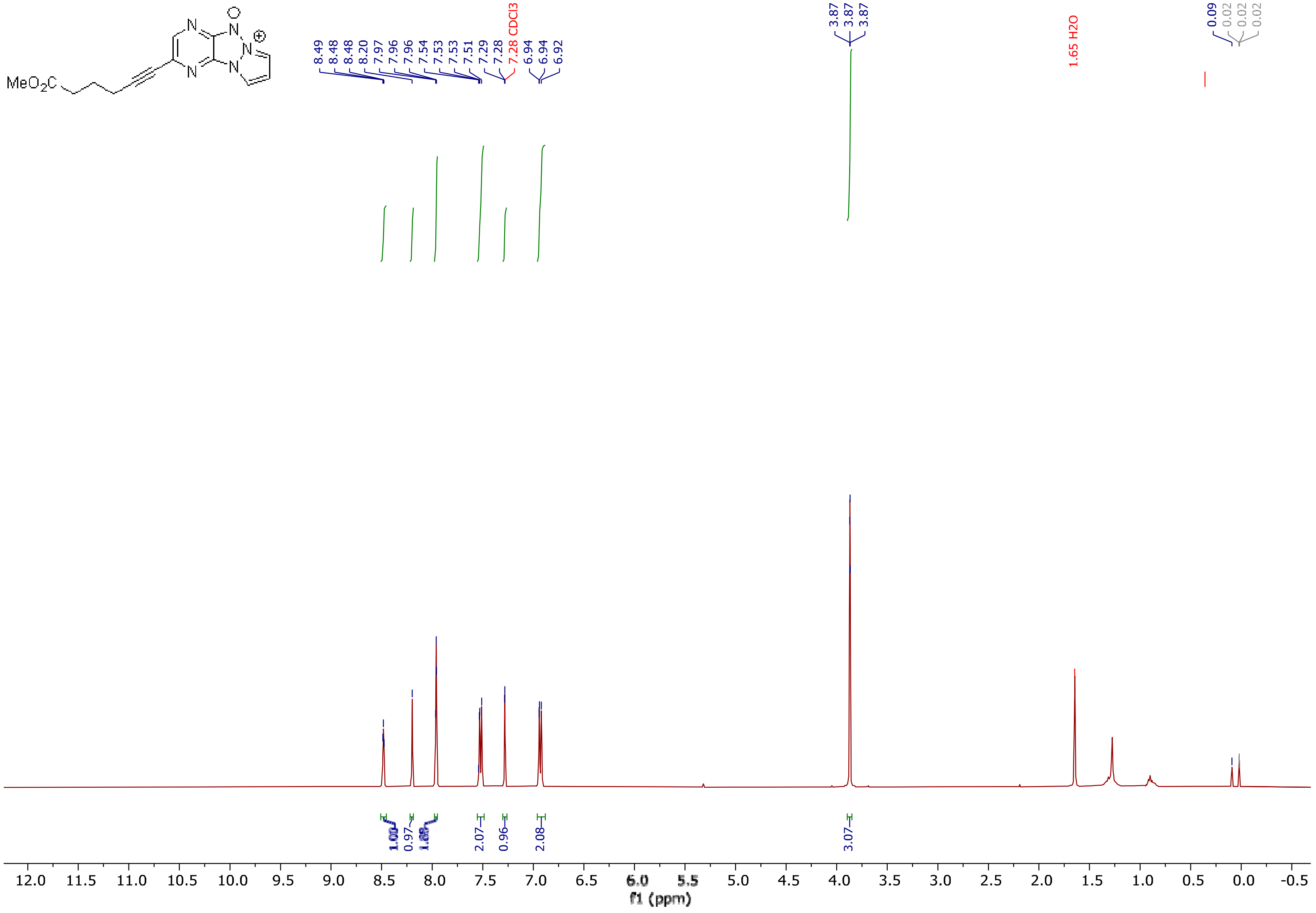

MeO2C
8.49
8.48
8.48
8.20
7.97
7.96
7.96
7.54
7.53
7.53
7.51
7.29
7.28
7.28 CDCl3
6.94
6.94
6.92
3.87
3.87
3.87
1.65 H2O
0.09
0.02
0.02
0.02
1.00
0.97
1.88
2.07
0.96
2.08
3.07
f1 (ppm)

^{13}C NMR (101 MHz, $CDCl_3$) spectrum of 2-((4-methoxyphenyl)ethynyl)pyrazolo[1',2':1,2][1,2,3]triazolo[4,5-*b*]pyrazin-6-ium-5-ide **3b**

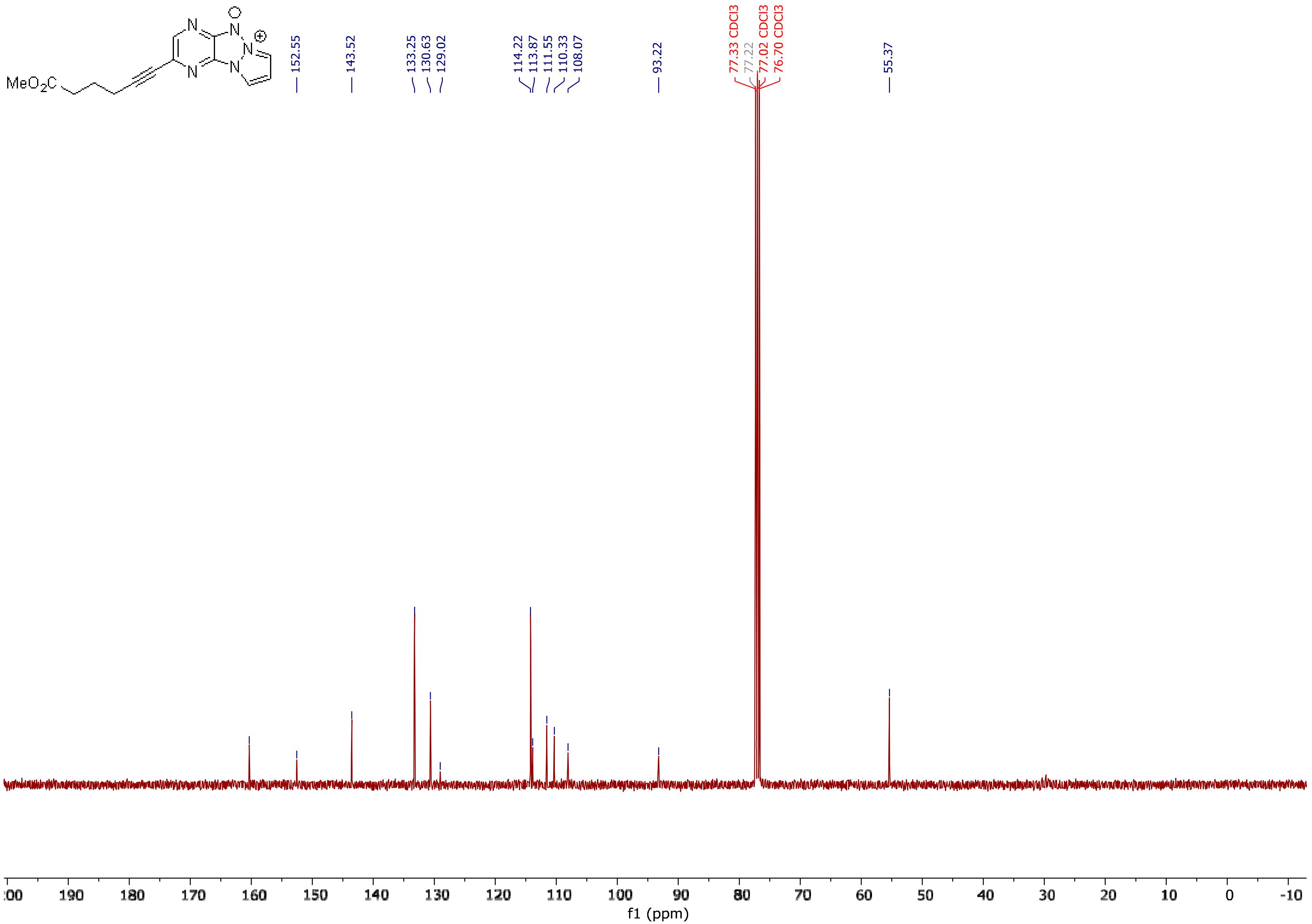

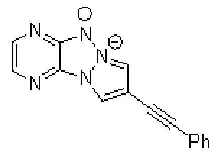
N
N
N
N
N
Ph

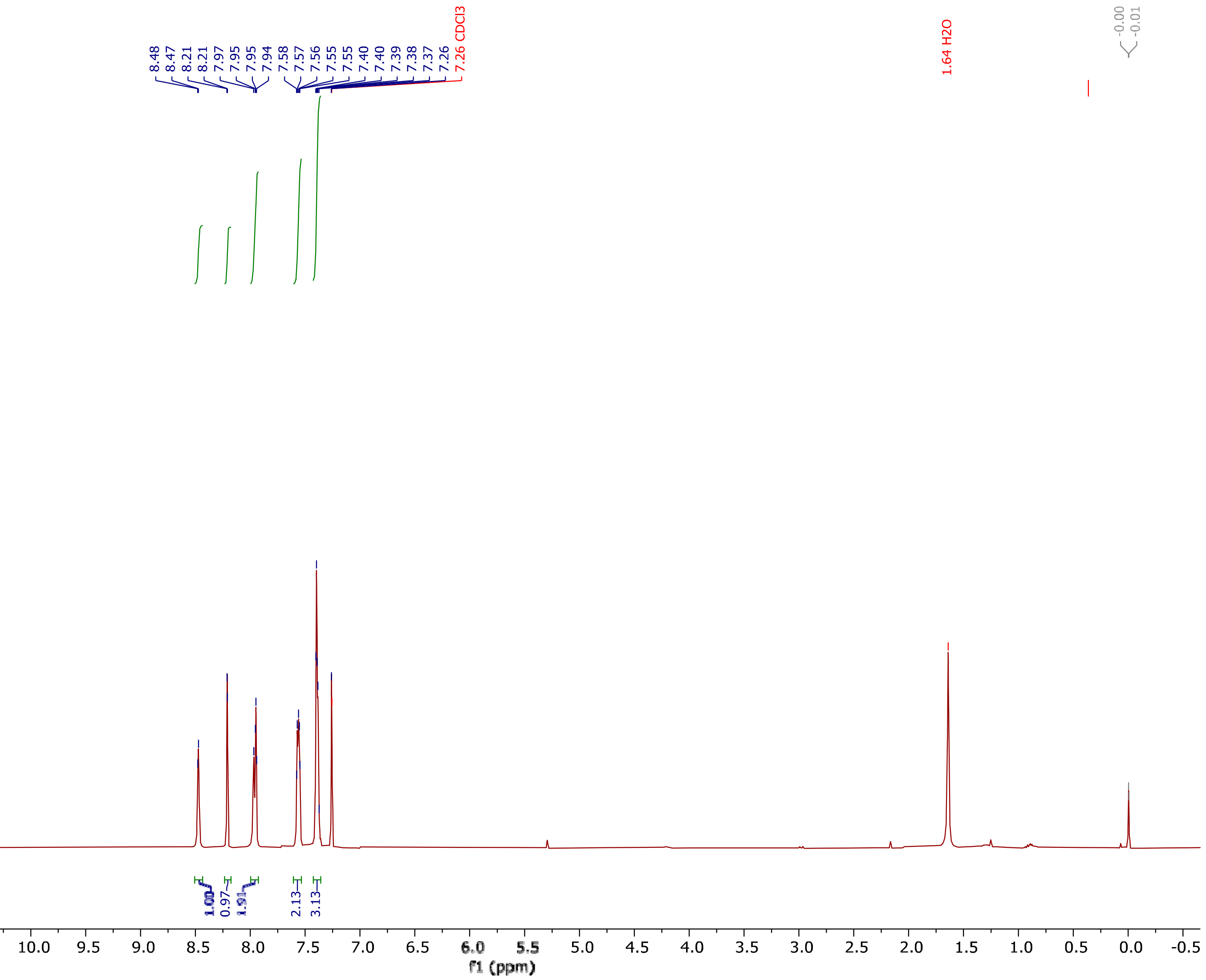
8.48
8.47
8.21
8.21
7.97
7.95
7.95
7.94
7.58
7.57
7.56
7.55
7.55
7.40
7.40
7.39
7.38
7.37
7.26
7.26 CDCl3
1.64 H2O
-0.00
-0.01
1.00
0.97
1.91
2.13
3.13
12.0
11.5
11.0
10.5
10.0
9.5
9.0
8.5
8.0
7.5
7.0
6.5
6.0
5.5
5.0
4.5
4.0
3.5
3.0
2.5
2.0
1.5
1.0
0.5
0.0
-0.5
f1 (ppm)

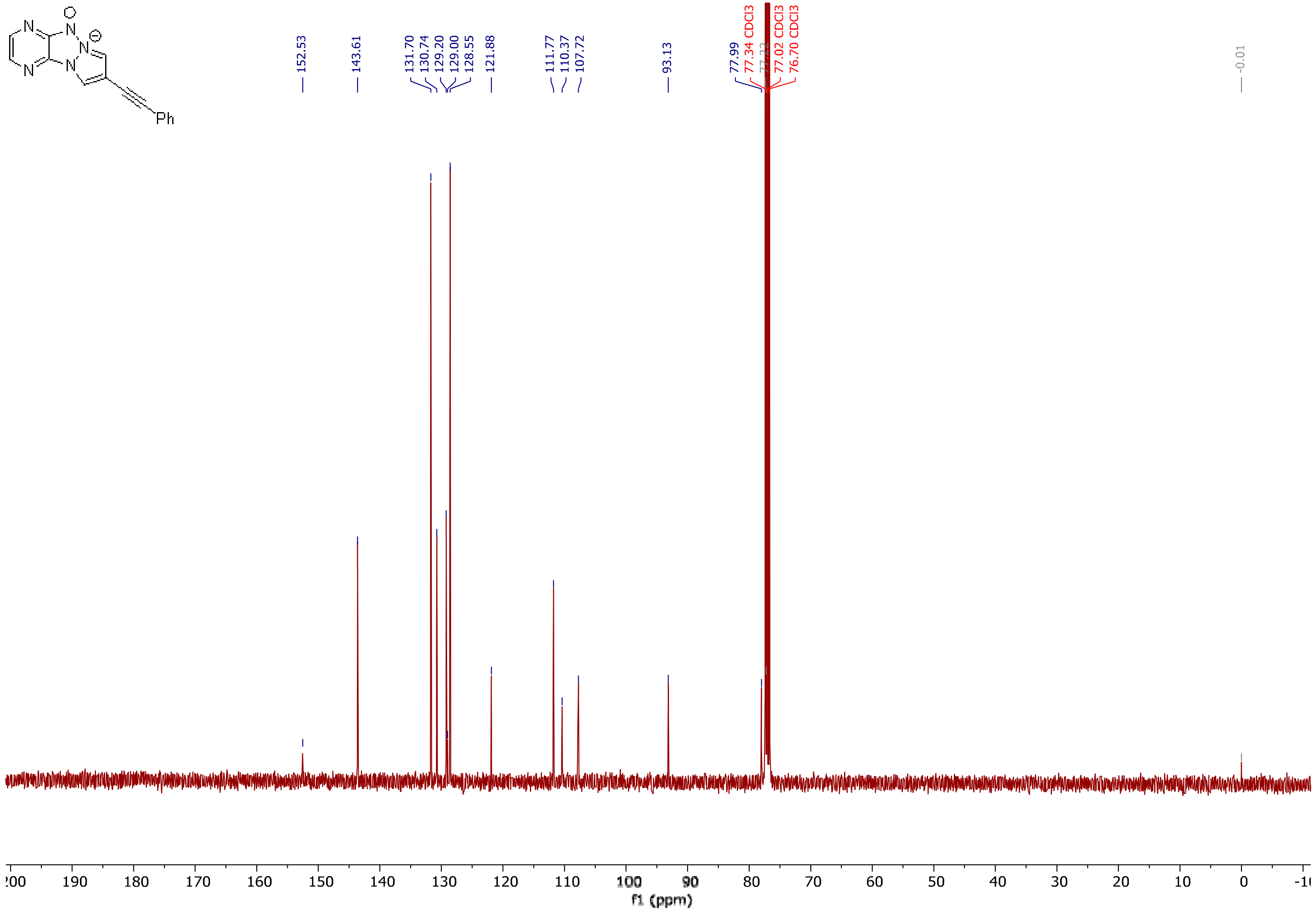

N
O
N
N
N
N
Ph
152.53
143.61
131.70
130.74
129.20
129.00
128.55
121.88
111.77
110.37
107.72
93.13
77.99
77.34 CDCl3
77.02 CDCl3
76.70 CDCl3
-0.01
f1 (ppm)

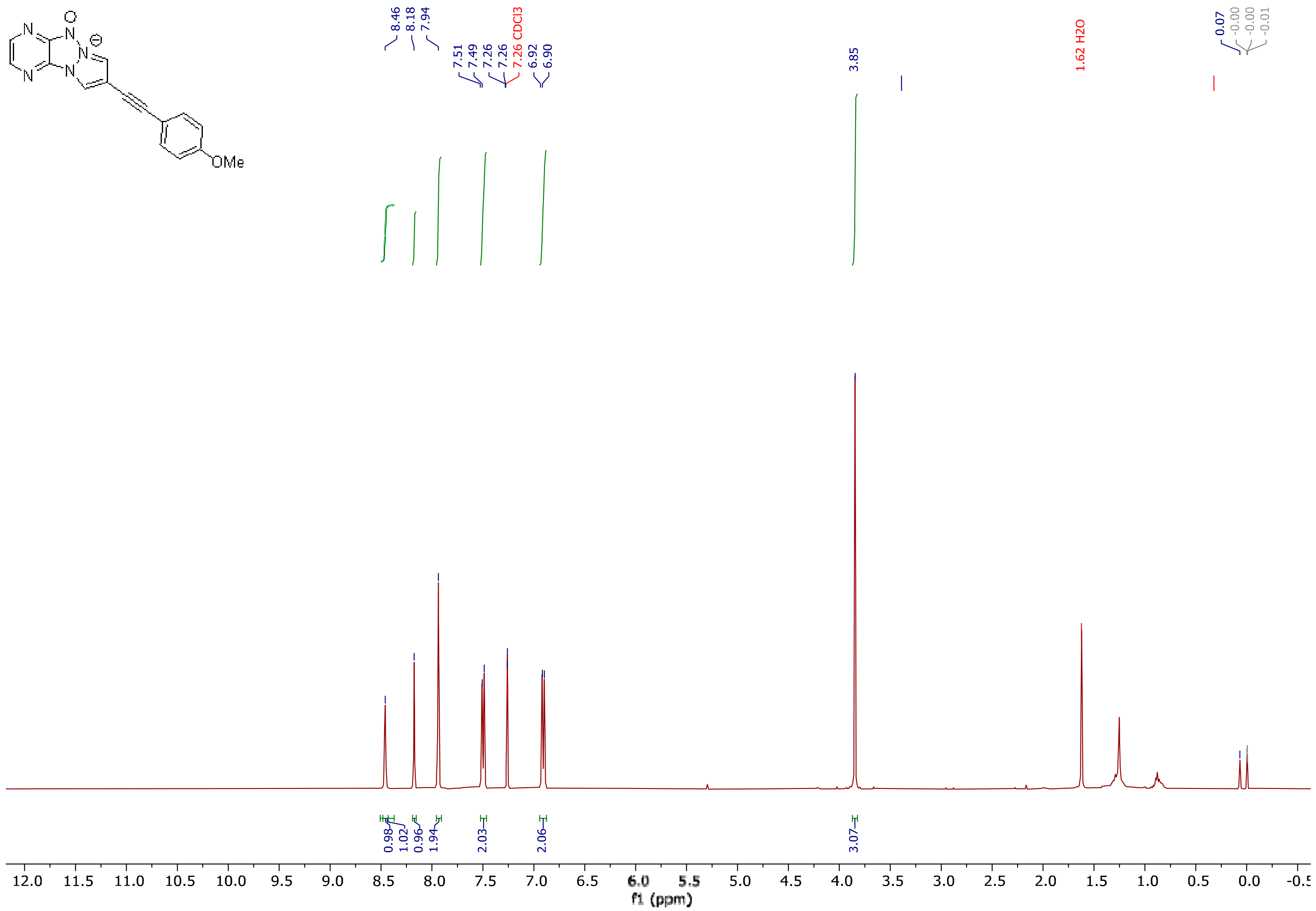

N
O
N
N
N
N
OMe
8.46
8.18
7.94
7.51
7.49
7.26
7.26
7.26 CDCl3
6.92
6.90
3.85
1.62 H2O
0.07
-0.00
-0.00
-0.01
0.98
1.02
0.96
1.94
2.03
2.06
3.07
12.0 11.5 11.0 10.5 10.0 9.5 9.0 8.5 8.0 7.5 7.0 6.5 6.0 5.5 5.0 4.5 4.0 3.5 3.0 2.5 2.0 1.5 1.0 0.5 0.0 -0.5
f1 (ppm)

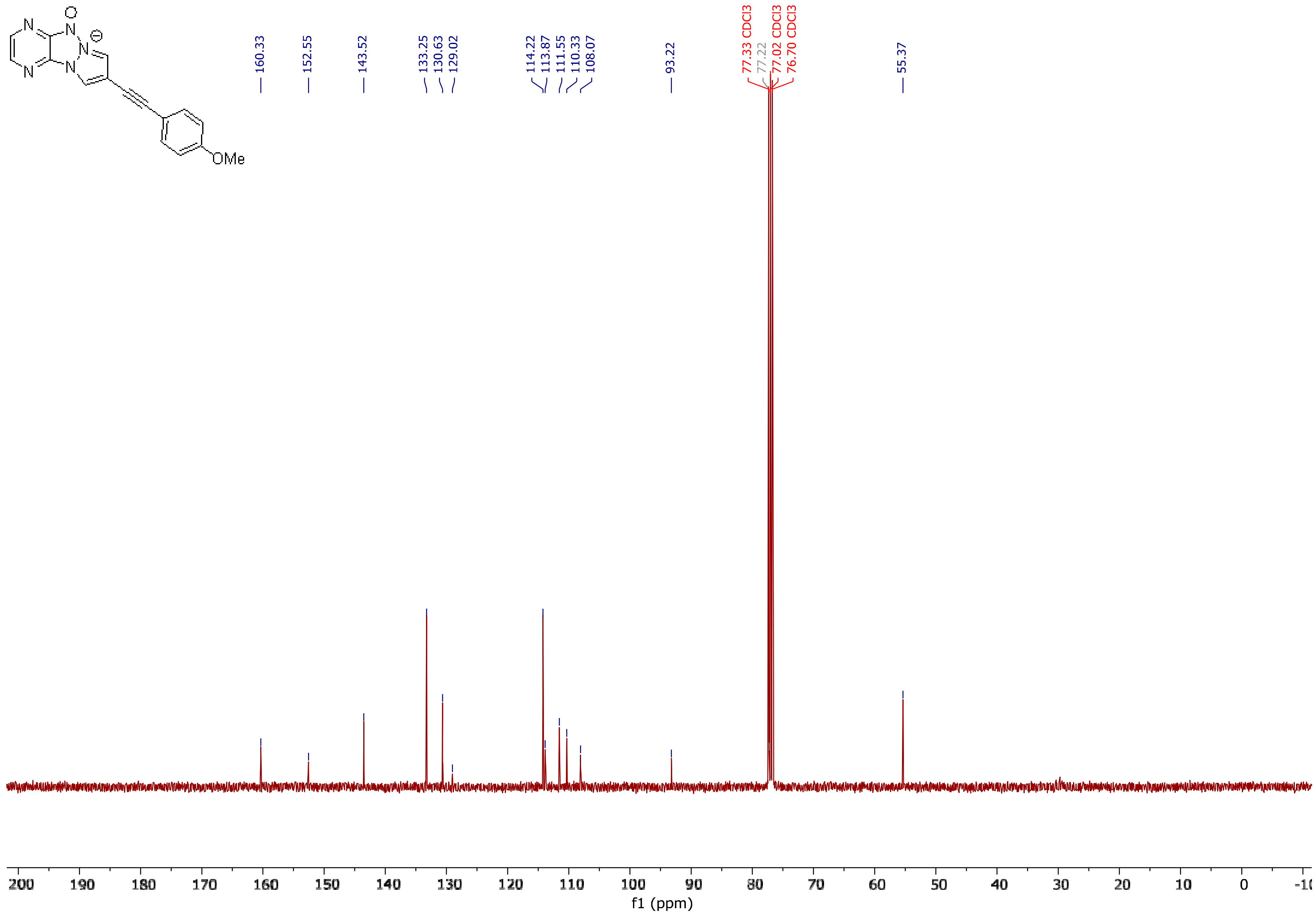

OMe
160.33
152.55
143.52
133.25
130.63
129.02
114.22
113.87
111.55
110.33
108.07
93.22
77.33 CDCl3
77.22
77.02 CDCl3
76.70 CDCl3
55.37
f1 (ppm)

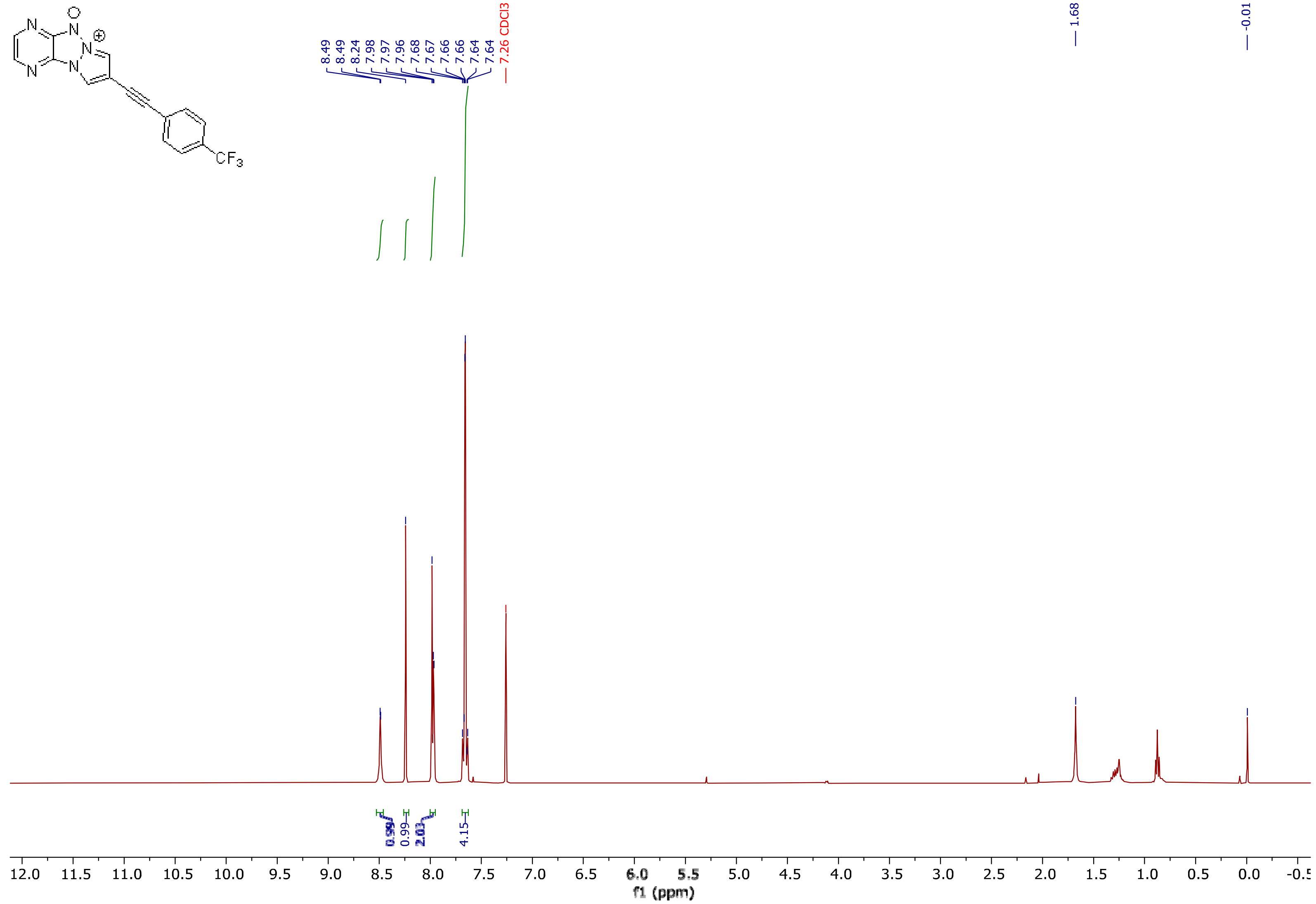

N
O
N
N
⊕
N
N
CF3
8.49
8.49
8.24
7.98
7.97
7.96
7.68
7.67
7.66
7.66
7.64
7.64
7.26 CDCl3
1.68
-0.01
0.99
2.03
4.15
12.0 11.5 11.0 10.5 10.0 9.5 9.0 8.5 8.0 7.5 7.0 6.5 6.0 5.5 5.0 4.5 4.0 3.5 3.0 2.5 2.0 1.5 1.0 0.5 0.0 -0.5
f1 (ppm)

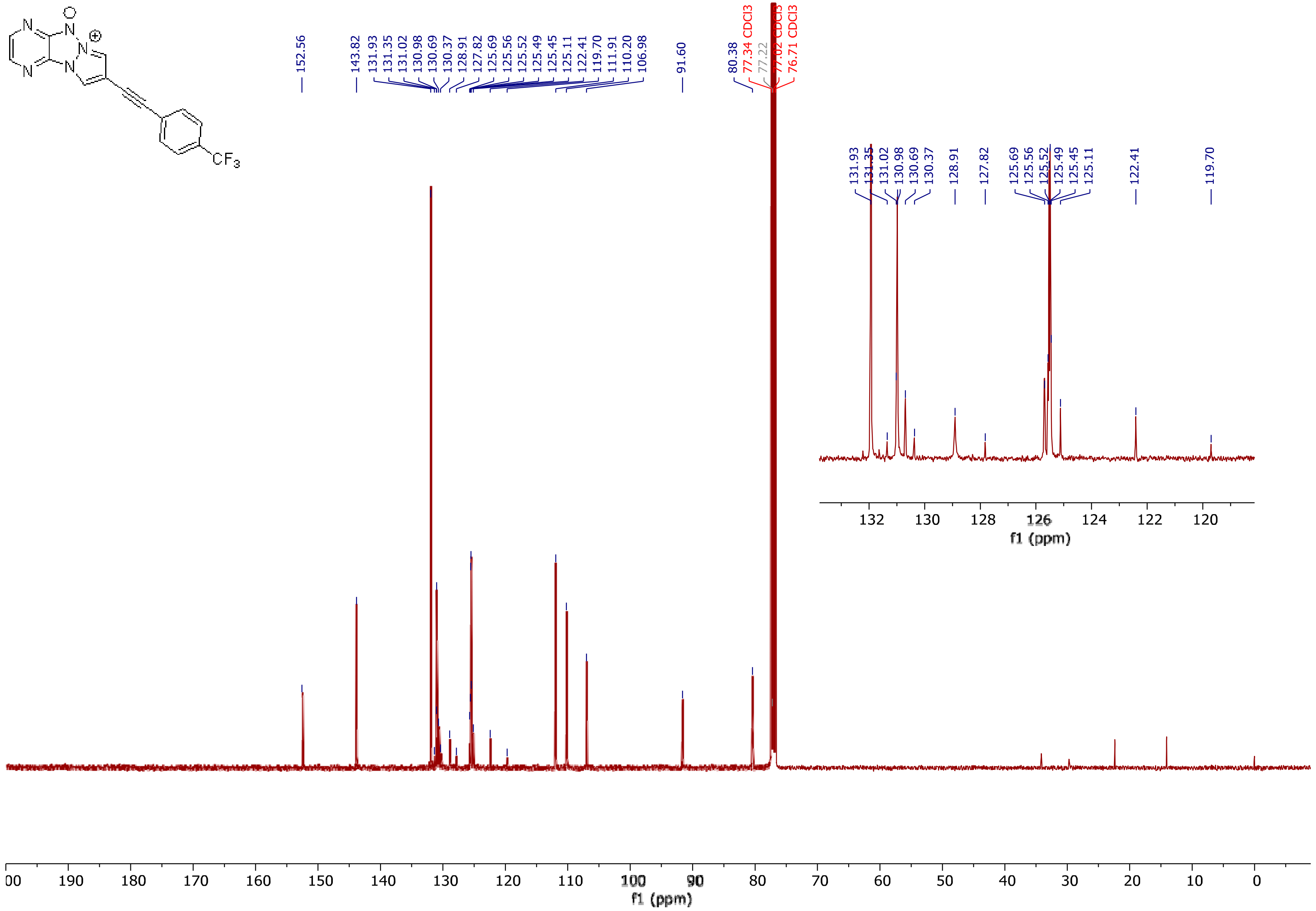

CF3
152.56
143.82
131.93
131.35
131.02
130.98
130.69
130.37
128.91
127.82
125.69
125.56
125.52
125.49
125.45
125.11
122.41
119.70
111.91
110.20
106.98
91.60
80.38
77.34 CDCl3
77.22
77.02 CDCl3
76.71 CDCl3
f1 (ppm)

^{19}F NMR (235 MHz, $CDCl_3$) 8-((4-(trifluoromethyl)phenyl)ethynyl)pyrazolo[1',2':1,2][1,2,3]triazolo[4,5-*b*]pyrazin-6-ium-5- ide **4c**

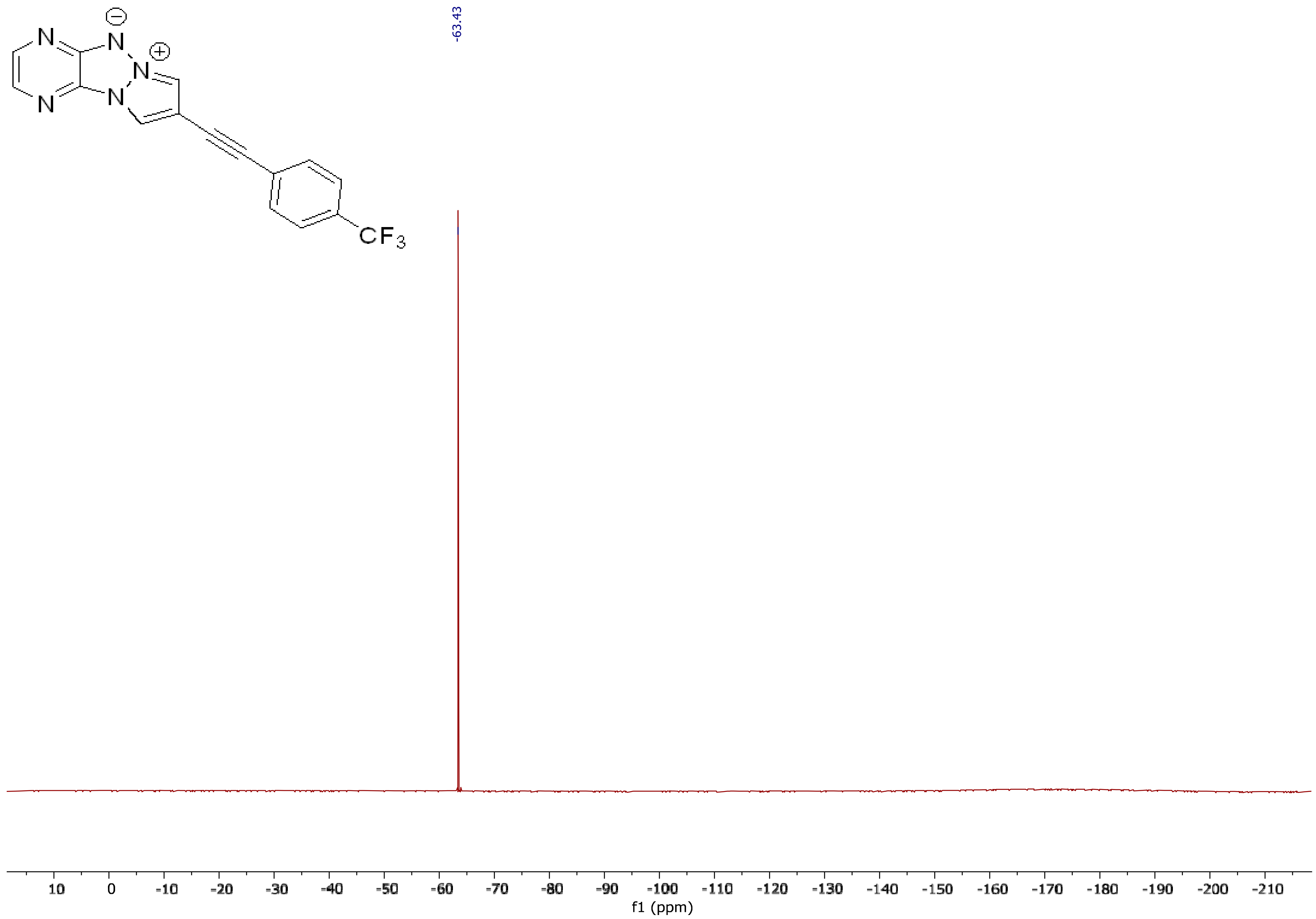

^{1}H NMR (400 MHz, $CDCl_3$) spectrum of 8-((trimethylsilyl)ethynyl)pyrazolo[1',2':1,2][1,2,3]triazolo[4,5-*b*]pyrazin-6-ium-5-ide **4d**

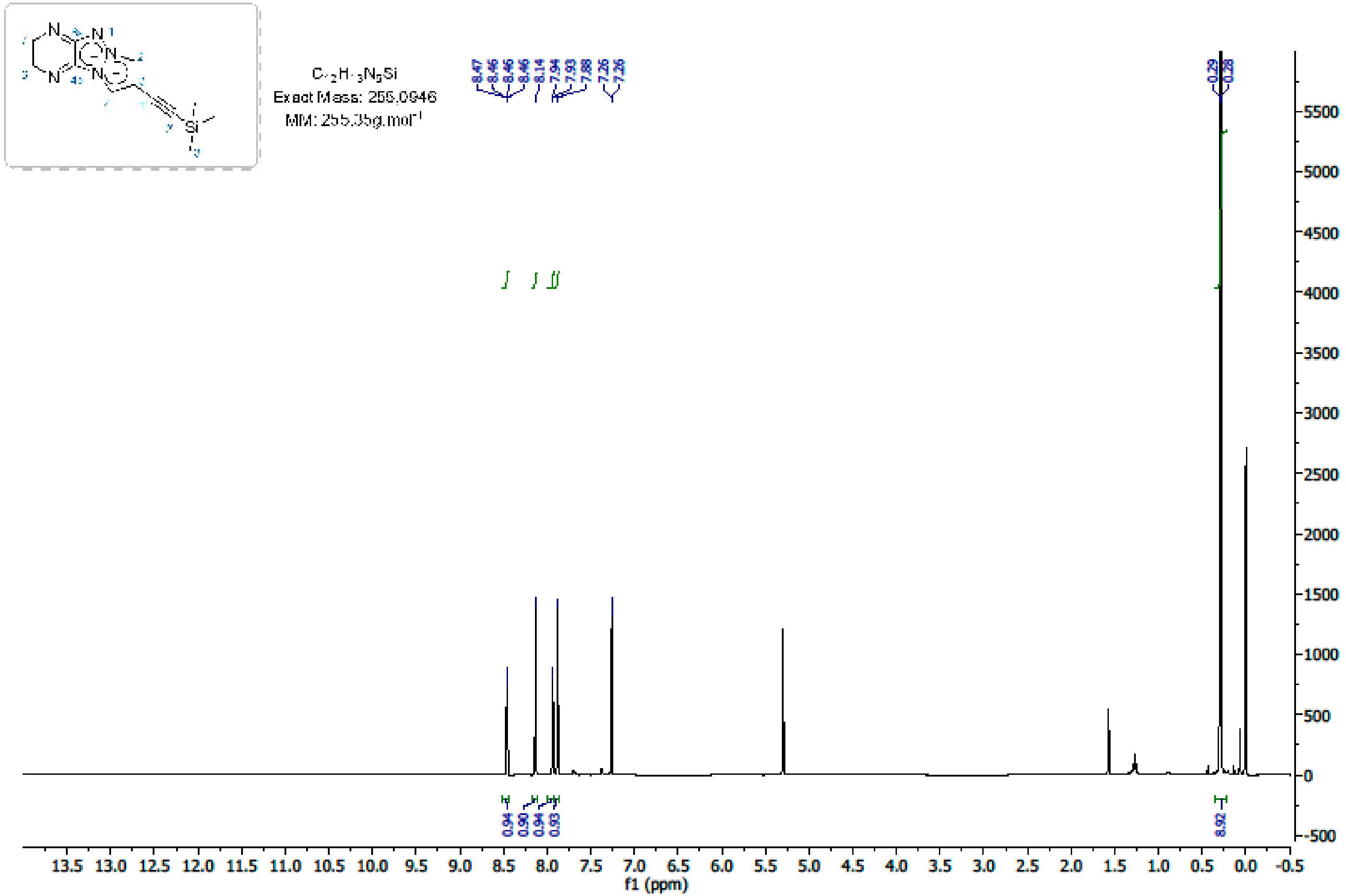

^{13}C NMR (101 MHz, $CDCl_3$) spectrum of 8-((trimethylsilyl)ethynyl)pyrazolo[1',2':1,2][1,2,3]triazolo[4,5-*b*]pyrazin-6-ium-5-ide **4d**

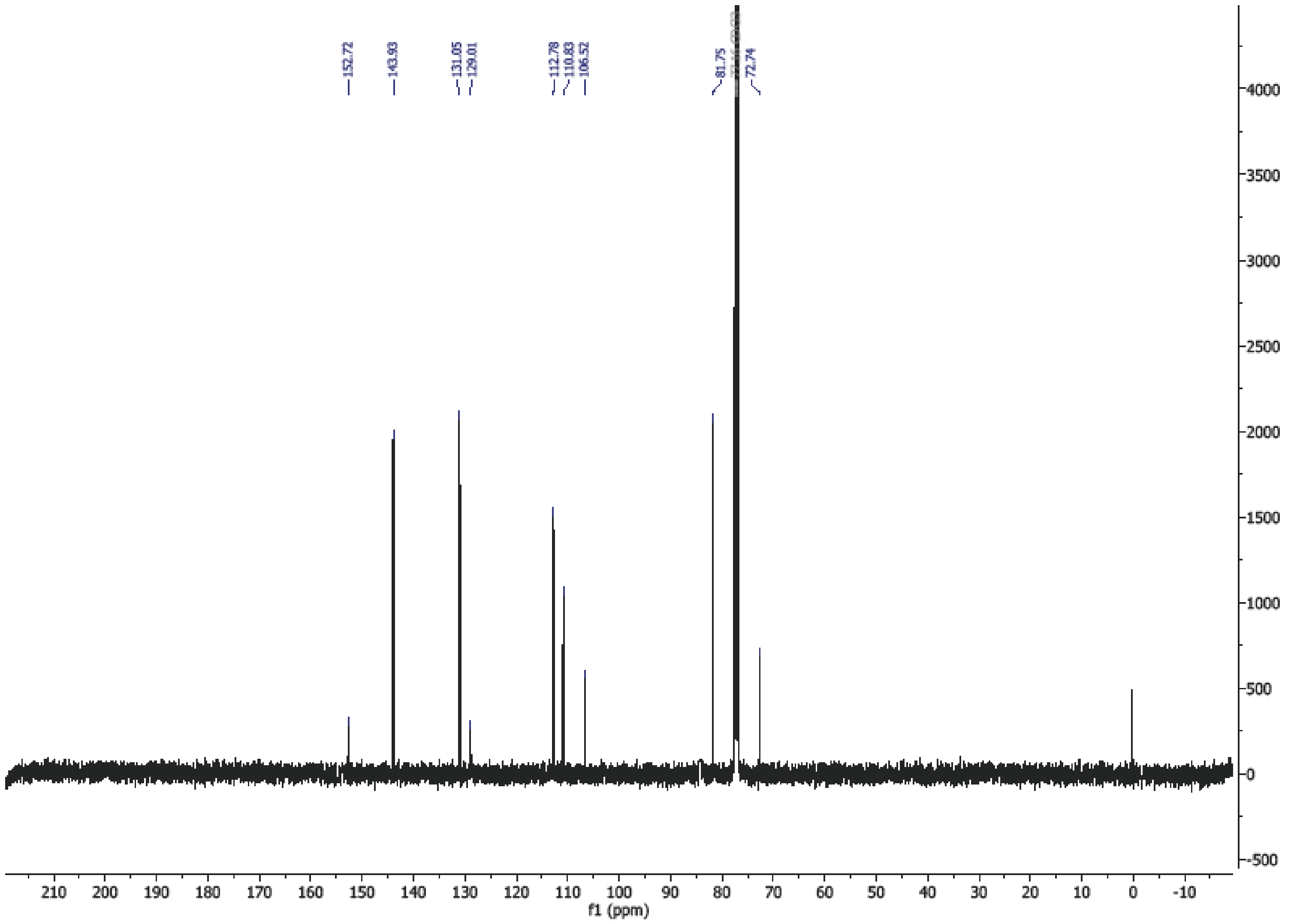

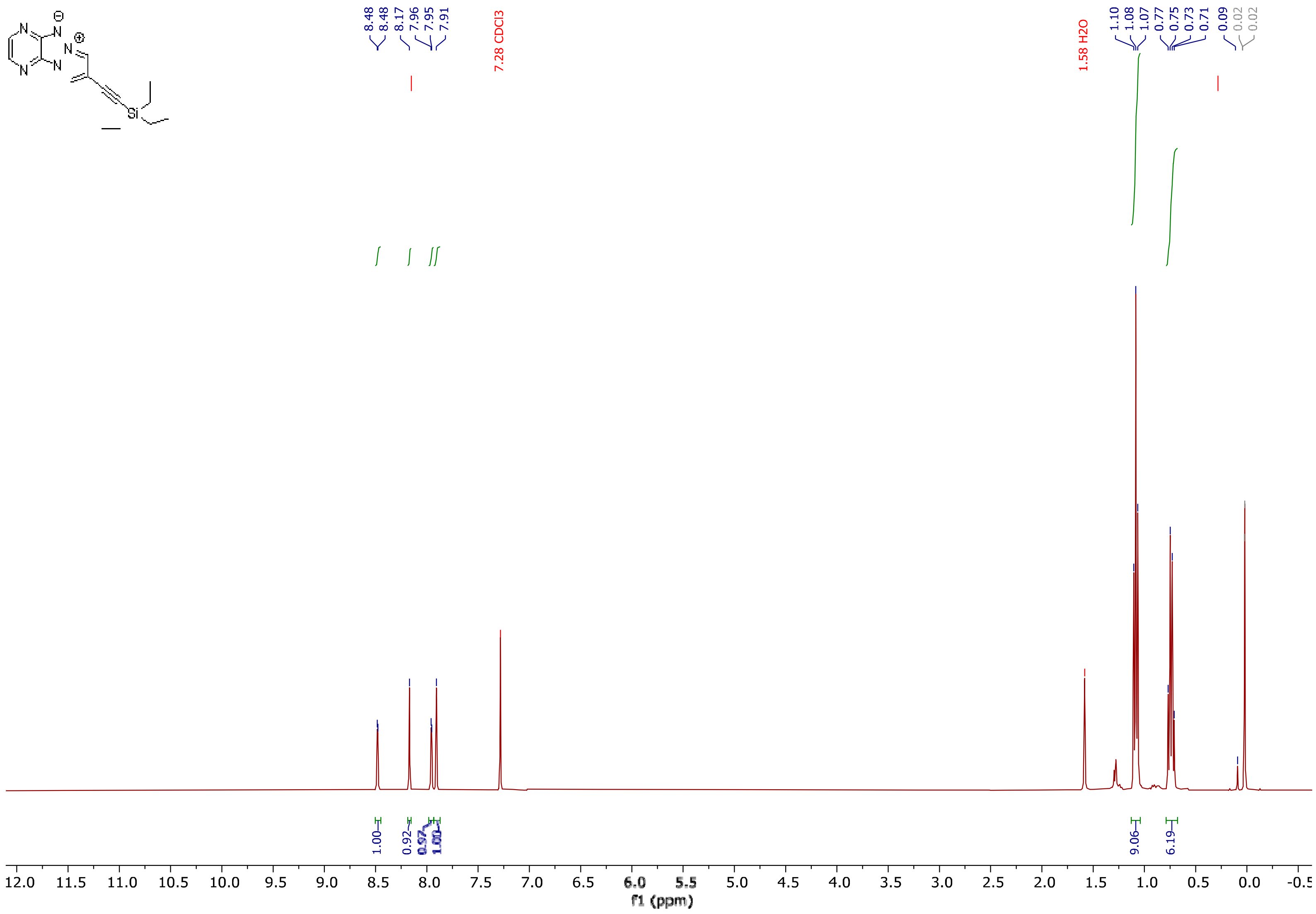

N
N
N
N
N
Si
8.48
8.48
8.17
7.96
7.95
7.91
7.28 CDCl3
1.58 H2O
1.10
1.08
1.07
0.77
0.75
0.73
0.71
0.09
0.02
0.02
1.00
0.92
0.97
1.00
9.06
6.19
12.0 11.5 11.0 10.5 10.0 9.5 9.0 8.5 8.0 7.5 7.0 6.5 6.0 5.5 5.0 4.5 4.0 3.5 3.0 2.5 2.0 1.5 1.0 0.5 0.0 -0.5
f1 (ppm)

^{13}C NMR (101 MHz, $CDCl_3$) spectrum of 8-((triethylsilyl)ethynyl)pyrazolo[1',2':1,2][1,2,3]triazolo[4,5-*b*]pyrazin-6-ium-5-ide **4e**

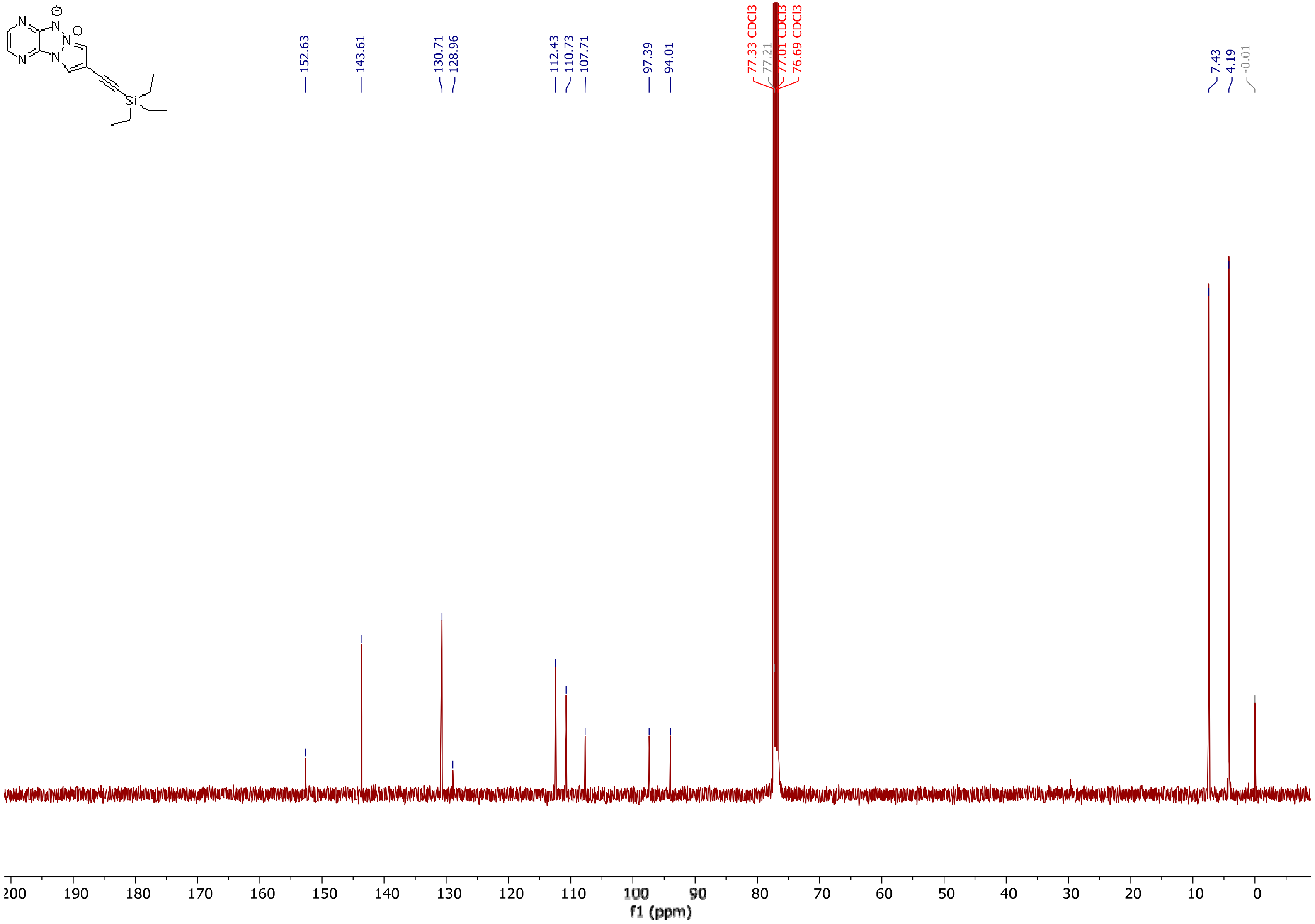

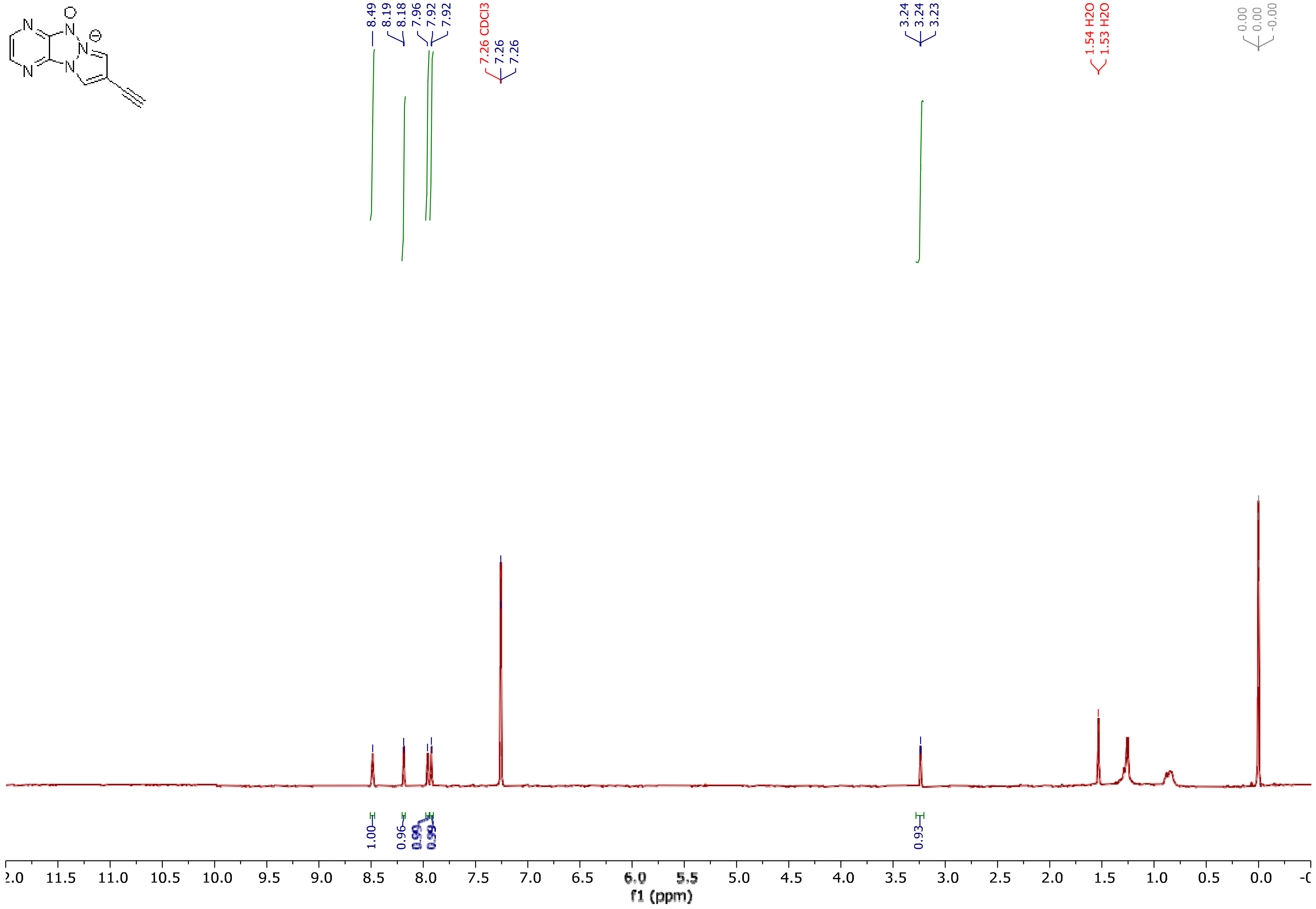

8.49
8.19
8.18
7.96
7.92
7.92
7.26 CDCl3
7.26
7.26
3.24
3.24
3.23
1.54 H2O
1.53 H2O
0.00
0.00
-0.00
1.00
0.96
0.93
f1 (ppm)

^{13}C NMR (101 MHz, $CDCl_3$) spectrum of 8-ethynylpyrazolo[1',2':1,2][1,2,3]triazolo[4,5-*b*]pyrazin-6-ium-5-ide **4f**

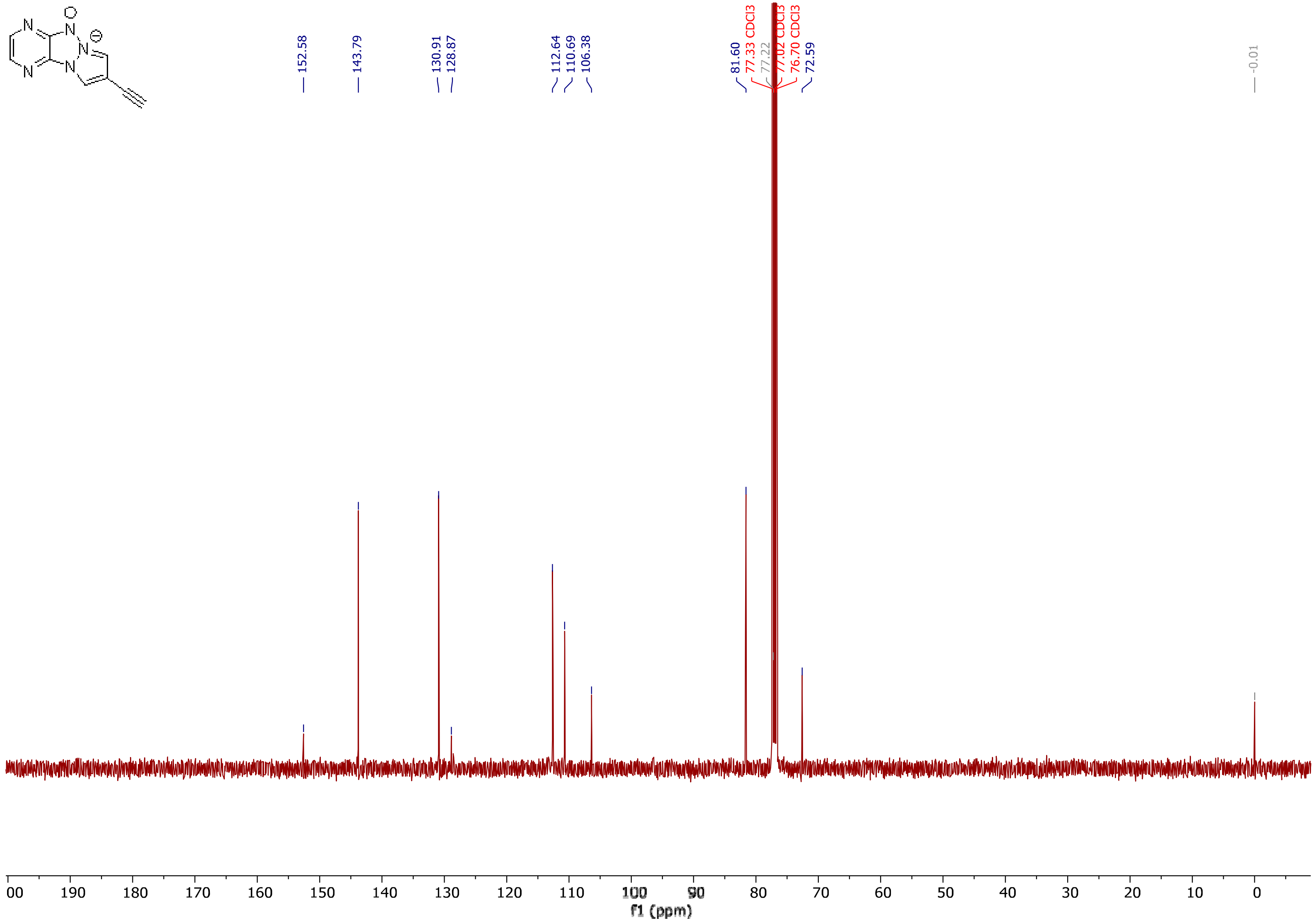

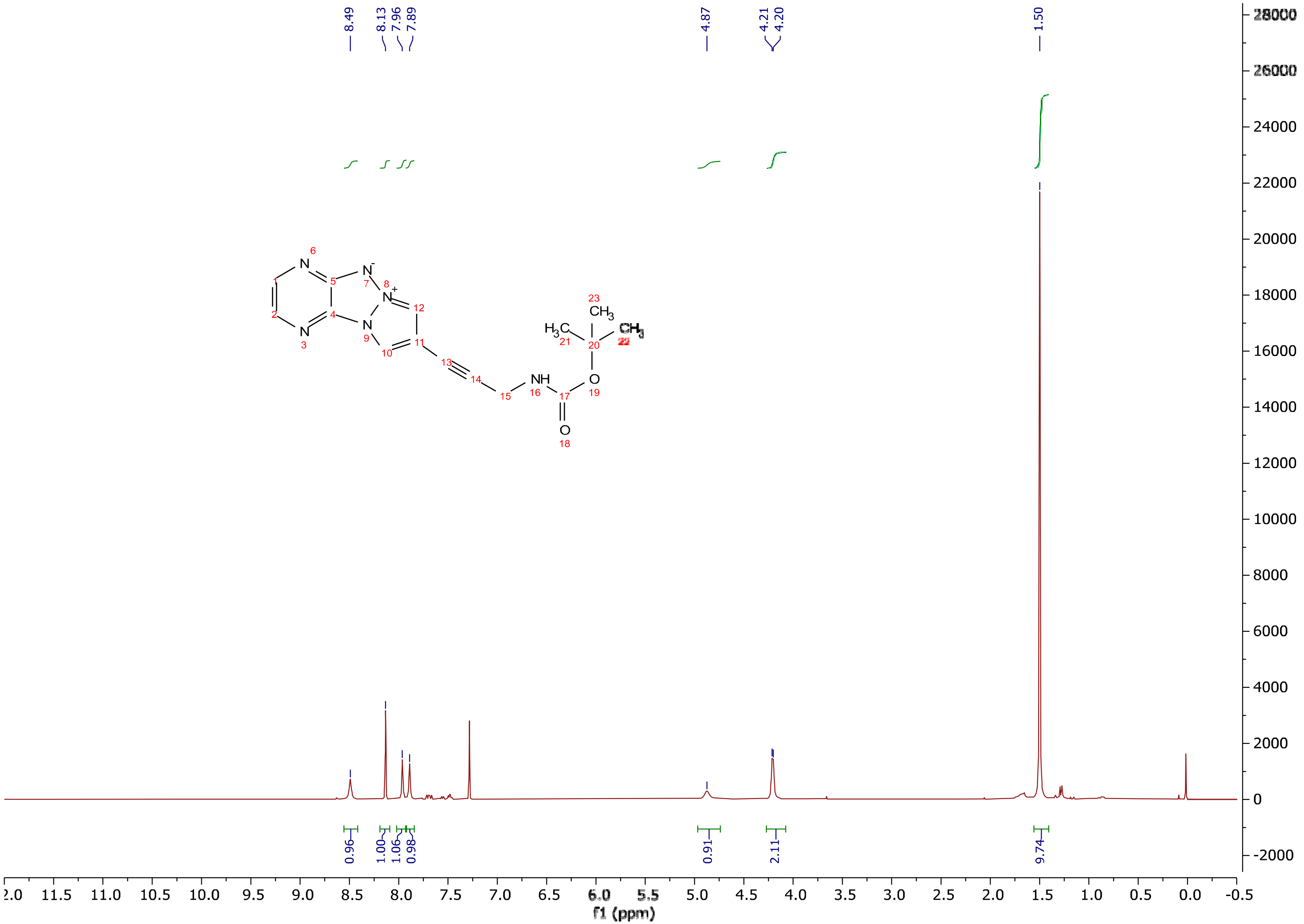

8.49
8.13
7.96
7.89
4.87
4.21
4.20
1.50
0.96
1.00
1.06
0.98
0.91
2.11
9.74
f1 (ppm)

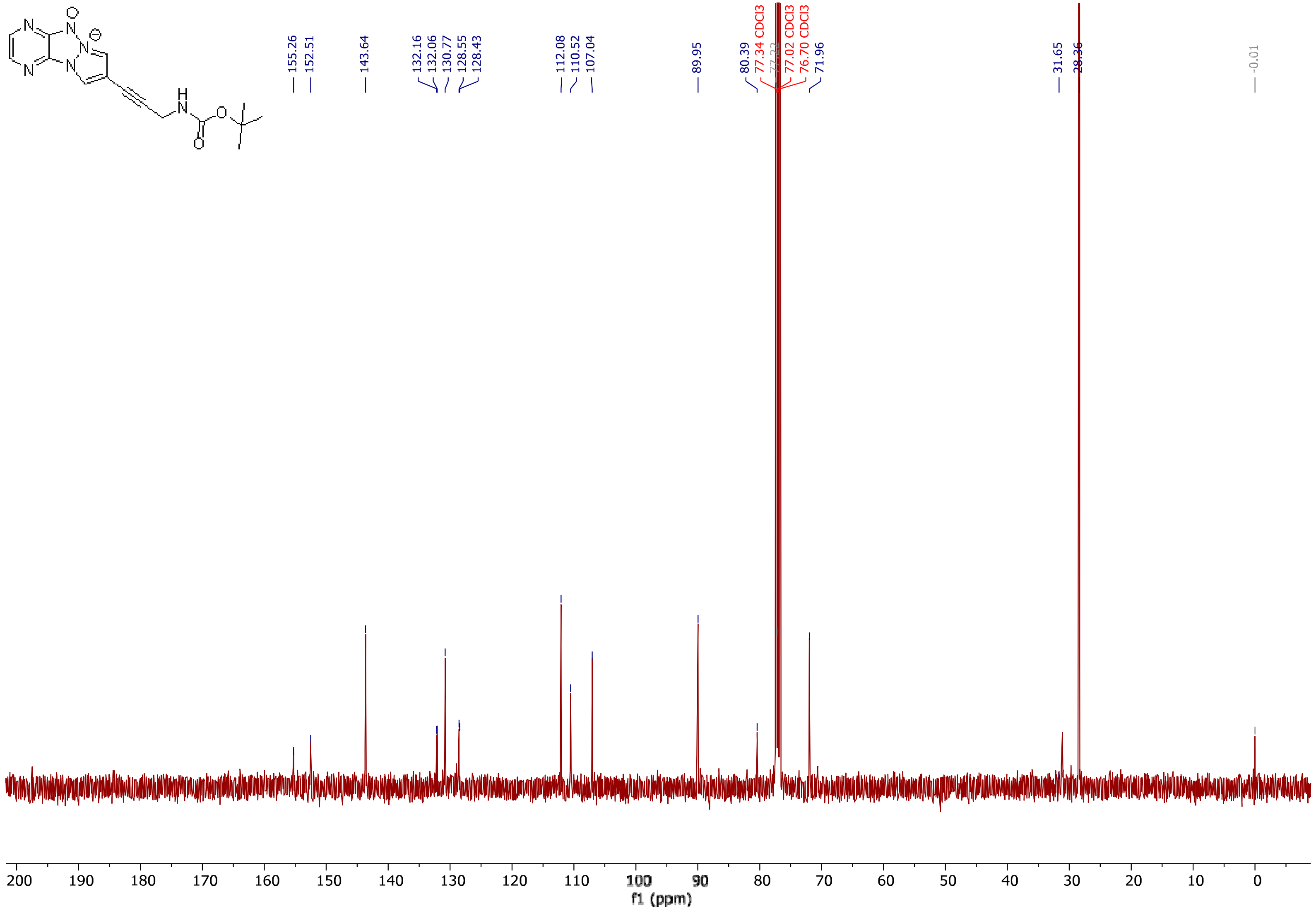
155.26
152.51
143.64
132.16
132.06
130.77
128.55
128.43
112.08
110.52
107.04
89.95
80.39
77.34 CDCl3
77.02 CDCl3
76.70 CDCl3
71.96
31.65
28.36
-0.01
200
190
180
170
160
150
140
130
120
110
100
90
80
70
60
50
40
30
20
10
0
f1 (ppm)

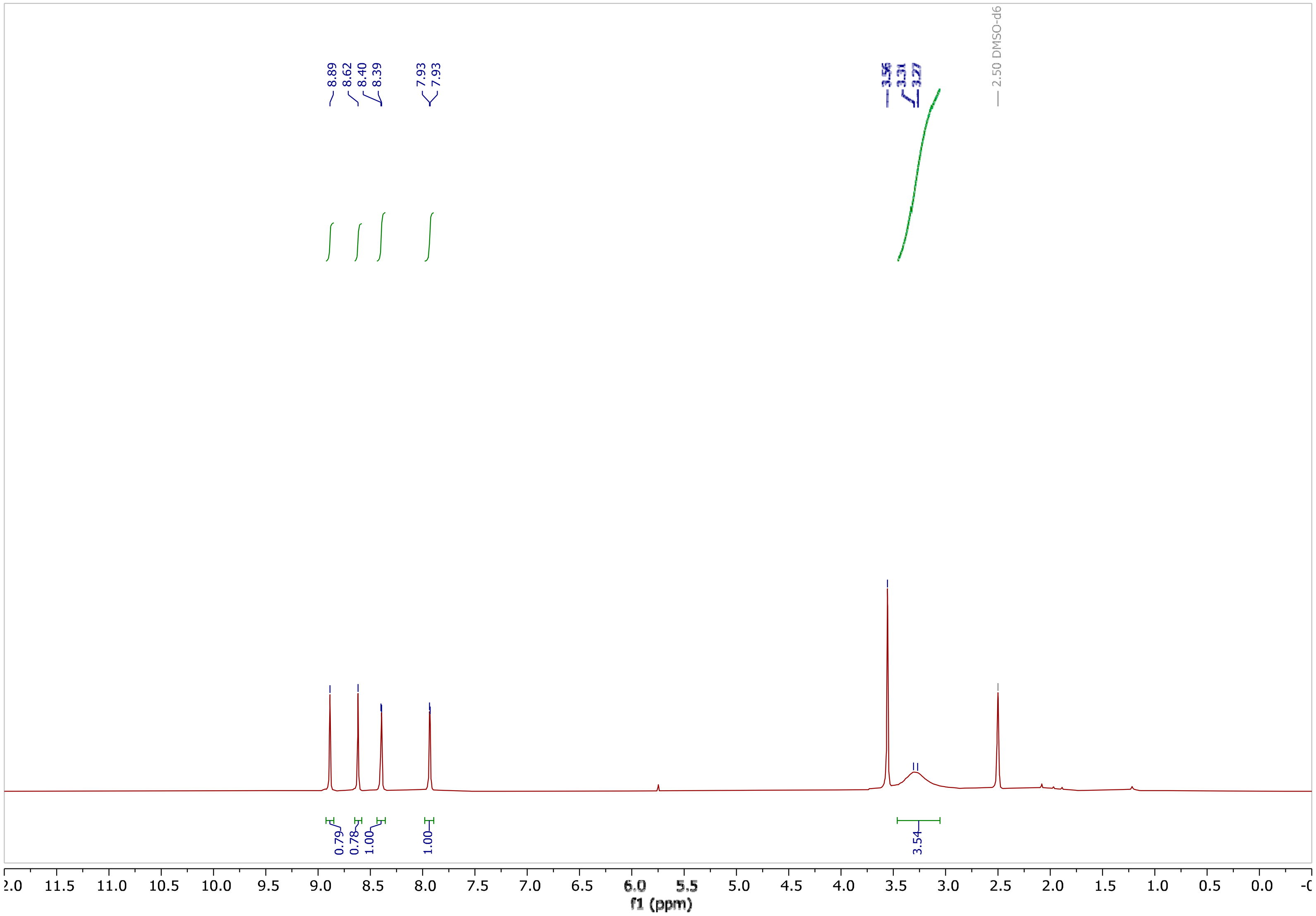

8.89
8.62
8.40
8.39
7.93
7.93
3.56
3.31
3.27
2.50 DMSO-d6
0.79
0.78
1.00
1.00
3.54
f1 (ppm)

^{13}C NMR (101 MHz, Acetone *d6*) spectrum of 8-(3-aminoprop-1-yn-1-yl)pyrazolo[1',2':1,2][1,2,3]triazolo[4,5-*b*]pyrazin-6-ium-5-ide **4h**

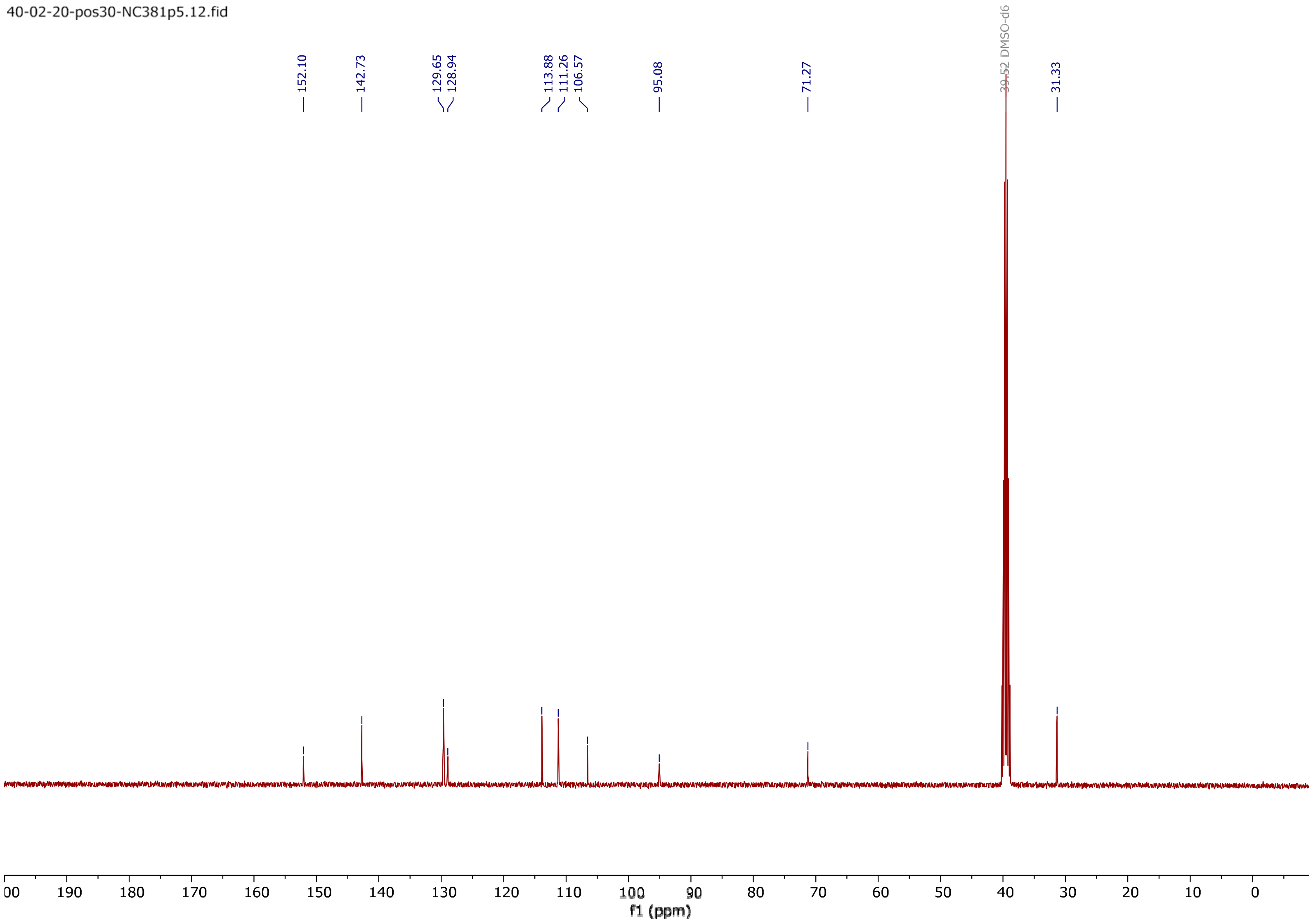

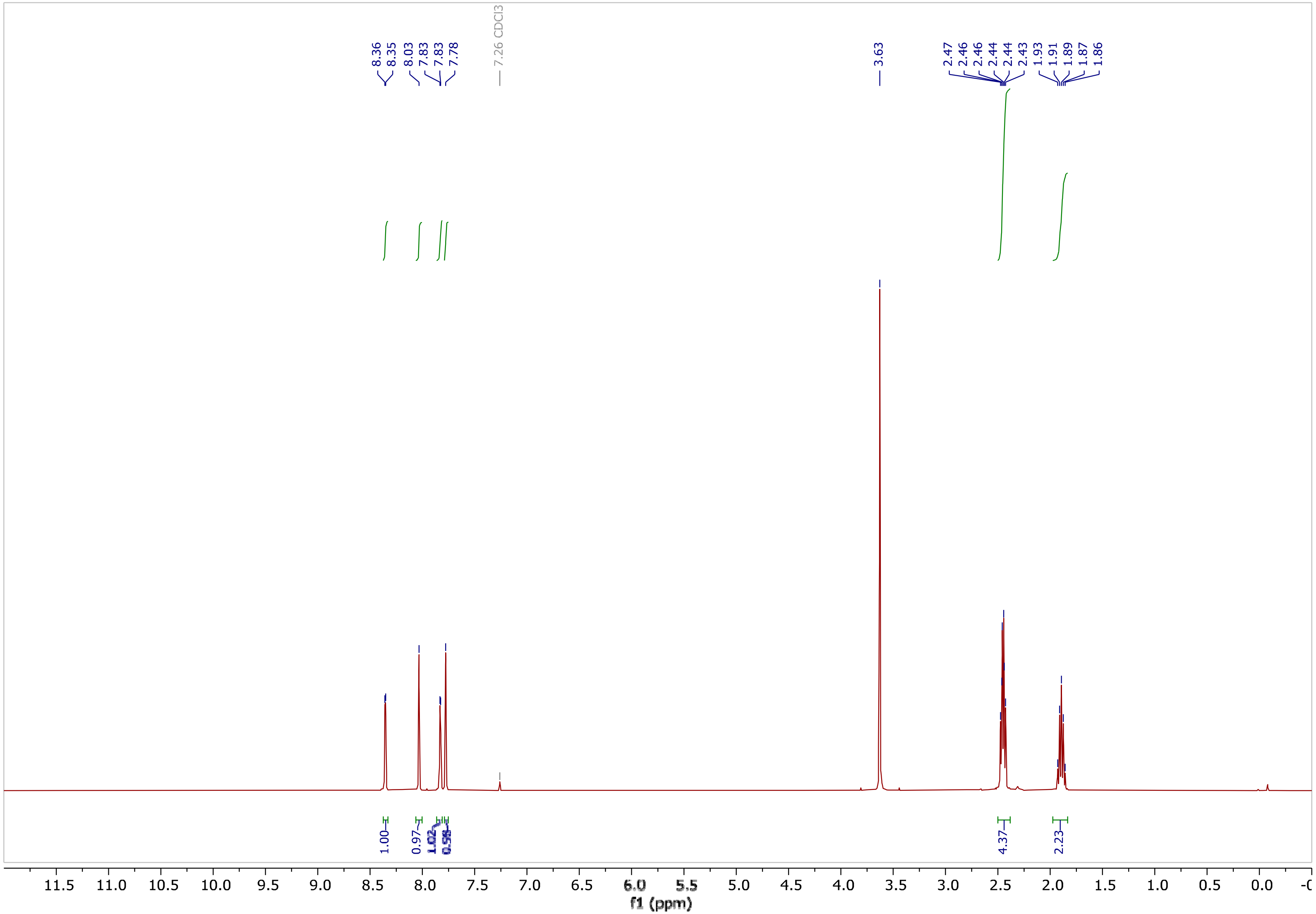
8.36
8.35
8.03
7.83
7.83
7.78
7.26 CDCl3
3.63
2.47
2.46
2.46
2.44
2.44
2.43
1.93
1.91
1.89
1.87
1.86
1.00
0.97
4.37
2.23
f1 (ppm)

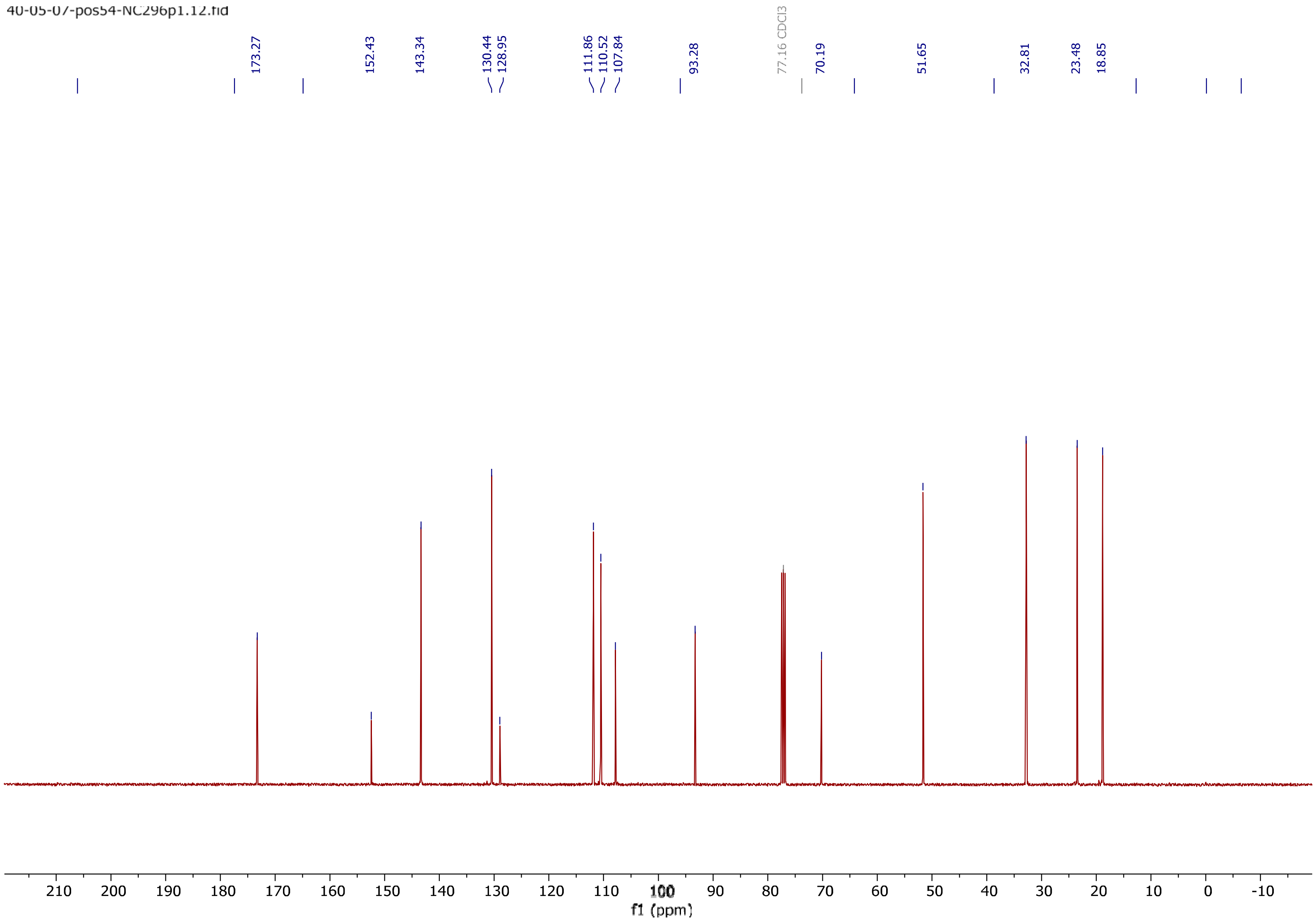
40-05-07-pos54-NC296p1.12.fid
173.27
152.43
143.34
130.44
128.95
111.86
110.52
107.84
93.28
77.16 CDCl3
70.19
51.65
32.81
23.48
18.85
210 200 190 180 170 160 150 140 130 120 110 100 90 80 70 60 50 40 30 20 10 0 -10
f1 (ppm)

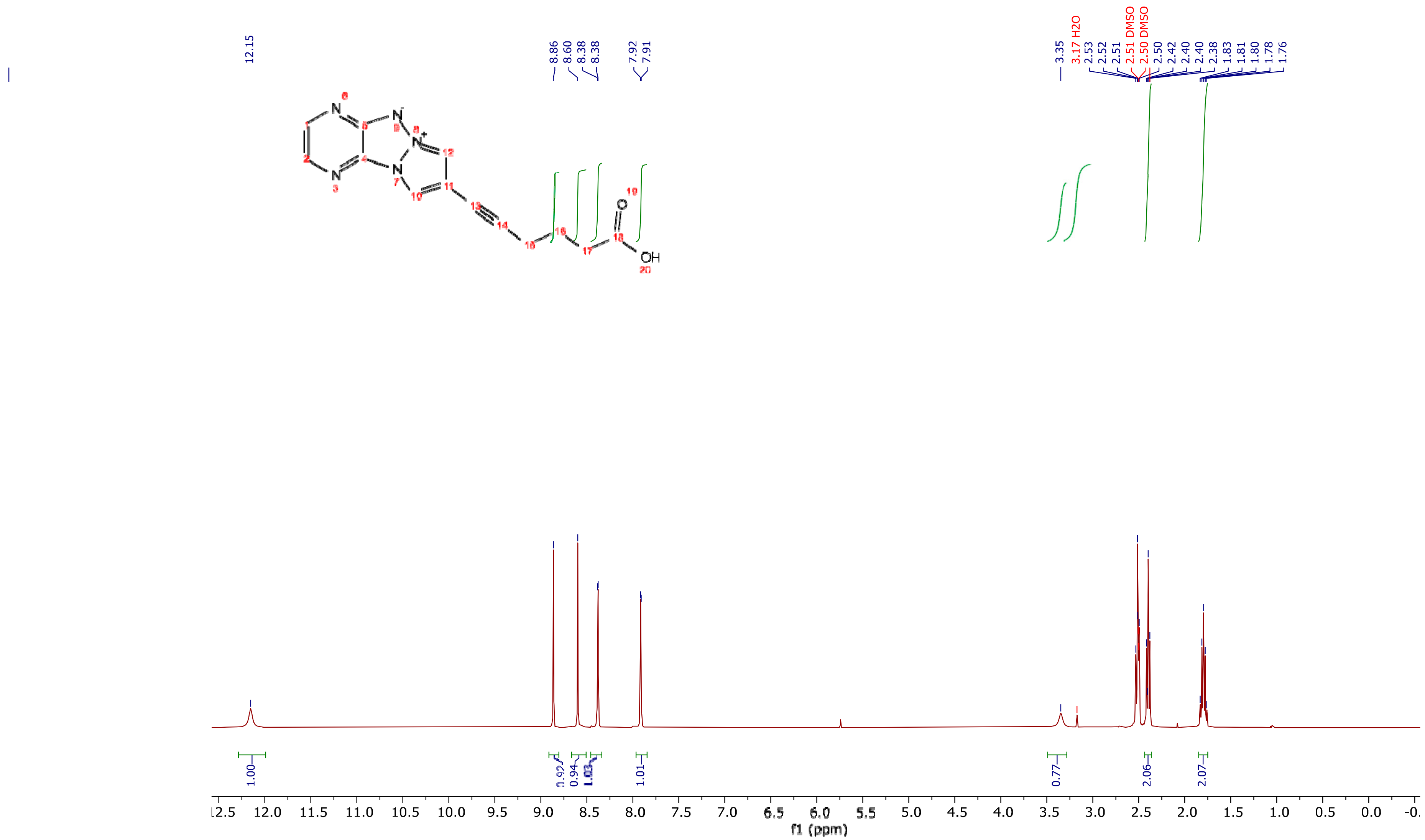
12.15
8.86
8.60
8.38
8.38
7.92
7.91
3.35
3.17 H2O
2.53
2.52
2.51
2.51 DMSO
2.50 DMSO
2.50
2.42
2.40
2.40
2.38
1.83
1.81
1.80
1.78
1.76
OH
1.00
0.94
1.01
0.77
2.06
2.07
f1 (ppm)

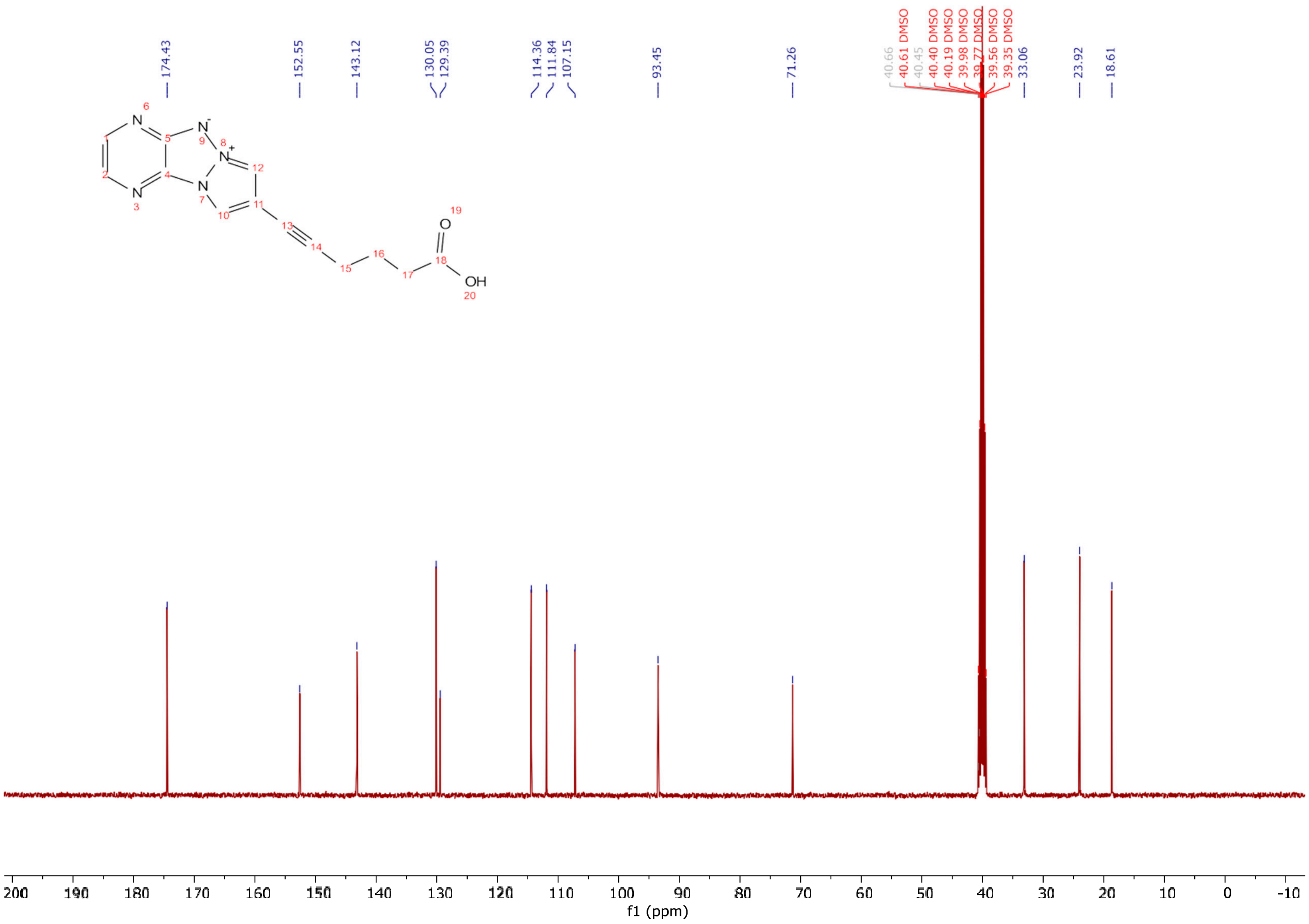
174.43
152.55
143.12
130.05
129.39
114.36
111.84
107.15
93.45
71.26
40.66
40.61 DMSO
40.45
40.40 DMSO
40.19 DMSO
39.98 DMSO
39.77 DMSO
39.56 DMSO
39.35 DMSO
33.06
23.92
18.61
f1 (ppm)

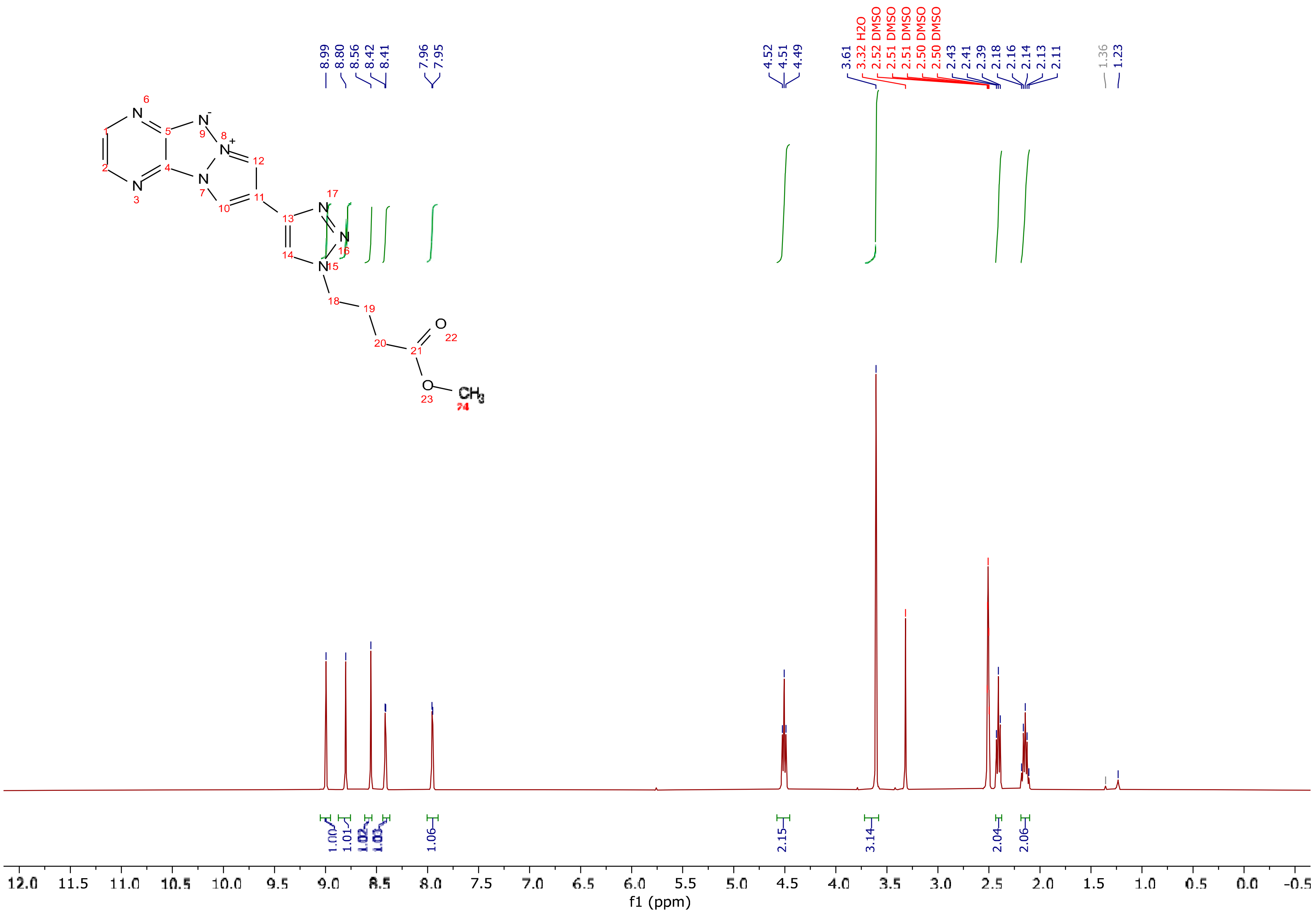

8.99
8.80
8.56
8.42
8.41
7.96
7.95
4.52
4.51
4.49
3.61
3.32 H2O
2.52 DMSO
2.51 DMSO
2.51 DMSO
2.50 DMSO
2.50 DMSO
2.43
2.41
2.39
2.18
2.16
2.14
2.13
2.11
1.36
1.23
1.00
1.01
1.06
2.15
3.14
2.04
2.06
f1 (ppm)

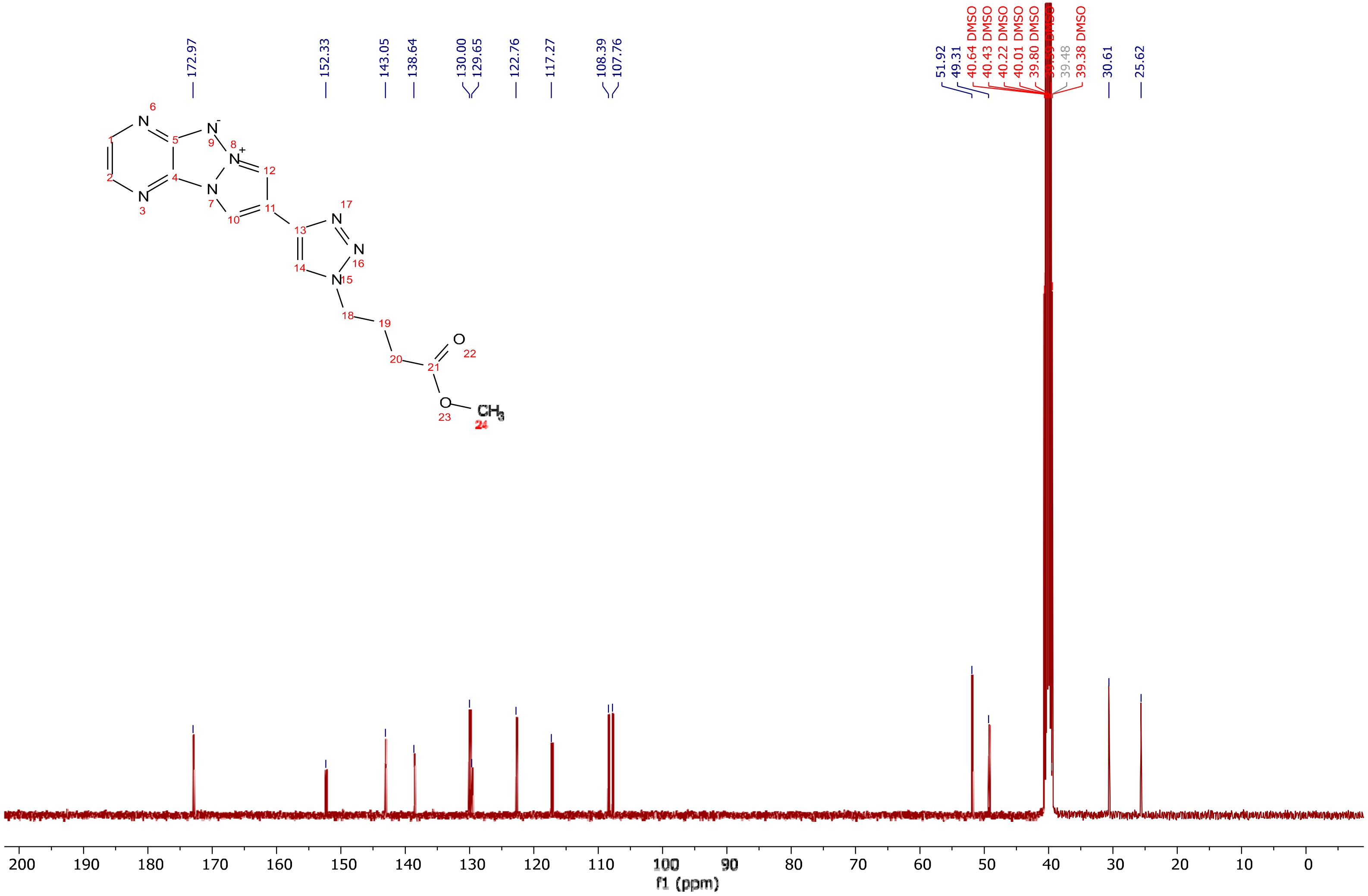

172.97
152.33
143.05
138.64
130.00
129.65
122.76
117.27
108.39
107.76
51.92
49.31
40.64 DMSO
40.43 DMSO
40.22 DMSO
40.01 DMSO
39.80 DMSO
39.48
39.38 DMSO
30.61
25.62
200 190 180 170 160 150 140 130 120 110 100 90 80 70 60 50 40 30 20 10 0
f1 (ppm)

Photophysical measurements : absorption, excitation and emission spectra

Absorption, excitation and emission spectrum of **2a** in DCM

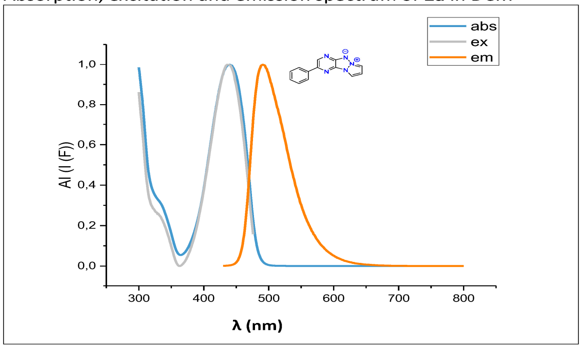

Absorption, excitation and emission spectrum of **2b** in DCM

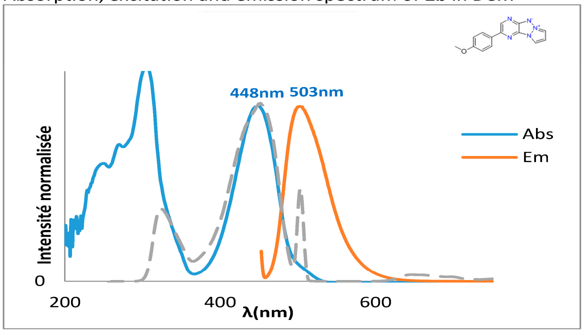

Absorption, excitation and emission spectrum of **2c** in DCM

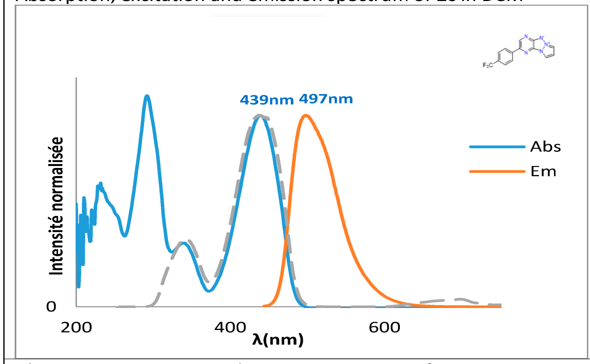

Absorption, excitation and emission spectrum of **2d** in DCM

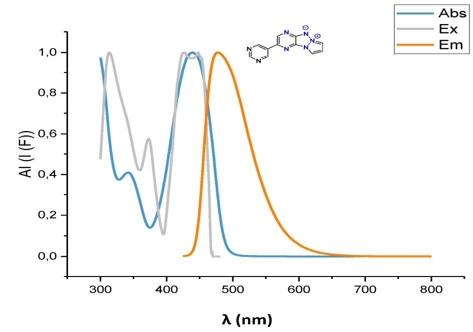

Absorption, excitation and emission spectrum of **3a** in DCM

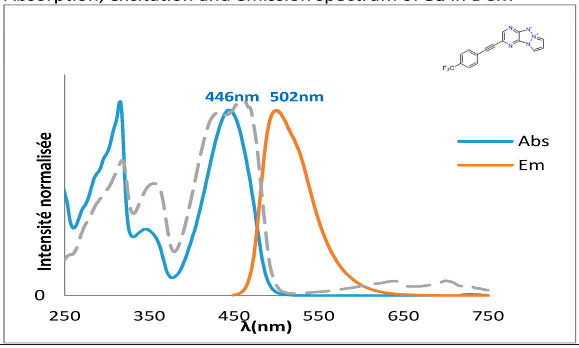

Absorption, excitation and emission spectrum of **3a** in DMSO

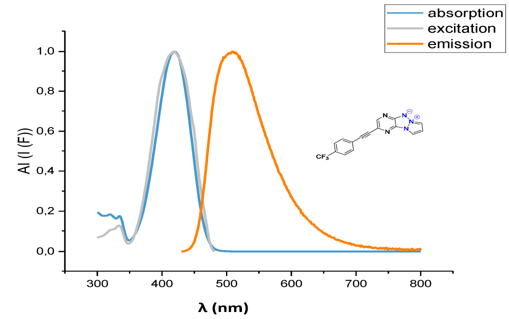

Absorption, excitation and emission spectrum of **4a** in DCM

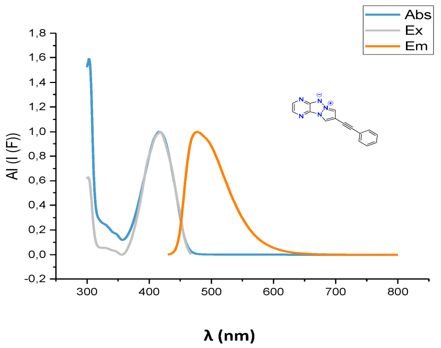

Absorption, excitation and emission spectrum of **4b** in DCM

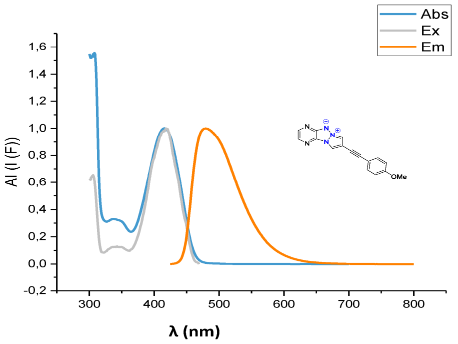

Absorption, excitation and emission spectrum of **4c** in DMSO

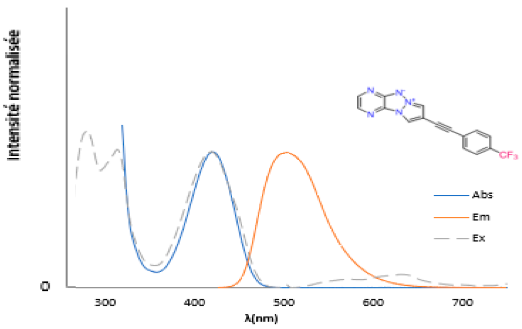

Absorption, excitation and emission spectrum of **4e** in DCM

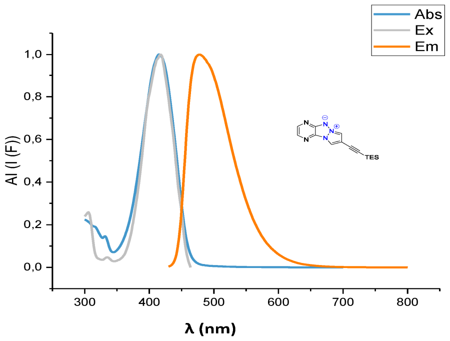

Absorption, excitation and emission spectrum of **4f** in DCM

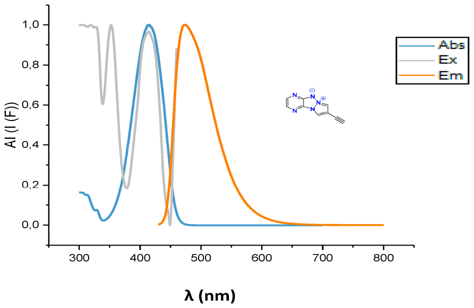

Absorption, excitation and emission spectrum of **4g** in DCM

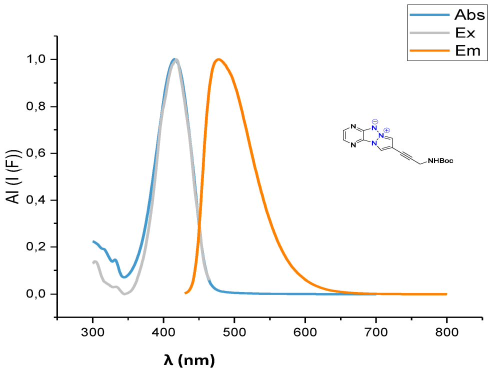

Absorption, excitation and emission spectrum of **4i** in DCM

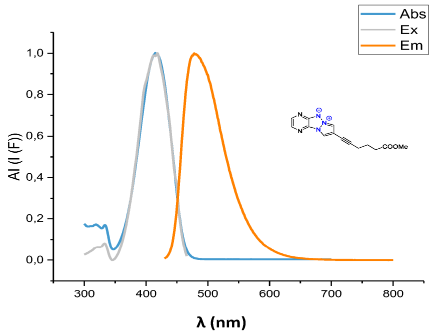

Absorption, excitation and emission spectrum of **4i** in DMSO

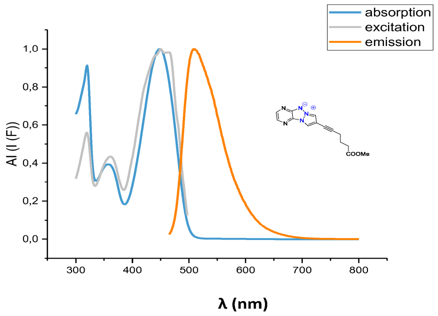

Absorption, excitation and emission spectrum of **4h** in DCM

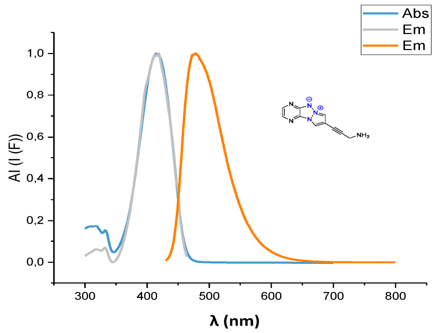

Absorption, excitation and emission spectrum of **4j** in DCM

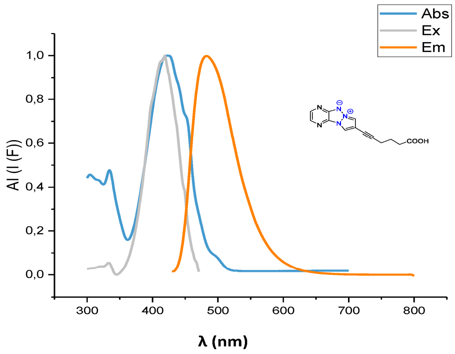

Absorption, excitation and emission spectrum of **5** in DCM

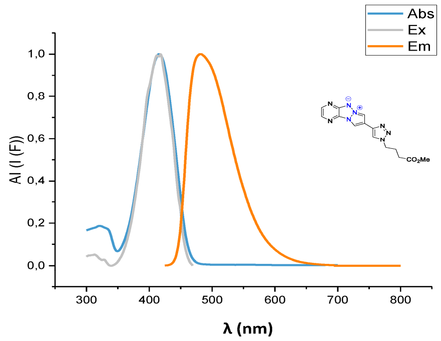

Supplement: Supplementary file 1 [file ijms-22-06645-s001.zip › ijms-1245969-supplementary.pdf]
